# The chromatin reader Dido3 is a regulator of the gene network that controls B cell differentiation

**DOI:** 10.1186/s13578-025-01394-x

**Published:** 2025-04-26

**Authors:** Fernando Gutiérrez del Burgo, María Ángeles García-López, Tirso Pons, Enrique Vázquez de Luis, Carlos Martínez-A, Ricardo Villares

**Affiliations:** 1 Centro Nacional de Biotecnología/CSIC, Darwin 3, Cantoblanco, E‐28049 Madrid, Spain; 2 Centro Nacional de Investigaciones Cardiovasculares, Instituto de Salud Carlos III, Melchor Fernández Almagro 3, 28029 Madrid, Spain

## Abstract

The development of hematopoietic cell lineages is a highly complex process governed by a delicate interplay of various transcription factors. The expression of these factors is influenced, in part, by epigenetic signatures that define each stage of cell differentiation. In particular, the formation of B lymphocytes depends on the sequential silencing of stemness genes and the balanced expression of interdependent transcription factors, along with DNA rearrangement. We have investigated the impact of Dido3 deficiency, a protein involved in chromatin status readout, on B cell differentiation within the hematopoietic compartment of mice. Our findings revealed significant impairments in the successive stages of B cell development. The absence of Dido3 resulted in remarkable alterations in the expression of essential transcription factors and differentiation markers, which are crucial for orchestrating the differentiation process. Additionally, the somatic recombination process, responsible for generation of antigen receptor diversity, was also adversely affected. These observations highlight the vital role of epigenetic regulation, particularly the involvement of Dido3, in ensuring proper B cell differentiation. This study reveals new mechanisms underlying disruptive alterations, deepening our understanding of hematopoiesis and may potentially lead to insights that aid in the development of therapeutic interventions for disorders involving aberrant B cell development.

**Supplementary Information:**

The online version contains supplementary material available at 10.1186/s13578-025-01394-x.

## Introduction

B cells originate from hematopoietic stem cells (HSC) through a highly regulated process that involves the activation or silencing of key transcription factors and concurrent DNA recombination events. HSC can differentiate into multipotent progenitors (MPP) which transition through various lymphoid progenitor cell stages, including lymphoid primed multi-potential progenitor (LMPP), common lymphoid progenitor (CLP), prepro-B, pro-B, pre-B, and immature B cells [25].

During the CLP stage, somatic rearrangements occur between the diversity (DH) and joining (JH) gene segments of the heavy chain (HC) locus, marking the initiation of B cell lineage commitment [2]. CLPs have the potential to differentiate into multiple cell types, such as B lymphocytes, T lymphocytes, NK cells, and dendritic cells [29, 33]. In mice, the upregulation of B220 expression in a CLP subpopulation facilitates their differentiation into prepro-B cells. Prepro-B cells, characterized by the expression of the tyrosine kinase AA4.1 (CD93), are committed to B cell differentiation [44]. The transition to the early pro-B state is marked by CD19 expression and the completion of DH-JH rearrangement [38]. IL7R signaling contributes to the survival and transient proliferation of early pro-B cells, as well as the sequential reorganization of immunoglobulin (Ig) genes [8]. The productive rearrangement of VH heavy chain variable segments with DJH (VH-DJH) in the late pro-B stage is crucial for forming a functional pre-B cell receptor (pre-BCR), which is essential for further survival and differentiation [32].

Epigenetic mechanisms, including post-translational histone modifications, play a crucial role in controlling chromatin structure, DNA accessibility, and gene expression in a cell type-specific manner. Proteins that specifically recognize and interpret these modifications are involved in development, differentiation, and tumorigenesis [7].

*Dido1* is a highly conserved gene found in various organisms. Its shortest encoded protein isoform, DIDO1, contains nuclear localization and export signals (NLS, NES), as well as a plant Zn-finger homeodomain (PHD). DIDO1 interacts with chromatin primarily through di/trimethylated lysine of histone H3 (H3K4me2/3) and functions as a chromatin reader [49]. Although the exact protein complexes associated with DIDO1 remain unknown, all three natural isoforms contain both the PHD and NLS domains. DIDO2, the least abundant isoform, includes a transcription elongation factor S-II central domain (TFIIS2M) required for the nucleolytic activity of RNA polymerase II, as well as a *Spen* paralog and ortholog C-terminal (SPOC) domain, which acts as a reader of RNA polymerase C-terminal domain (CTD) [4], with transcriptional corepressor activity. DIDO3, the main isoform, encompasses all the domains present in DIDO2, along with a coiled-coil (CC) region that likely has a structural role. Additionally, DIDO3 contains low complexity regions rich in proline, alanine, and arginine residues.

Attempts to delete *Dido1* have resulted in cell lethality. N-terminal truncation leads to the expression of a shorter protein driven by an internal promoter, which is associated with aneuploidy, centrosome amplification, and centromere-localized breaks [22]. Mice with the *Dido1*ΔNT mutation exhibit myeloid neoplasms, and alterations in DIDO1 are linked to myelodysplastic syndrome in humans [43]. Deletion of the 3' terminal exon (ΔE16) of *Dido1* results in a truncated form of Dido3, leading to embryonic lethality by gestation day 8.5 [14]. Embryonic stem cells derived from these embryos lacking the C-terminal of Dido3 fail to differentiate properly but retain their self-renewal capacity [16, 18]

Given the embryonic lethality associated with *Dido1*ΔE16 deletion, we investigated the impact of Dido3 deficiency on the hematopoietic lineage using mice that carry a floxed 3'-terminal exon 16 (flE16) of *Dido1* [36] and a Cre recombinase gene under the control of the *Vav1* promoter. In these mice, the B cell population showed significant reductions, suggesting that Dido3 plays a crucial role in chromatin remodeling necessary for B cell differentiation, thereby influencing developmental cell plasticity and lineage fate decisions.

## Materials and methods

### Mice

A mouse strain bearing an 3'-terminal exon 16 (E16) flanked by *loxP* sites was generated as described elsewhere [18, 36] and interbred with Cre^SOX2^ mice, producing mice heterozygous for the E16 deletion (Supplementary Fig. 1A). Mice carrying one functional and one mutated allele, and transgenic for Vav1-cre, were crossed with mice homozygous for both the floxed allele of *Dido1* and to obtain experimental and control littermates.

Hematopoietic populations were isolated from the offspring. All experiments were carried out with animals between 6 and 10 weeks of age, sex- and age-matched in each case.

### Flow cytometry and cell sorting

Bone marrow cells from each sample were diluted in a solution of 0.5% PBS-BSA, 0.065% NaN_3_. To avoid nonspecific antibody binding, samples were blocked with Fc-Block (CD16/32; BD Pharmingen). Antibodies used were from eBioscience (B220-eF450, CD3-biotin, CD5-percpCy5.5, CD19-AlexaFluor700, CD93-APC, CD127 (IL7R)-PeCy7, Sca1APC), Beckman Coulter (B220-biotin, B220-PE, CD4-biotin, CD8-PE, CD8-biotin, cKit-APC, Gr1-biotin, Ly6C-biotin), BioLegend (CD4-PeCy7, CD11b-PeCy7), and BD Pharmingen (CD11b-biotin, CD21-APC, CD23-PE, CD43biotin, IgG1-PE, IgG3-PE, Ly6G-PE, Nk1.1-APC). Biotinylated antibodies were visualized with streptavidin-eF450 (eBioscience), streptavidin-PE (Southern Biotech) or streptavidin-PE-CF594 (eBioscience). Cell death was monitored using annexin V-APC (Immunostep) and DAPI. In *in vivo* assays, the cell cycle was analyzed by measuring BrdU incorporation into cell DNA 18 h after intraperitoneal injection of 200 mg BrdU (Invitrogen). Cells were labeled following the protocol for the BrdU Staining Kit for flow cytometry (Invitrogen). Analytical cytometric data was acquired using a Gallios cytometer (Beckman Coulter). Flow cytometry analyses were performed using FlowJo v10.6.0.

The same combination of antibodies used in flow cytometry was applied to mouse bone marrow cells to select the target population in staining buffer (PBS containing 10% FBS). These cells were then sorted using a BD FACSAria Fusion cytometer and the data were analyzed with BD FACSDiva version 8.0.1.

### In vitro differentiation of bone marrow cells

Bone marrow cells from control (WT) and *Dido1*∆E16 mice (lin^–^: CD3, CD11b, CD19, CD45R (B220), Ly6G/C (Gr1), TER119), were purified with the EasySep Mouse Hematopoietic Progenitor Cell Isolation kit (STEMCELL Technologies) according to the manufacturer's protocol. The isolated cells were grown in a semi-solid methyl cellulose matrix for the Mouse Hematopoietic CFU Assay in Methocult Media 3630 (STEMCELL Technologies), following the manufacturer's instructions. The medium was supplemented with IL7, which promotes growth of B lymphocyte progenitor cells as pre-B colony-forming units (CFU-pre-B).

### RT-qPCR

RNA was extracted from sorted bone marrow LSK cells (Lin (B220, Ly6G/C (Gr1), TER119, CD3, CD11c, CD11b, F480)^-^), Sca-1^+^, cKit^+^, IL-R7^-^ using the RNeasy Plus Mini Kit (Qiagen) according to the manufacturer’s instructions. The quality of the RNA was assessed by electropherogram (Agilent 2100 Bionalyzer). To synthesize cDNA, the SuperScriptIII First Strand Synthesis System for RT-PCR kit (Invitrogen) was employed, with up to 1 µg total RNA used as the template in a final reaction volume of 20 µl. qPCR reactions were performed in 384-well plates on an ABI PRISM 7900HT system (Applied Biosystems) using SYBR Green qPCR Master Mix (Applied Biosystems). Data acquisition and analysis were conducted using ABI SDS 2.0 software.

### RNA-seq

Pre-B (B220^+^, CD19^+^, IgM^-^) cells extracted from the bone marrow of WT and *Dido1*∆E16 mice were isolated by sorting. RNA was purified using the RNeasy Mini Plus Kit (Qiagen). Samples containing approximately 100 ng of RNA with an Integrity Number (RIN) ≥ 9 were processed for high-throughput sequencing at BGI Genomics (Hong Kong) using the HiSeq 2000 platform.

### ATAC-seq

LSK cells of WT and *Dido1*∆E16 mice were isolated by sorting and processed using the DNA Library Prep Kit (Illumina). The tagged DNA was purified with the DNA Clean Concentrator™5 Kit (Zymo Research). The samples were pre-amplified with the Nextera Index kit and the Nextera DNA Library Prep kit (both from Illumina), incorporating index sequences at the ends. DNA from the PCR reaction was purified using the DNA Clean Concentrator™5 Kit. To quantify the tagged DNA, the 1x dsDNA HS Assay kit (Qubit) was utilized. Samples were then enriched for DNA sizes between 200 and 600 bp (corresponding to 1–3 nucleosomes) using AMPure XP magnetic beads (Beckman-Coulter), following the manufacturer's guidelines. For sequencing the tagged DNA, a mixture of libraries was prepared with a balanced proportion of three samples each of WT and *Dido1*ΔE16 LSK cells, each containing approximately 100,000 pmol/l of tagged DNA fragments. High-throughput sequencing was performed by the CNIC Genomics Service on a HiSeq 2500 platform using a Hi-Seq-Rapid PE Flowcell 2x50 kit.

### ChIP-seq

LSK cells from bone marrow of WT and *Dido1*∆E16 mice were isolated by sorting. ChIP assays were performed using the True MicroChIP-seq Kit (DIAGENODE Cat# C01010132) according to the manufacturer’s protocol. Briefly, samples were fixed with 1% formaldehyde for 10 minutes. Chromatin was sheared using Bioruptor® Pico sonication device (DIAGENODE Cat# B01060010) in combination with the Bioruptor® Water cooler for 5 cycles, applying a 30 seconds [ON] 30 seconds [OFF] setting. The shearing efficiency of IP DNA was analyzed by PCR, and fragments of 300–500 bp were visualized on a 2% agarose gel.

Antibodies for histone marks (H3K4me3, True MicroChIP-seq Kit and H3K27me3, Abcam (ab195477)) were used. Equal amounts of 0.5 μg each of the rabbit IgG negative control and the H3K4me3 positive control antibody were added. For ChIP sequencing, libraries were prepared by the Genomics Unit of the Scientific Park Madrid and sequenced using NextSeq 2000 High Output Run Mode V4 (Illumina) as single-end 100-bp reads.

### Computational analysis

Raw fastq reads were aligned to the mouse reference genome (GRCm38/mm10 assembly, http://genome.ucsc.edu) using Bowtie2 (bowtie-bio.sourceforge.net/bowtie2) and Burrows-Wheeler aligner BWA-MEM 0.7.15 (github.com/lh3/bwa.git) with standard settings. The aligned reads were then converted to BAM files using Picard tools 2.9.0 (http://broadinstitute.github.io/picard). Duplicate reads were removed using Picard tools. BED files were imported into RStudio and annotated with the R/Bioconductor package ChIPseeker (github.com/YuLab-SMU/ChIPseeker) [54]. The promoter region was defined as − 1 kb to + 200 bp from the transcription start site (TSS). Additionally, we utilized the Bioconductor packages org.Mm.eg.db and TxDb.Mmusculus.UCSC.mm10.knownGene for peak annotations. Gene ontology (GO), pathway annotation, and enrichment analyses were conducted using GSEA (gsea-msigdb.org) with the Molecular Signatures Database (MSigDB) (go-basic.obo, downloaded Jan 15, 2020). The significance of overlap between data sets was assessed using the enrichPeakOverlap function in ChIPseeker, with the number of random permutations (nShuffle) set to 10,000. To evaluate read coverage distribution across the genome, bigWig files (10-bp genomic bins) were generated using bamCoverage/deepTools 2.3.1 [41] and normalized to account for differences. The aligned sequence reads, coverage, and ChIP-seq and ATAC-seq peaks were visualized using IGV (https://igv.org; [42]. Multi-omics analysis was performed with Metascape (metascape.org) [57].

*RNA-seq data analysis* Sequenced reads were processed in parallel using the Galaxy RNA-seq pipeline (http://usegalaxy.org) and a combined approach with BWA-MEM, StringTie (ccb.jhu.edu/software/stringtie/) and edgeR (bioconductor.org/packages/edgeR). This combined approach was previously applied to process RNA-seq data of *Dido1*∆E16 in embryonic stem cells (GEO: GSE152346). Differentially expressed genes (DEGs) were defined based on an absolute log2 (fold change) ≥0.6 and FDR <0.05, unless stated otherwise. A Venn diagram was created to identify co-expressed DEGs among samples using the webtool at http://bioinformatics.psb.ugent.be/webtools/Venn/, provided by Bioinformatics & Evolutionary Genomics (Gent, BE). The biological significance of DEGs was explored using STRING v.11.0 (string-db.org).

*ATAC-seq data analysis* The model-based analysis of ChIP-seq (MACS; github.com/taoliu/MACS/) (Yong [55]) version 2.1.2 and HOMER 4.11[24] were employed to identify accessible chromatin regions between WT and *Dido1*ΔE16 LSK cells. The overlap between accessible regions identified by MACS and HOMER was found to be 80–90% (see Supplementary Table 4). To determine equivalent accessible regions in the replicas, we used the Intersect function of BedTools [40] along with the BED files produced by MACS. Sequences overlapping with ENCODE blacklist regions (mm10-blacklist.bed.gz) were excluded using BedTools before calculating accessible chromatin regions. Significant changes in chromatin accessibility between equivalent regions were assessed using DESeq2 and edgeR, defining significant changes as those with a fold change >2 and a false discovery rate (FDR) <0.05. Genes, promoters, UTRs, introns, and exons were assigned to the accessible regions using ChIPseeker.

*ChIP-seq data analysis* BAM files were processed with MACS (github.com/taoliu/MACS/) [55] version 3.0.0b3 for enrichment scoring and peak calling. Peaks were called using the callpeak function in MACS with an extension size of 147 bp. Differential binding between experimental conditions was analyzed using the bdgdiff function in MACS, with a gap distance of 73 bp and a minimum region length of 147 bp. Read count normalization was performed on alignment files to adjust for differences in sequencing depth, followed by background correction based on input using the bdgdiff function of MACS. Peaks overlapping with ENCODE blacklist regions (mm10-blacklist.bed.gz) were removed from downstream analyses using BedTools [40].

### Statistical analysis

Results are presented as mean ± standard error of the mean (SEM). Statistical significance was assessed by one-way analysis of variance (ANOVA) or the Student’s *t*-test, conducted with GraphPad Prism version 6 software. A p-value of <0.05 was considered statistically significant.

## Results

### Loss of the Dido3 protein results in anemia and severe peripheral lymphopenia

To investigate the *in vivo* the role of Dido3 in hematopoiesis, we deleted E16 of *Dido1* in murine HSC during early development. Mice homozygous for the floxed allele (*Dido1*flE16/flE16) were crossed with mice carrying one functional and one mutated allele of *Dido1* (*Dido1*∆E16, which lacks the C terminus of Dido3) along with the *Vav1:iCre* transgene, with an additional Cre-inducible *R26R*-*EYFP* allele. The resulting *Dido1*flE16/∆E16;R26R-EYFP;iCre^Vav1^ mice (hereafter referred to as *Dido1*∆E16) served as experimental subjects, while their *Dido1*flE16/+;R26R-;EYFP;iCre^Vav1^ littermates (hereafter referred to as WT) were used as controls, unless otherwise specified.

The ablation of *Dido1* E16 during early hematopoietic development was confirmed by qPCR on DNA samples from sorted bone marrow LSK cells, revealing a >99% reduction in *Dido1*∆E16 DNA content (Supplementary Fig. 1B). A corresponding reduction in Dido3 expression occurs at a later stage (prepro-B cells) as shown by RT-qPCR analysis, without affecting the expression of the rest of the locus (Supplementary Fig. 1C). Hematological analyses indicated a significant decrease in cellular components when comparing *Dido1*∆E16 mice to heterozygous littermate controls. Specifically, leukocyte numbers were reduced to 33% and erythrocyte numbers decreased to 92% of their respective values. Within the leukocyte fraction, the lymphocyte subpopulation was primarily responsible for the observed defect, showing a reduction to 27% (Supplementary Fig. 1D). Flow cytometry analysis of peripheral blood samples from WT and *Dido1*∆E16 mice for EYFP reporter expression revealed that >97% of circulating cells were EYFP-positive in both lymphoid and myeloid gates (Supplementary Fig. 1D, E).

As assessed by complete blood count and flow cytometry data, *Dido1*∆E16 mice exhibited markedly decreased counts of lymphocyte subsets, with the most significant reduction in B220 (95% decrease), followed by CD8 (75%), CD4 (60%) and NK1.1 (27%). The number of monocytes and granulocytes remained unchanged (Supplementary Fig. 1D). Furthermore, no differences were observed in the percentages of the various leukocyte populations between homozygous *Dido1* WT/WT mice and heterozygous WT/*Dido1*∆E16 mice (data not shown).

### Loss of Dido 3 results in reduced total bone marrow cellularity

**Fig. 1:**
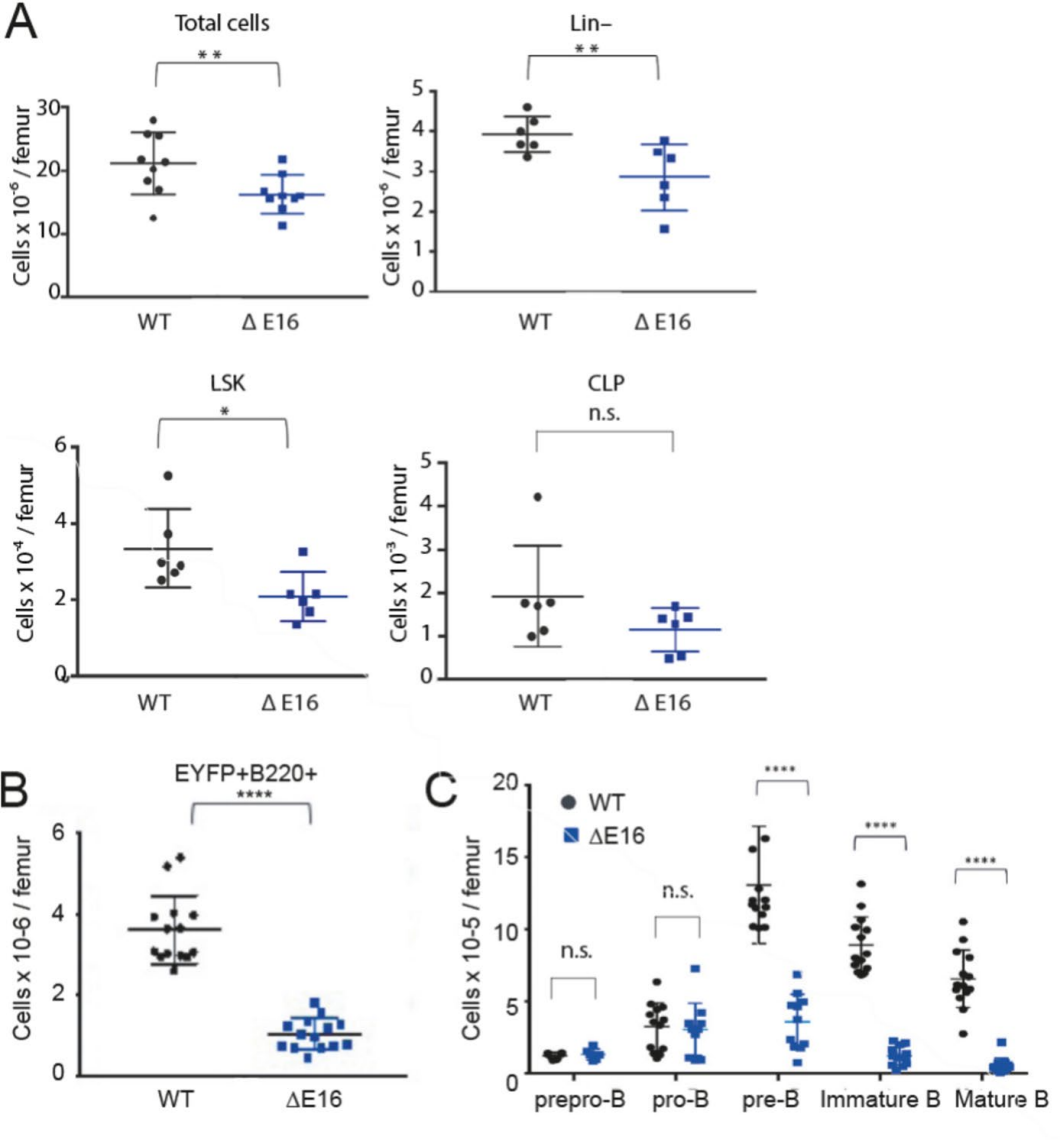
Altered hematopoietic cell populations in *Dido1*∆E16 mice indicate impaired B cell development compared to WT. **A**. Quantification of total (WT n = 9, *Dido1*∆E16 n = 9), lin⁻, LSK and CLP cells (WT n = 6, *Dido1*∆E16 n = 6) per single femur (t-test ** p < 0.01, * p < 0.05). Bars, mean ± SD. **B**. Numbers of EYFP^+^B220.^+^ cells per femur in WT compared to *Dido1*∆E16 mice (n = 14; **** p < 0.0001). **C**. Total bone marrow B precursors and mature recirculating B cells isolated from a single femur. Prepro-B (n = 6); pro-B, pre-B (WT n = 13, *Dido1*∆E16 n = 11); immature and mature B cells (WT n = 14, *Dido1*∆E16 n = 13). **B, C.** Means ± SD are shown, t-test **** p < 0.0001

Since *Dido1* has been previously linked most closely to stem cell developmental and differentiation functions, we analyzed the B-cell lineage in the bone marrow. We used flow cytometry to identify LSK and common lymphoid progenitor lin^−^Sca1^+^cKit^+^IL7R^+^ (CLP) cell populations (Supplementary Fig 2A, B), and found no significant differences in their relative proportions. However, *Dido1*∆E16 mice showed reduced total bone marrow cellularity and significantly lower absolute numbers of LSK cells (Fig. 1A).

We analyzed the distribution of different subpopulations of B-cell precursors, specifically prepro-B (EYFP^+^B220^+^CD19^-^CD93^+^IgM^-^), pro-B (EYFP^+^B220^+^CD19^+^CD43^+^IgM^-^), pre-B (EYFP^+^B220^+^CD19^+^CD43^-^IgM^-^) and immature B (EYFP^+^B220^+^CD19^+^IgM^low^) cells (Supplementary Fig. 2C). The number of B220^+^ cells was significantly lower in *Dido1*∆E16 mice compared to controls (Fig. 1B). The most affected populations were pre-B and immature B cells, while the numbers of prepro-B and pro-B cell were similar in both genotypes (Fig. 1C).

### Dido3 Deficiency increases apoptosis and limits proliferation of immature B cells in bone marrow

Signaling from the pre-BCR complex promotes the survival and proliferation of cells that have successfully rearranged the *Igh* locus. This serves as the first checkpoint, where non-functional heavy chain rearrangements lead to cell death by apoptosis.

**Fig. 2:**
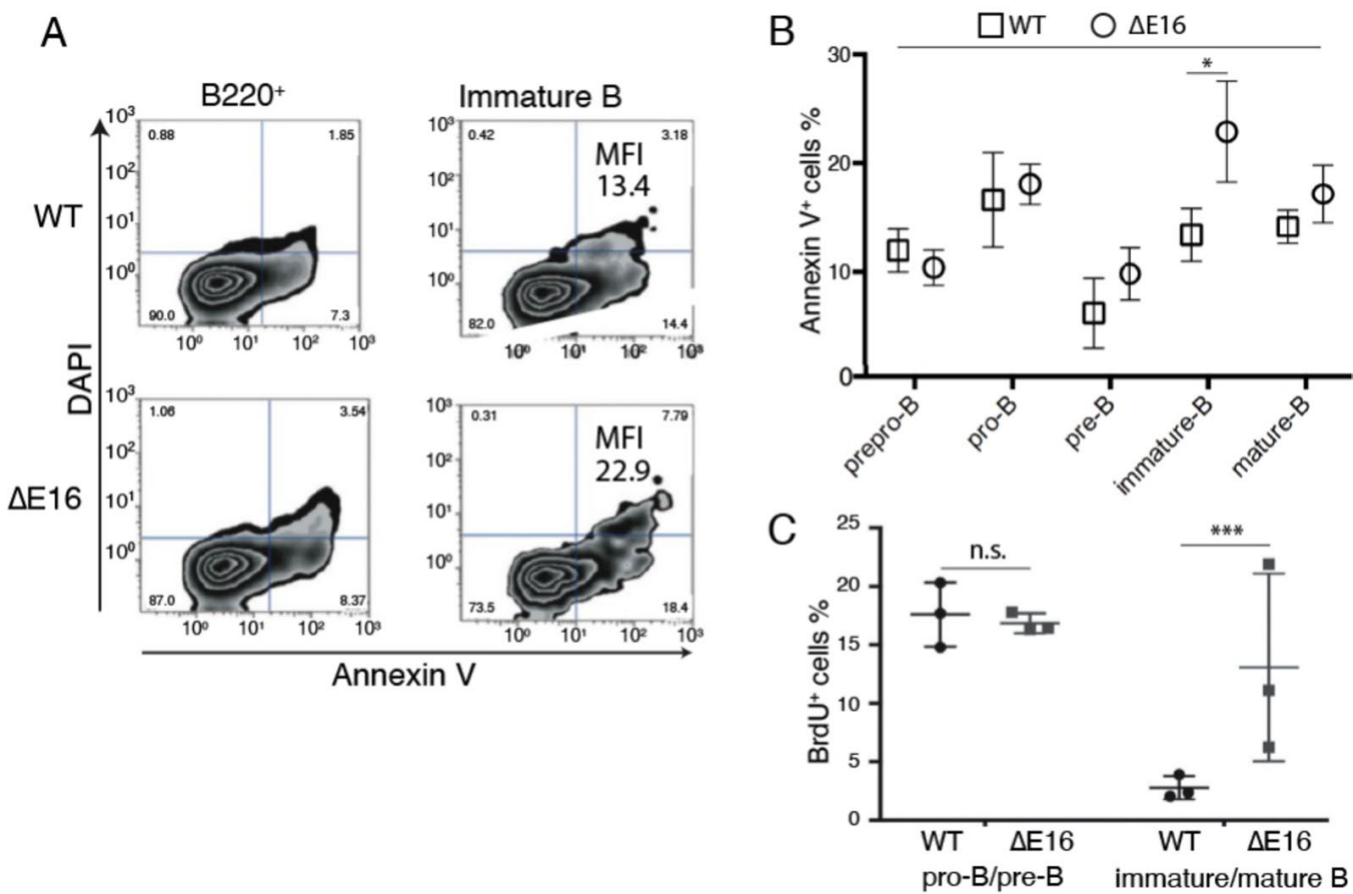
Increased apoptosis and altered proliferation in *Dido1*∆E16 B cell populations compared to WT. **A**. Representative graphs showing annexin V binding total B220^+ ^(left) and immature B cells (right) from the bone marrow of WT and *Dido1*∆E16 mice. The mean fluorescence intensities (MFI) for immature cells were 13.41 (WT, top) and 22.9 (*Dido1*∆E16, bottom). The lower left quadrant shows viable cells; the lower right, cells in early apoptosis (which bind annexin V and exclude DAPI); the upper right, cells in late apoptosis (which bind annexin V and incorporate DAPI); and the upper left, necrotic cells. **B**. Mean percentages of annexin V + cells in different precursor subpopulations (mean ± SD, n = 3, t-test * p < 0.05). **C**. Percentages (mean ± SD; n = 3) of cells incorporating BrdU in pro-B/pre-B and immature/mature B cell populations, from the bone marrow of WT and *Dido1*∆E16 mice, assessed 18 h after intraperitoneal BrdU injection

In a second checkpoint, immature B cells whose BCR is activated by an autoantigen in the bone marrow can either undergo receptor editing to modify their light chain or be deleted through apoptosis [37]. To detect cell death in distinct precursor populations, we utilized flow cytometry on bone marrow samples from *Dido1*∆E16 and WT mice, labeling them with appropriate antibodies, annexin V, and DAPI. Apoptosis levels were slightly higher, though not statistically significant, in *Dido1*∆E16 mice. In the immature B cell population, both the number of apoptotic cells and the annexin V mean fluorescence intensity (MFI) in bone marrow of *Dido1*∆E16 mice showed a significant increase compared to WT (Fig. 2A and B). This suggests that the size of the immature B cell population in *Dido1*∆E16 mice is limited by an increase in apoptotic processes at this stage. Our data support the notion that Dido3 is essential for efficiently overcoming the BCR signaling-dependent checkpoint.

We employed BrdU (5-bromo-2'-deoxyuridine) incorporation *in vivo* to identify and examine proliferating cells, using DAPI to determine DNA content and cell cycle stages. The percentage of cells that incorporated BrdU after 18 h post-intraperitoneal injection was quantified for pro-B/pre-B and immature/mature B cells. Cells from *Dido1*∆E16 mice in pro-B/pre-B states showed BrdU incorporation similar to that of WT mice (Fig. 2C). In the immature cell population, there was a non-significant increase in both the percentage and MFI of cells that incorporated BrdU (Fig. 2D). The percentage of S phase cells of the pro-B/pre-B populations was similar between both mouse groups, although an increase in the percentage of *Dido1*∆E16 mature/immature B cells was observed in this phase (Fig. 2D). The increase in the percentage of immature B cells in the S phase in *Dido1*∆E16 compared to control mice, along with the rise in apoptotic cells suggests that immature B cells from *Dido1*∆E16 mice experience an S phase block that eventually leads to apoptosis.

*In vitro* differentiation assays were carried out to determine whether the B cell precursors from *Dido1*∆E16 mouse exhibited alterations in their capacity to differentiate toward the B lineage. We purified cells lacking lineage-specific markers (lin^-^) trough negative selection and plated them in a semi-solid matrix and medium optimized for B cell development, supplemented with IL7. In cultures from WT mice, between 25 and 35 colonies were counted in plates seeded with 2 x 10^6^ lin^–^ cells/ml, along with 5 to 10 smaller cell groups per plate. No colonies were found in plates seeded with lin^-^ precursors from *Dido1*∆E16 mice; however, we did detect some clusters consisting of a small number of disaggregated cells. Thus, B-lineage (B220^+^) cells from conditional mutant mice displayed differentiation defects when stimulated *in vitro*, resulting in blocked proliferation and maturation of B-cell precursors under these conditions.

### Identification of essential regulators of stem cell function and the B cell pathway by ATAC-seq analysis of bone marrow LSK cells

Since DIDO1 has been shown to be a chromatin interactor that recognizes accessible chromatin regions with H3K4me3 histone marks [49], we analyzed this LSK population using Omni-ATAC-seq [12]. Two biological replicates with accessible regions were used for differential analysis between WT and *Dido1*∆E16 LSK cells.

**Fig. 3:**
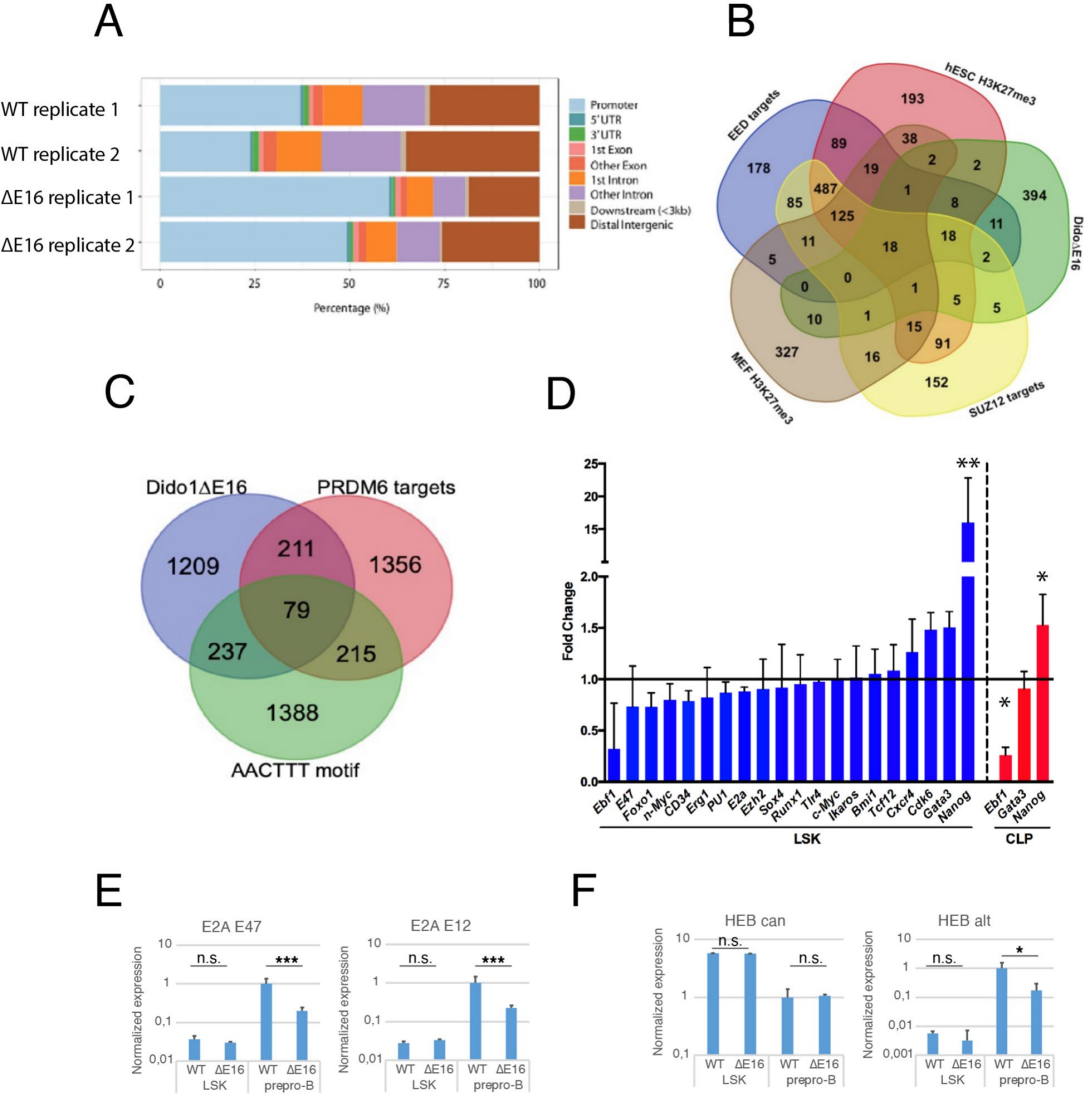
Differential chromatin accessibility and gene expression changes in *Dido1*∆E16 LSK cells. **A**. Distribution relative to transcription start sites of chromatin open in WT and closed in *Dido1*∆E16 LSK cells, as determined by ATAC-seq (mean values, n = 3). **B**. Venn diagrams illustrating the overlap in promoter regions of differentially accessible chromatin with genesets associated with PRC2 activity. **C**. Overlap of differentially accessible chromatin in intronic and intergenic regions. **D**. Validation of the expression of potentially affected genes using real-time qPCR (n = 4, t-test ** p < 0.01, * p < 0.05). **E**. Differential expression of *Tcf3* and its two isoforms in prepro-B cells and in lin⁻ precursors, with values relative to WT prepro-B levels. **F**. Expression levels of various *Tcf12* isoforms in *Dido1∆*E16 prepro-B cells, each compared to the expression of the same region in WT cells (n = 4, t-test *** p < 0.001, * p < 0.05)

Accessible regions were annotated with the ChIPseeker program [54], associating the position of the accessible regions in each replicate with the structure of the genes (promoter, 5’-UTR, 3’-UTR, exon, intron, or intergenic) in the mouse genome mm10. The largest percentage of accessible regions was concentrated in gene promoters, introns, and intergenic regions in both WT and *Dido1*∆E16 LSK cells (Fig. 3A). However, in the *Dido1*∆E16 samples, there was a relative reduction in the number of introns and intergenic regions compared to promoters, which was related to the preferential association of DIDO1 with enhancer sequences over promoters [13].

A Gene Set Enrichment Analysis (GSEA) of the ATAC-seq data was performed. GSEA is a computational method that determines whether *a priori* defined sets of genes show concordant and significant differences between two biological states [48]. In seeking overlaps with “curated gene sets” (C2 collection) of MSigDB database, we found that the promoters with the least accessibility in *Dido1*∆E16 LSK cells coincided with (1) genes with mixed epigenetic marks (H3K4me2 and H3K27me3) in mouse embryonic fibroblast (MEF) HCP genes (highCpG-density promoters), p=2.66 x 10^−57^; (2) silencing marks (H3K27me3) in embryonic cells, p=1.99 x 10^−38^; (3) targets of human EED, a core subunit of Polycomb Repressor Complex 2 (PRC2), p=1.61x 10^−33^, and (4) human SUZ12 targets (also a core subunit of PRC2), p=5.56 x 10^−31^ (Supplementary Table 1). In accordance with these data, our previous microarray dataset (GEO: GSE85029, [16]) analyzed here by GSEA, indicates that genes silenced in Dido3-deficient embryonic stem cells (ESC) correlate significantly with those silenced in mouse SUZ12-deficient ESC,the genes overexpressed in these cells also coincided with those overexpressed in the Suz12^–/–^ cells. Thus, there appears to be a relationship between the epigenetic regulation of gene expression by PRC2 and the presence of Dido3. We also constructed a Venn diagram representing DEGs associated with distinct gene sets from the MSigDB database (Fig. 3B). Additionally, there was a correlation with the geneset of genes expressed after p53 activation (p<1.48 x 10^−24^), consistent with the *Cdkn2a* alterations observed in *Dido1*∆E16 mouse samples at later developmental stages (see below). When the analysis was limited to non-promoter regions (intra- or intergenic), using the MSigDB “regulatory target gene sets” C3 collection, there was significant overlap with the targets of the putative histone-lysine N-methyltransferase PRDM6 (p<7.5 x 10^−24^), and the set of genes with at least one occurrence of the highly conserved motif M17 AACTTT, a potential binding site for an unknown transcription factor in the region spanning up to 4 kb around their transcription start sites (p<5.91 x 10^−69^) (Fig. 3C). Among the 79 genes common to the three gene sets, we identified several essential regulators for stem cell function and B cell pathway entry, including *Akt3, Runx1, Ebf1, Igfr1, Tcf4, Cdk6*, and *Etv6*. GSEA analysis of the few identified sites that showed higher accessibility in *Dido1*∆E16 LSK cells yielded no relevant results.

To validate the expression data in additional samples by RT-qPCR, we selected several genes from the list of regions with different Tn5 accessibility in WT and *Dido1*∆E16 LSK cells. These included genes related to the Polycomb group (PcG) proteins (*Bmi1, Ezh2*), maintenance and differentiation of stem cells (*Runx1, Nanog, Ikzf2* (Helios), *Tcf12* (Heb)), differentiation towards lineage B (*Klf3, Il7r, Ebf1, Tcf3* (E2A, E12/E47), *Spi1* (PU.1), *Foxo1*) or towards T lineage (*Gata3*), and cell proliferation (*Mycn* (N-myc), *Myc, Cdk6*). The analysis showed that the expression of genes related to differentiation towards the B lineage was non-significantly lower in *Dido1*∆E16 than in WT LSK cells. Conversely, genes such as *Gata3* and *Cdk6* were significantly overexpressed in *Dido1*∆E16 LSK cells, with *Nanog* levels being 16 times higher than in WT LSK cells (Fig. 3D). Dido*1*∆E16 CLP samples exhibited significantly reduced *Ebf1* expression, whereas *Nanog* levels remained normal. As in nearly every developmental process, the three “E-proteins” play a fundamental role in hematopoiesis [6]. These transcription factors are characterized by a basic helix-loop-helix (bHLH) domain, allowing them to homo- and hetero-dimerize with themselves and other HLH proteins, and to bind to DNA through E-boxes (CACGTG sequences). *Tcf3* (which encodes E2A isoforms E12 and E47) and *Tcf12* (which encodes HEB isoforms HEBcan and HEBalt) have been implicated in B cell development [3]. To test the expression of the distinct isoforms, we designed specific primers for conventional RT-qPCR assays. *Tcf3* expression was lower in WT and *Dido1*∆E16 LSK cells than in prepro-B cells; this reduction was attributed equally to E12 and E47 isoforms (Fig. 3E). However, while the expression of the HEBcan isoform of Tcf12 (assessed by exon 1/2 expression levels) was unaffected by Dido3 absence, the expression of the HEBalt isoform (assessed as exon 1/2 of X9 mRNA isoform levels) dropped to 10% of the WT levels in *Dido1*∆E16 prepro-B cells (Fig. 3F).

### Dido3 deficiency alters gene expression in PRC2 targets and impairs immunoglobulin locus transcription in pre-B cells

The first checkpoint in B cell development depends on pre-BCR activation and occurs at the pre-B stage [11] and we observed the most significant effects of Dido3 absence at this stage, specifically by a lack of progenitor cell progression. Therefore, we sequenced the transcriptome of WT and *Dido1*∆E16 cells during this phase.

**Fig. 4:**
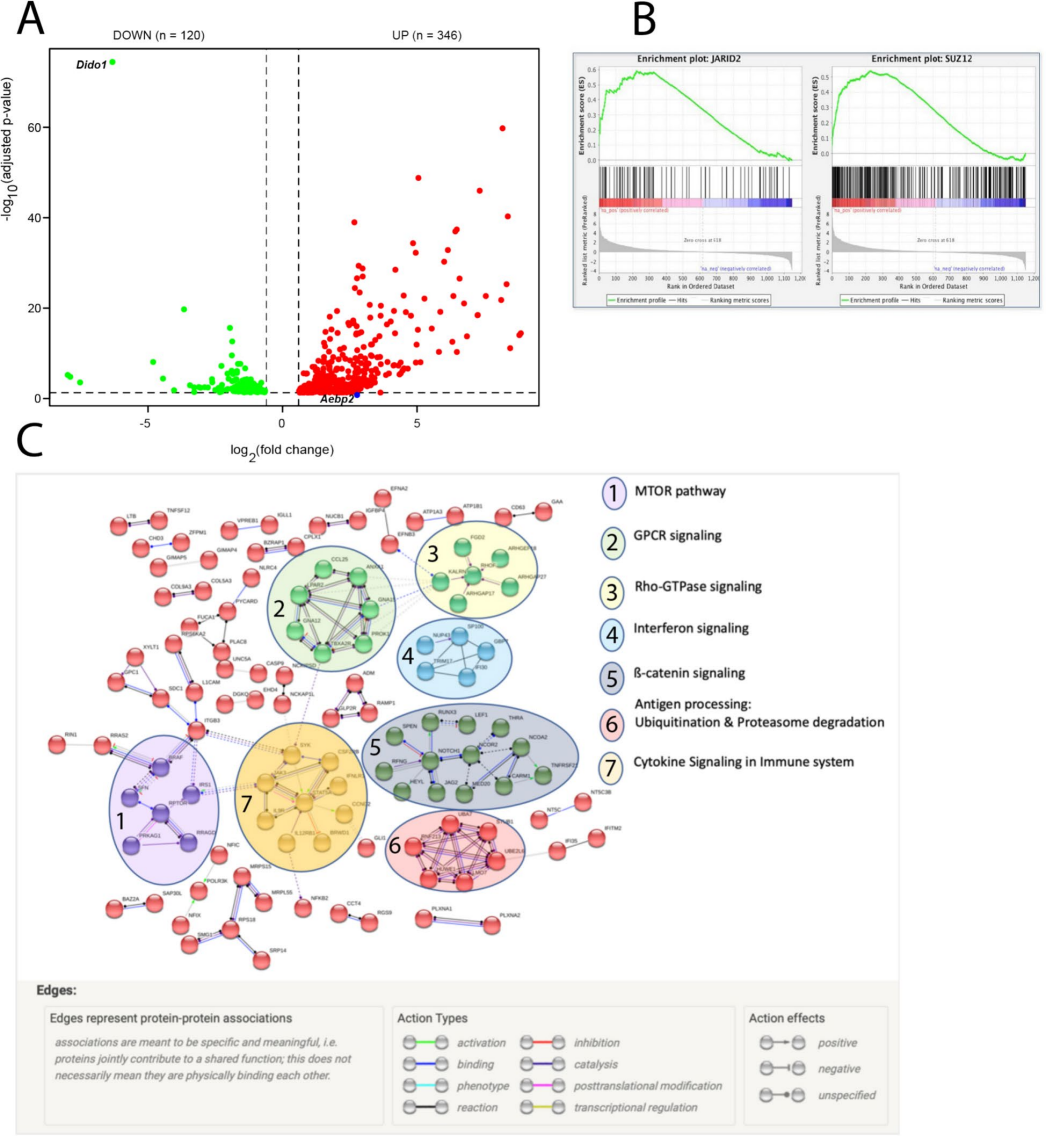
RNA-seq reveals the involvement of key genes for B-cell development. **A**. Dot plot representation of the RNA‐seq results. The red dots represent significantly upregulated genes in *Dido1*∆E16 pre-B cells, the green dots represent significantly downregulated genes (|log2 FC|≥ 1 and FDR < 0.01). **B**. GSEA enrichment graphs of genesets relative to PRC2 activity and differentially expressed genes in pre-B cells. The RNA-seq expression data are ranked according to fold change in the transcription levels in pre-B *Dido1*∆E16 cells, compared to pre-B WT cells. A maximal correlation was observed between the expression of altered genes in pre-B *Dido1*∆E16 cells and genes identified by ChIPseq as targets of PRC2 components JARID2 (p < 0.001; FDR = 0.213) and SUZ12 (p < 0.001: FDR = 0.217). **C**. STRING analysis and clustering of differentially expressed genes between *Dido1*∆16 and WT LSK cells. It shows that differentially expressed genes are involved in known and predicted protein–protein interactions. Interaction score > 0.4

Using pooled samples (2–3 mice/pool), we purified RNA from flow cytometry-sorted pre-B cells. Two biological replicates were used to construct libraries for high throughput sequencing. We identified 120 genes with higher expression in WT and 346 genes upregulated in *Dido1*∆E16 pre-B cells (Fig. 4A and Supplementary Table 2).

For a first analysis, we applied the GSEA tool, using a ranked list of genes with baseMean greater than 1, ordered by fold change. Analyzing against MSigDB M3 subcollection (GTRD, mouse transcription factor targets, https://www.gsea-msigdb.org/gsea/msigdb), we identified downregulated gene sets comprising targets of the PRC2 components JARID2 and SUZ12 (p<0.001) (Fig. 4B). Additionally, *Aebp2*, a substoichometric PRC2 subunit that enhances K9 and K27 methylation of histone H3 [21], was found to be upregulated (log_2_FC = 2.61 FDR = 0.067) in *Dido1*∆E16 pre-B cells, although below the threshold of significance.

Then, we utilized protein-protein interactions from the STRING database v.11.0 to explore the biological significance and functional associations of differentially expressed genes. Gene Ontology (GO) terms with a false discovery rate (FDR) of less than 0.25 included positive regulation of leukocyte differentiation, cellular response to stimuli, positive regulation of lymphocyte activation, positive regulation of lymphocyte differentiation, positive regulation of T cell differentiation, positive regulation of B-cell differentiation, signal transduction, regulation of signaling, immune response, and regulation of leukocyte cell-cell adhesion.

Following gene clustering, the key signaling pathways identified were MTOR, GPCR, Rho-GTPase, interferon, ß-catenin, cytokine signaling in the immune system, and antigen processing by ubiquitination and proteosome degradation (Fig. 4C).

**Fig. 5:**
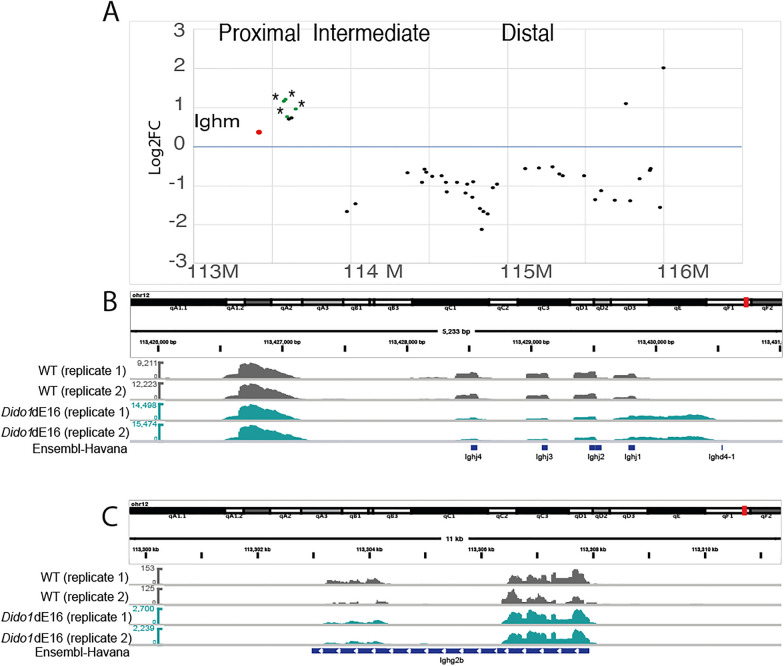
Analysis of the *Igh* locus in *Dido1*∆E16 pre-B cells reveals defects in gene rearrangement. **A**. Relative abundance of *Ighv* regions in the transcriptome of *Dido1*∆E16 pre-B cells, presented as log fold change (logFC) compared to WT levels (green dots represent significantly overexpressed regions, *, p < 0.05). The bottom section shows chromosome coordinates, while the top indicates the main regions (proximal, intermediate, distal) where distinct V families are mapped. **B**. Integrative Genomics Viewer (IGV; http://www.broadinstitute.org) plot displaying RNA-seq data that illustrates transcription from the DNA region between D and J regions; positions of J fragments and the more distal D fragment (D4) are represented. **C**. IGV plot with RNA-seq data showing transcription of the *Igg2b* gene in *Dido1*∆E16 pre-B samples

The relevance of pre-BCR in the pre-B developmental checkpoint, along with the involvement of PRC2 in V(D)J recombination [47], prompted us to investigate the impact of Dido3 deficiency on the transcription of the *Igh* and *Igk* loci. The joining (J) and variable (V) regions of the *Igk* locus were not significantly affected. However, we observed an over-representation of proximal *Igh* V fragments (specifically the DQ52 and 7183 families), while intermediate and distal V fragments (mainly V_H_J558 and 3609 families) were under-represented in *Dido1*∆E16 pre-B cells (Fig. 5A and Supplementary Table 3).

Unexpectedly, we detected high transcription levels of DNA located between J and D fragments (Fig. 5B). In WT cells, this DNA segment is typically eliminated through recombination during the prior pro-B stage [26], or even in prepro-B stage, by RAG1/2 activity. Finally, we observed expression of the *Igg2b* (gamma-2b) gene, indicating that isotype-switched IgG2b^+^ memory B cells co-purified with pre-B cells in significantly higher numbers compared to WT samples (Fig. 5C).

### Chromatin immunoprecipitation sequencing (ChIP-seq) analysis reveals the role of Dido 3 genes with H3K27me3

**Fig. 6:**
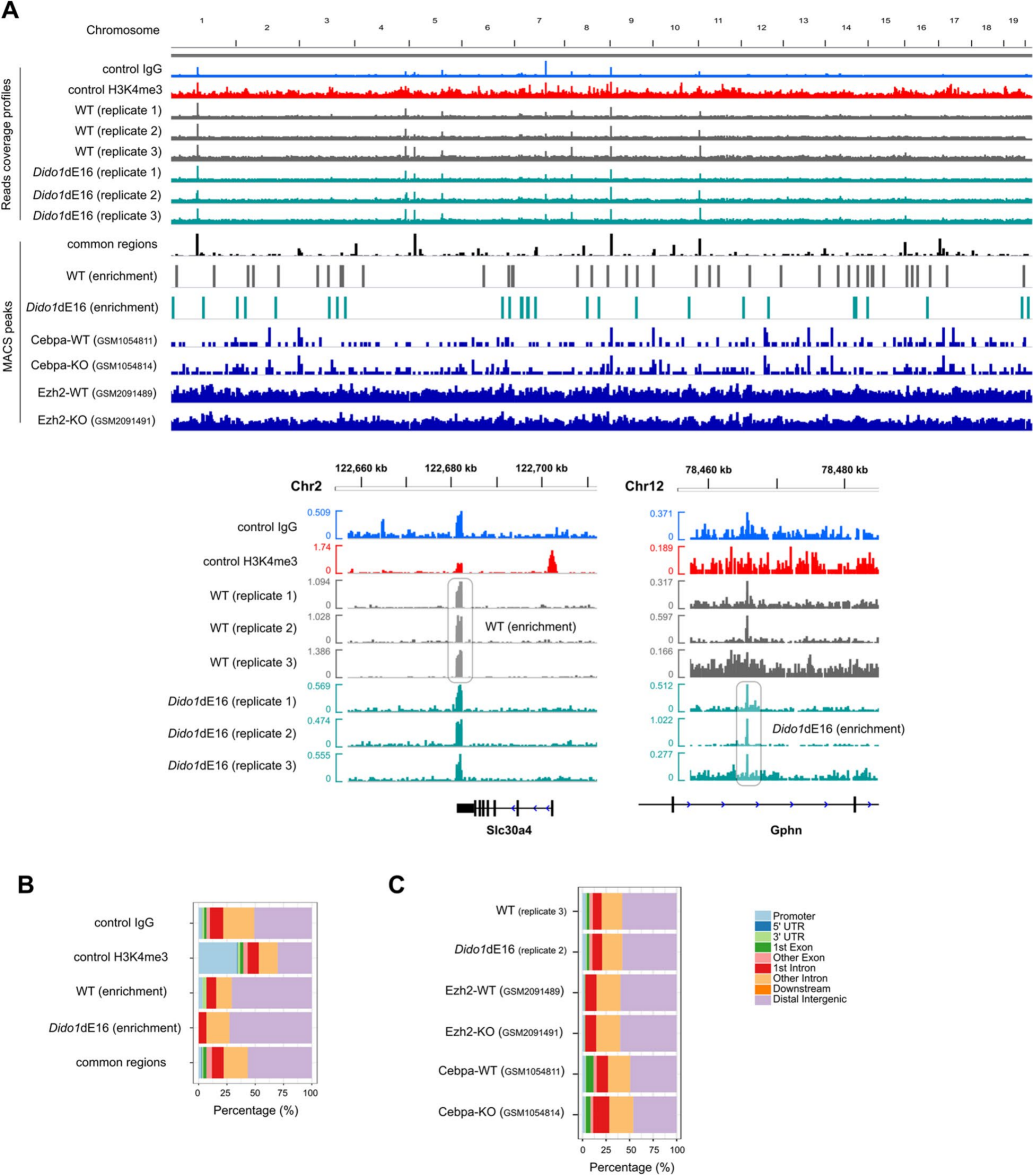
H3K27me3 profile in WT and *Dido1*ΔE16 LSK cells is compatible with ChIP-Atlas validated datasets. **A** Genome browser snapshot of genomic regions enriched for H3K27me3 chromatin marks. The top eight tracks were obtained from female (n = 4) and male (n = 4) mice samples with matched ages. The distribution of a non-specific control IgG and specific H3K4me3 peaks in WT LSK cells is also shown. Seven MACS peaks data tracks are provided: one for H3K27me3 signal in common regions between WT and *Dido1*ΔE16 LSK cells, one for enriched H3K27me3 signal in WT LSK cells, one for enriched H3K27me3 signal in *Dido1*ΔE16 LSK cells, and the last four (*Ezh2*-WT, *Ezh2*-KO, *Cebpa*-WT, *Cebpa*-KO) for ChIP-Atlas validated datasets of H3K27me3 signal for comparison (https://chip-atlas.org). Enriched H3K27me3 signal in WT, *Dido1*ΔE16, and common regions was calculated using the bdgdiff function in MACS. GEO accession codes are shown in parentheses. Insets at the bottom of the panel A shows ChIP-seq signals on two randomly selected genomic regions (Chr2:122,660,000-122,710,000 and Chr12:78,460,000-78,480,000). The Y-axis values indicate the mean of normalized reads per 10 bp (normalized using BPM with bamCoverage of deepTools). **B** and **C** Distribution of the enriched-peak distances to the nearest transcription start site as determined by ChIPseeker [54]

To investigate the effects of Dido3 on PRC2 regulation, we conducted H3K27me3 chromatin immunoprecipitation sequencing analysis on LSK cells isolated from WT and mutant *Dido1*ΔE16 mice (Fig. 6B, and Supplementary Tables 4 and 5). The majority of the H3K27me3 peaks were found in gene introns and intergenic regions in both WT and *Dido1*ΔE16 LSK cells (Supplementary Table 4). However, in WT cells, we observed a slightly higher number of genes with H3K27me3 enrichment compared to *Dido1*ΔE16 LSK cells.

Specifically, we identified 58 regions (48 genes) with significantly greater H3K27me3 enrichment in WT compared to *Dido1*ΔE16, and 44 regions (33 genes) that showed more H3K27me3 enrichment in *Dido1*ΔE16 compared to WT. Additionally, we found 726 regions (436 genes) with no significant H3K27me3 enrichment in LSK cells (Fig. 6A, Supplementary Table 5, and Supplementary File 1). The genes highlighted in Supplementary Table 5 are involved in various functions, including RNAPII transcription, RNA processing, histone H4 binding and modification, cell identity and differentiation, immune response in B-cells, and the maintenance of hematopoietic stem cells/hematopoiesis. Notably, some of these genes were differentially expressed in pre-B cells (see below).

We also compared the H3K27me3 ChIP-seq data from WT and *Dido1*ΔE16 with other GEO datasets reported in two different studies. Specifically, we utilized datasets from Ezh2-KO (GSM2091489, GSM2091491) and Cebpa-KO LSK cells (GSM1054811, GSM1054814) (Supplementary Table 6). The analysis indicated a preference for H3K27me3 binding in gene introns and intergenic regions, with no significant differences observed between WT and mutant LSK cells (Fig. 6C and Supplementary Table 7). Furthermore, less than 10-12% of the H3K27me3 peaks in the mouse genome from both WT and *Dido1*ΔE16 LSK cells overlap with Ezh2-KO and Cebpa-KO datasets (Supplementary Table 8). This result may be influenced by the different experimental conditions employed (e.g., histone H3K27me3 antibody) across the three studies.

**Fig. 7:**
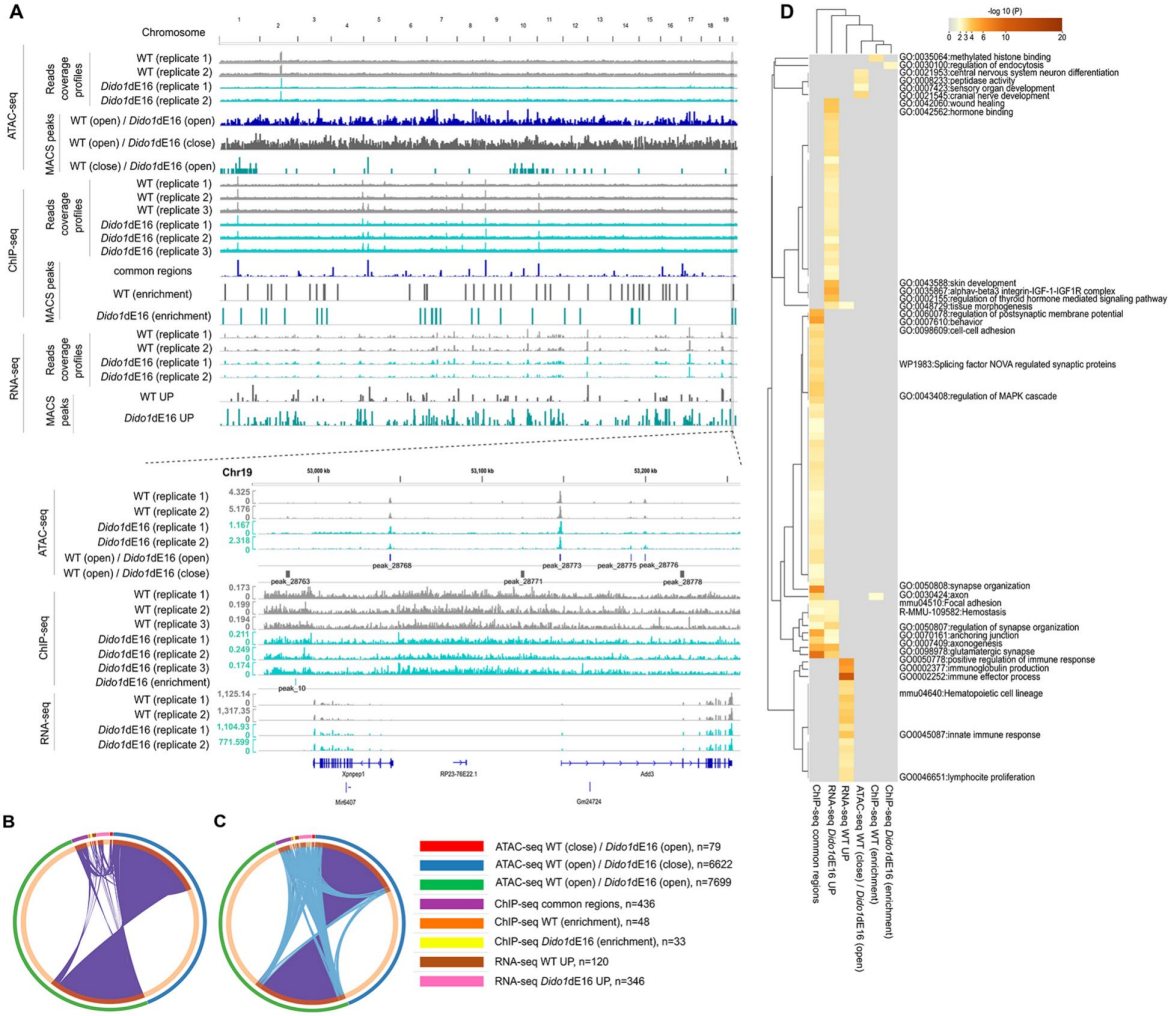
Integrative multi-omics analysis of chromatin accessibility, epigenomic, and transcriptomic data from WT and *Dido1*ΔE16 cells identifies distinct molecular alterations in B cell differentiation. **A**. Genome browser snapshot of genomic regions identified in ATAC-seq, ChIP-seq, and RNA-seq data analysis. The ATAC-seq and RNA-seq tracks were obtained from male (n = 8) mouse samples of different ages. The ChIP-seq tracks were obtained from eight samples as described in Fig. 6A. The bottom inset of panel A displays the ATAC-seq, ChIP-seq, and RNA-seq signals on a randomly chosen genomic region (Chr19:52,980,000–53,250,000). The peak identifiers are identical to those found in Supplementary Files 1 and 2. The Y-axis values indicate the mean of normalized reads per 10 bp (normalized using BPM with bamCoverage of deepTools). **B**. Overlaps between gene lists from ATAC-seq, ChIP-seq, and RNA-seq experiments. **C**. Overlaps between genes sharing the same enriched ontology terms (i.e., GO Biological Processes, Molecular Functions or Cellular Components, KEGG Pathway, Reactome Gene Sets, WikiPathways). On the outside, each arc represents the identity of each gene list, using the same color code as indicated in the legend. On the inside, each arc represents a gene list, where each gene member of that list is assigned a spot on the arc. Dark orange color represents the genes that are shared by multiple lists and light orange color represents genes that are unique to that gene list. Purple lines link the same gene that are shared by multiple gene lists (notice a gene that appears in two gene lists will be mapped once onto each gene list, therefore, the two positions are purple linked). Blue lines link the genes, although different, fall under the same ontology term (the term has to be statistically significantly enriched and with size no larger than 100). The greater the number of purple links and the longer the dark orange arcs implies greater overlap among the input gene lists. Blue links indicate the amount of functional overlap among the input gene lists. **D**. Hierarchical cluster of enriched ontology terms. The heatmap cells are colored by their p-values; gray cells indicate the lack of enrichment for that term in the corresponding gene list

The combined analysis of differentially expressed genes (RNA-seq), chromatin accessible regions (ATAC-seq), and H3K27me3 ChIP-seq data reveals that only a few genes were detected simultaneously across different experiments, although many share similar functions or belong to related pathways (Fig. 7). In meta-analysis, it is common to observe minimal direct overlap among studies due to variations in the biological assays employed (Fig. 7A). However, we noted greater functional overlap (Fig. 7B, C), as these studies likely capture different subsets of gene members involved in the same biological processes. Fig. 7D highlights the “immune effector process” (GO:0002252; logP = − 11.5) as the most enriched GO term among downregulated genes identified in the RNA-seq analysis.

In WT and *Dido1*ΔE16 LSK cells, approximately 26–28% of the H3K27me3 binding sites overlap with chromatin-accessible areas in *Dido1*ΔE16 LSK cells (Supplementary Table 9). The differential chromatin-accessible areas “WT(close)/MUT(open)” identified in ATAC-seq correspond to only 1% of the H3K27me3 marks (Supplementary Table 9). In contrast, as shown in Supplementary Table 10, 37% of H3K4me3 binding sites overlap with chromatin-accessible regions in WT cells, compared to 19% in *Dido1*ΔE16 LSK cells, as expected.

Large regions of repressive H3K27 methylation, alongside smaller regions of activating H3K4 methylation, constitute bivalent chromatin domains. In embryonic stem cells (ESC), these bivalent regions keep developmental genes inactive while ensuring their readiness for activation. PRC2 plays a critical role in the mechanism of bivalent gene silencing and activation by regulating chromatin accessibility [34]. Supplementary Table 11 provides a list of bivalent genes that are either upregulated or downregulated according to RNA-seq analysis.

## Discussion

Although Dido3 is expressed ubiquitously throughout the organism and presumably has a more general role in determining chromatin structure, its significant impact on the B cell population prompted us to focus our study on this cell lineage. Speculations regarding the molecular mechanism involved have been discussed in previous studies [17, 19, 20, 20, 36, 39, 45]. Here we demonstrate that Dido3 is involved in the progression of HSC into B cell differentiation.

In B lymphopoiesis, EBF1 is the key transcription factor for B cell specification and commitment [51]. Ectopic expression of *Ebf1* in HSC directs differentiation towards the B lineage at the expense of other lineages [56]. *Ebf1*^*–/–*^ mice exhibit a developmental blockade of the B lineage at the prepro-B stage and lack mature B cells [31]. Our mouse model shows a partial arrest in B cell development at the prepro-B and pro-B stages, similar to the phenotype observed in *Ebf1*^+/–^ mice. Our RT qPCR assays indicate that *Ebf1* expression in purified *Dido1*∆E16 CLP is approximately one-third of that seen in WT CLP cells, where the specification process towards the lymphoid lineage initiates. Heterozygous deletion of *Ebf1* leads to an impaired response to IL7 *in vitro* [1], which may explain the observed stage-specific reduction in cellular expansion. The loss of Dido3 in the LSK state activates *Nanog* transcription, which negatively modulates *Ebf1*. Consequently, reduced levels of EBF1 would diminish the potential of *Dido1*∆E16 CLP cells to differentiate into the B lineage.

All canonical E proteins are crucial for lymphoid development [28], HEB (*Tcf12*) and E2-2 (*Tcf4*) are believed to contribute to the overall E protein dosage required for B cell development, as the absence of either factor results in a decrease in pro-B cell numbers without causing a clear developmental block [58]. Among the E proteins, E2A is essential for maintaining the expression of *Ebf1*, *Foxo1*, and *Pax5*, thereby supporting the B cell genetic program. In Tcf3^+/–^; Tcf12^–/–^ mice, B cell development is completely halted at the CLP stage [53]. However, it remains unclear whether this arrest is due to the absence of HEBcan, HEBalt, or both isoforms. In our study, neither isoform is entirely absent,while HEBcan levels are normal, HEBalt levels in *Dido1*∆E16 prepro-B cells drop to 10% of those in WT, indicating a potentially uncharacterized function for this isoform. Lack of E2A function leads to an inability to rearrange *Igh* gene segments, a deficiency that can be rescued with a single dose of E2A [5]. Although it is yet to be determined if this effect is dose-dependent, it may account for our findings in *Dido1*∆E16 pre-B cells, where a significant number of RNA reads correspond to the segment between D and J fragments, which was efficiently deleted in WT samples.

We found significant association between the transcriptional alterations in *Dido1*∆E16 cells and several PRC2 targets, as well as genes marked by H3K27me3 epigenetic modifications. This suggests that Dido3 plays a role in regulating specific PRC2 activity within the B cell lineage. Additionally, the loss of EZH2 is known to impair distal VH–DJH rearrangement and *Igh* locus contraction [47], indicating a preference for using proximal V fragments in our model, which supports the idea that Dido3 coordinates with PRC2.

Previously, we have demonstrated differential expression of the DIDO isoforms in myeloproliferative disorders (MPD) [15]. Other studies have indicated that BCR-ABL1 translocation (Philadelphia chromosome,Ph) in chronic myelocytic leukemia (CML) may modulate *DIDO1* expression in a JAK2V617F-independent manner [9] [10]. Multiple signaling pathway disorders and genetic abnormalities associated with Ph are crucial for the development of various leukemia types. However, the reasons why MPD specifically evolve into CML, acute myeloid leukemia, acute leukemia leukocytosis, or mixed-phenotype acute leukemia remain unclear. BCR-ABL1 kinase hyperactivity activates signaling pathways and disrupts cellular processes [30], [46], with hyperactivation of these pathways confirmed in CML and acute lymphoblastic leukemia (ALL) mouse models. BCR-ABL1 primarily channels activity through the JAK/STAT and PI3K/AKT/mTOR pathways, as well as CCAAT/enhancer-binding proteins (C/EBP), which regulate normal myelopoiesis and myeloid disorders [27].

Our Gene Ontology analysis of RNA-seq data revealed that in cells lacking Dido3, the expression of components from PI3K/mTOR and JAK/STAT pathway, along with *Cebpb*, is transcriptionally affected. Furthermore, statistical analysis of DNA methylation indicated that target genes of NANOG [50], Polycomb-group target genes, and/or genes with an AACTTT promoter motif exhibit a methylation profile suggesting a predisposition to cancer development, such as pancreatic ductal adenocarcinoma [23]. This profile has also been linked to the promoter of *MEF2C* and is enriched at the promoters of genes involved in neurodevelopment [35]. Notably, this finding could correlate with the abnormal brain development observed in *Dido1* mutant mice [52].

## Conclusions

In summary, our data provide evidence of the critical involvement of Dido3, a reader of histone post-translational modifications (PTM), in several processes of bone marrow hematopoiesis, including cell cycle progression, apoptosis, and differentiation. Dido3 deficiency is associated with alterations in DNA accessibility and transcriptional defects in major B cell differentiation master regulators. This association seems to be related, at least in part, to the relationship of Dido3 with PRC2 and NANOG. The association between transcriptional alterations in *Dido1*∆E16 cells and PRC2 targets suggests that Dido3 may regulate PRC2 activity within the B cell lineage, influencing critical chromatin accessibility and gene expression patterns. This, together with its effect on the expression of NANOG suggests a potential predisposition to cancer development, highlighting the potential oncogenic implications of Dido3 dysregulation. Remaining open questions include the DNA methylation and the overall histone PTM landscape in Dido3-deficient cells, as well as the consequences of Dido3 deficiency in peripheral B cell differentiation, although our findings already support the multifaceted role of Dido3 in B cell development and its broader implications in immune system development and hematopoietic cancer biology.

## Supplementary Information

Additional file1 (PDF 523 KB)

Additional file2 (PDF 897 KB)

Additional file3 (PDF 160 KB)

Additional file4 (PDF 458 KB)

Additional file5 (PDF 236 KB)

Additional file6 (PDF 73 KB)

Additional file7 (PDF 99 KB)

Additional file8 (PDF 85 KB)

Additional file9 (PDF 69 KB)

Additional file10 (PDF 78 KB)

Additional file11 (PDF 83 KB)

Additional file12 (PDF 78 KB)

Additional file13 (PDF 102 KB)

Additional file14 (XLSX 111 KB)—ChIP-seq annotations

Additional file15 (XLSX 872 KB)—ATAC-seq annotations

## Data Availability

The datasets generated and analyzed during the current study are available from the corresponding author on reasonable request. Additional materials related to this study are also available upon request. ATAC-seq and RNA-seq data are deposited in the Gene Expression Omnibus database under accession code GSE157228, while ChIP-seq data can be accessed under accession code GSE272156.
