# Supplementary material for: The chromatin reader Dido3 is a regulator of the gene network that controls B cell differentiation
Source: Cell Biosci. 2025 Apr 26;15:56. doi: 10.1186/s13578-025-01394-x (PMC12034202; doi:10.1186/s13578-025-01394-x)
Supplement: Supplementary file 1 — Additional file1 (PDF 523 KB) [file 13578_2025_1394_MOESM1_ESM.pdf]

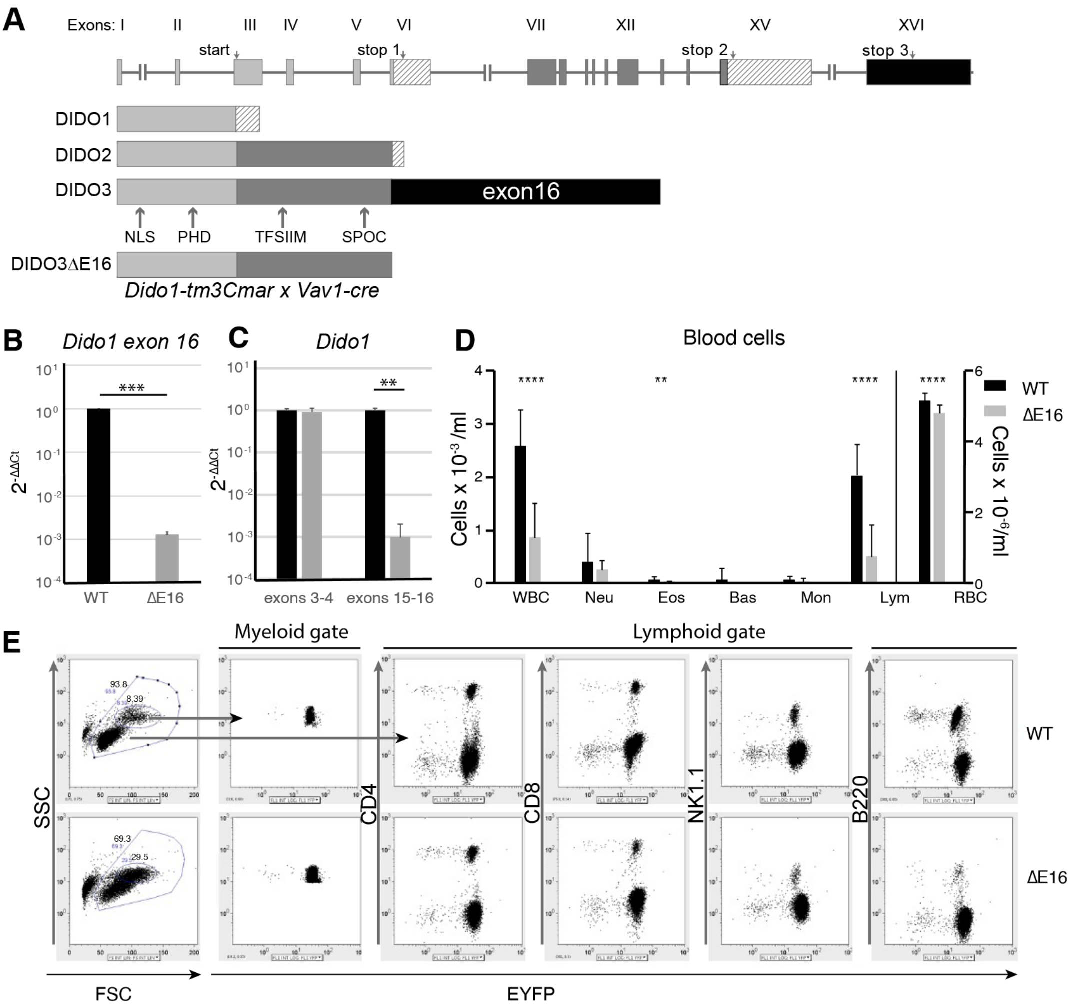

Supplementary figure 1. Deletion of Dido1 exon 16 in hematopoietic lineage. **A.** *Dido1* gene structure and Dido protein isoforms, and their domains in WT and conditional *Dido1*ΔE16 mutants; NLS nuclear location signal, PHD plant homeodomain, TFSIIM transcription elongation factor S-II subunit M, SPOC *Spen* paralog and ortholog module. **B.** Real-time qPCR of genomic DNA from bone marrow lin^{-} cells, normalized by ΔΔCt to *Ins* (internal control) and WT *Dido1* exon 16 (internal reference), n=4. **C.** Real-time TaqMan® qPCR of RNA from prepro-B cells, using exon-specific probes common to three DIDO isoforms (exons 2-3 boundary, probe Mm01215088_m1, Thermo Fisher Scientific Inc.) and specific for Dido3 (exons 15-16 boundary, probe Mm01215087_m1, Thermo Fisher Scientific Inc.), normalized by ΔΔCt with respect to *ß-actin* and *Gapdh* (internal controls) and WT *Dido1* (internal reference), n=3. **D.** Complete blood count in WT (n = 18) and Dido1ΔE16 (n = 8) mice. Each column shows the mean number of total leukocytes (WBC), neutrophils (Neu), eosinophils (Eos), basophils (Bas), monocytes (Mon), lymphocytes (Lym) and eritrocytes/reticulocytes (RBC). **E.** Flow cytometry analysis of peripheral blood WBC from WT (top row) and Dido1ΔE16 (bottom row) mice, showing cells that express EYFP as reporter of recombinase activity and *Dido1* exon 16 deletion. Left to right: Forward/Side Scatter (FSC/SSC) profile and the EYFP reporter expression in the myeloid gate and in CD4+, CD8+, NK1.1+ and B220+ cells. In **B, C** and **D,** t-test **** p <0.0001 ,*** p <0.001, ** p <0.01. Bars indicate the standard deviation (B, C) or the standard error of the mean (D).
