# Supplementary material for: The chromatin reader Dido3 is a regulator of the gene network that controls B cell differentiation
Source: Cell Biosci. 2025 Apr 26;15:56. doi: 10.1186/s13578-025-01394-x (PMC12034202; doi:10.1186/s13578-025-01394-x)
Supplement: Supplementary file 2 — Additional file2 (PDF 897 KB) [file 13578_2025_1394_MOESM2_ESM.pdf]

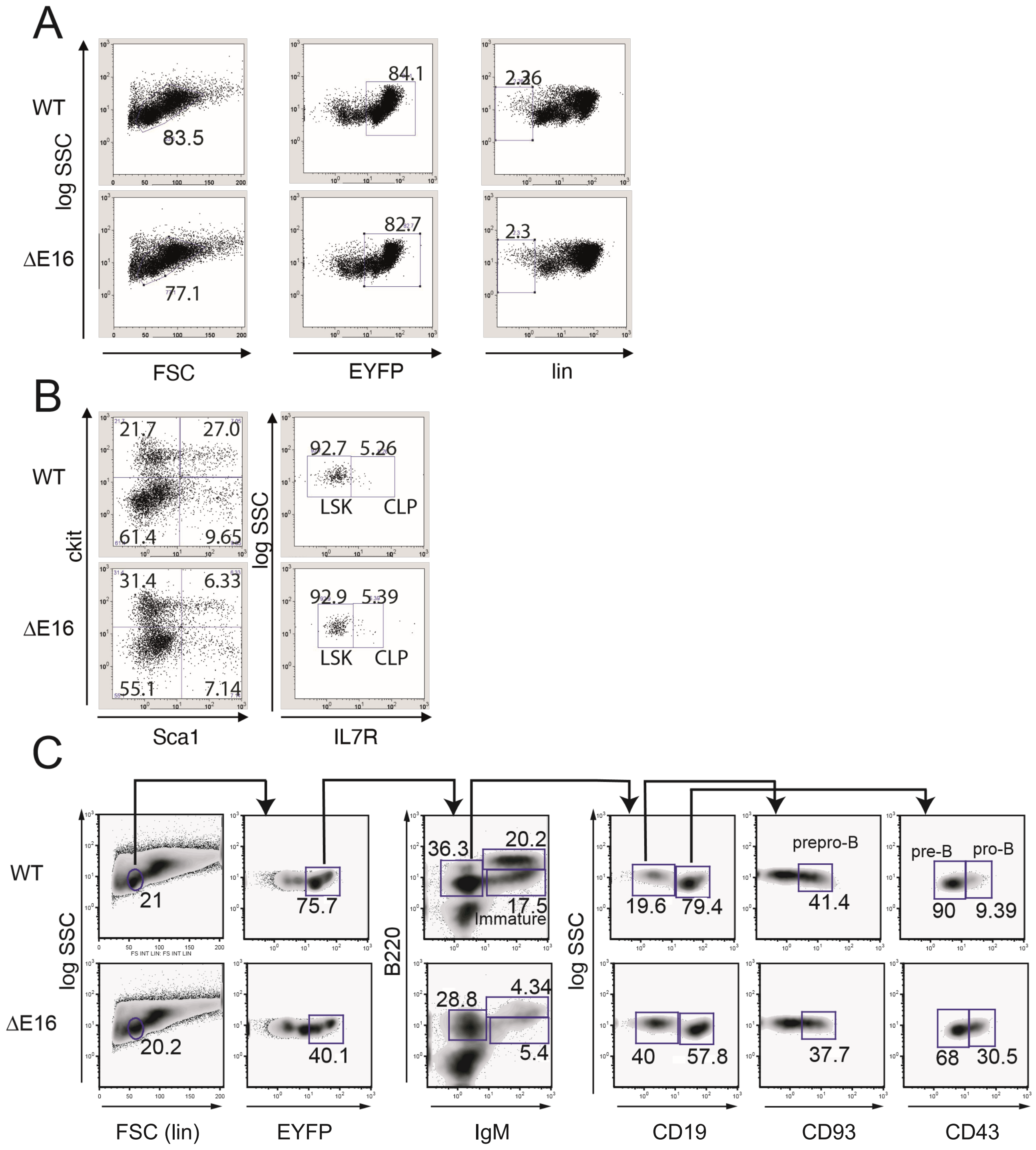

Supplementary Figure 2. Characterization strategy by flow cytometry for lymphoid progenitor populations in mouse bone marrow. A. Forward/side scatter (FSC/SSC) plot of total cells in WT and *Dido1*ΔE16 mice and EYFP+ cells. **B**. Within EYFP+ cells, lin- (Gr1- Ter119- CD11b-CD3-B220-) were analyzed for Sca1, cKit, and IL7R expression to characterize LSK (Sca1+cKit+IL7R-) and CLP (Sca1+cKit+IL7R+) populations. **C.** Forward/side scatter (FSC/SSC) plot of total cells in WT and *Dido1*ΔEI6 mice and EYFP+ cells. EYFP+ cells were gated by IgM and B220 markers to distinguish populations of mature EYFP+B220+IgMhigh cells and immature EYFP+B220+IgMlow cells. Of the EYFP+B220+IgM- subpopulation, prepro-B cells were identified as CD19-CD93+, pre-B as CD19+CD43-, and pro-B as CD19+CD43+.
