# Supplementary material for: The chromatin reader Dido3 is a regulator of the gene network that controls B cell differentiation
Source: Cell Biosci. 2025 Apr 26;15:56. doi: 10.1186/s13578-025-01394-x (PMC12034202; doi:10.1186/s13578-025-01394-x)
Supplement: Supplementary file 3 — Additional file3 (PDF 160 KB) [file 13578_2025_1394_MOESM3_ESM.pdf]

Supplementary Table 1

GSEA analysis of ATAC-seq data. GSEA analysis showing gensets from the full C2 collection (4762 gene sets) showing overlap with genes whose promoter have limited accessibility in Dido3-deficient hematopoietic stem cells, as identified by ATAC-seq. 50 genesets with lower false discovery rate (FDR) are listed.

Overlap Results.

Collection: C2 (“Curated genesets”) **50**

**# overlaps shown:**	**4762**
**# genesets in collections:**	**1098**
**genes in comparison:**	**45956**
**genes in universe:**	

Gene Set Name	# Genes in Gene Set (K)	Description	# Genes in Overlap (k)	k/K	p-value	FDR q-value
MEISSNER_BRAIN_HCP_WITH_H3K4ME3_AND_H3K27ME3	1069	H3 dimethylation at K4 (H3K4me2) and trimethylation at K27 (H3K27me3) in brain.	135	0,1263	2,66E-57	1,27E-53
BENPORATH_ES_WITH_H3K27ME3	1118	(H3K27me3) mark in their promoters in human embryonic stem cells, as identified by ChIP on chip.	113	0,1011	1,99E-38	4,74E-35
CHEN_METABOLIC_SYNDROM_NETWORK	1210	(MEMN) claimed to have a causal relationship with the metabolic syndrom traits.	111	0,0917	6,59E-34	1,05E-30
BENPORATH_EED_TARGETS	1062	The Polycomb protein EED [GeneID=8726] in human embryonic stem cells.	103	0,097	1,61E-33	1,91E-30
PEREZ_TP53_TARGETS	1174	Epithelium upon expression of TP53 [GeneID=7157] off	105	0,0894	3,65E-31	3,47E-28
BENPORATH_SUZ12_TARGETS	1038	Of the Polycomb protein SUZ12 [GeneID=23512] in human embryonic stem cells.	98	0,0944	5,56E-31	4,42E-28
SCHAEFFER_PROSTATE_DEVELOPMENT_48HR_DN	428	Females exposed to the androgen dihydrotestosterone [PubChem=10635] for 48 h.	63	0,1472	7,77E-31	5,28E-28
YOSHIMURA_MAPK8_TARGETS_UP	1305	MAPK8 (JNK1) [GeneID=5599].	107	0,082	1,42E-28	8,46E-26
NUYTTEN_EZH2_TARGETS_UP	1037	Knockdown of EZH2 [GeneID=2146] by RNAi.	94	0,0906	2,12E-28	1,12E-25
BLALOCK_ALZHEIMERS_DISEASE_UP	1691	Disease.	123	0,0727	5,72E-28	2,73E-25
WONG_ADULT_TISSUE_STEM_MODULE	721	Regulated in a compendium of adult tissue stem cells.	76	0,1054	3,21E-27	1,39E-24
MEISSNER_NPC_HCP_WITH_H3K4ME2	491	H3 dimethylation mark at K4 (H3K4me2) in neural precursor cells (NPC).	62	0,1263	1,29E-26	5,12E-24
CUI_TCF21_TARGETS_2_DN	830	Isolated from TCF21 [Gene ID=6943] knockout mice.	80	0,0964	5,08E-26	1,86E-23
LIM_MAMMARY_STEM_CELL_UP	489	Mouse and human species.	60	0,1227	3,92E-25	1,33E-22
MIKKELSEN_NPC_ICP_WITH_H3K4ME3	445	Histone H3 trimethylation mark at K4 (H3K4me3) in neural progenitor cells (NPC).	57	0,1281	7,01E-25	2,22E-22
KINSEY_TARGETS_OF_EWSR1_FLII_FUSION_DN	329	Genes down-regulated in TC71 and EWS502 cells (Ewing's sarcoma) by EWSR1-FLI1 [GeneID=2130;2314] as inferred from RNAi knockdown of this fusion protein.	48	0,1459	1,29E-23	3,83E-21
SMID_BREAST_CANCER_BASAL_DN	701	Genes down-regulated in basal subtype of breast cancer samples.	69	0,0984	4,22E-23	1,18E-20
BENPORATH_PRC2_TARGETS	652	Set 'PRC2 targets': Polycomb Repression Complex 2 (PRC) targets; identified by ChIP on chip on human embryonic stem cells as genes that: possess the trimethylated H3K27 mark in their promoters and are bound by SUZ12 [GeneID=23512] and EED [GeneID=8726] Polycomb proteins.	65	0,0997	4,00E-22	1,06E-19
LEE_BMP2_TARGETS_UP	745	Genes up-regulated in uterus upon knockout of BMP2 [GeneID=650].	69	0,0926	1,29E-21	3,23E-19
DODD_NASOPHARYNGEAL_CARCINOMA_UP	1821	Genes up-regulated in nasopharyngeal carcinoma (NPC) compared to the normal tissue.	114	0,0626	1,06E-20	2,52E-18
GOBERT_OLIGODENDROCYTE_DIFFERENTIATION_DN	1080	Genes down-regulated during differentiation of Oli-Neu cells (oligodendroglia precursor) in response to PD174265 [PubChem=4709].	83	0,0769	1,45E-20	3,30E-18
ZWANG_TRANSIENTLY_UP_BY_2ND_EGF_PULSE_ONLY	1725	Genes transiently induced only by the second pulse of EGF [GeneID =1950] in 184A1 cells (mammary epithelium).	109	0,0632	4,04E-20	8,74E-18
GOZGIT_ESR1_TARGETS_DN	781	Genes down-regulated in TMX2-28 cells (breast cancer) which do not express ESR1 [GeneID=2099]) compared to the parental MCF7 cells which do.	68	0,0871	7,21E-20	1,49E-17
GRAESSMANN_APOPTOSIS_BY_DOXORUBICIN_UP	1142	Genes up-regulated in ME-A cells (breast cancer) undergoing apoptosis in response to doxorubicin [PubChem=31703].	82	0,0718	1,51E-18	2,99E-16
SMID_BREAST_CANCER_LUMINAL_B_DN	564	Genes down-regulated in the luminal B subtype of breast cancer.	55	0,0975	1,78E-18	3,38E-16
SMID_BREAST_CANCER_BASAL_UP	648	Genes up-regulated in basal subtype of breast cancer samples.	59	0,091	2,63E-18	4,82E-16
LIU_PROSTATE_CANCER_DN	481	Genes down-regulated in prostate cancer samples.	50	0,104	4,61E-18	8,12E-16
DURAND_STROMA_S_UP	297	Genes up-regulated in the HSC supportive stromal cell lines.	39	0,1313	8,07E-18	1,37E-15
YANG_BCL3_TARGETS_UP	364	Genes up-regulated in neonatal cardiac myocytes upon knockdown of BCL3 [GeneID=602] by RNAi.	43	0,1181	9,24E-18	1,52E-15
CHICAS_RB1_TARGETS_CONFLUENT	567	Genes up-regulated in confluent IMR90 cells (fibroblast) after knockdown of RB1 [GeneID=5925] by RNAi.	54	0,0952	1,03E-17	1,64E-15
MEISSNER_NPC_HCP_WITH_H3_UNMETHYLATED	536	Genes with high-CpG-density promoters (HCP) that have no histone H3 methylation marks in neural precursor cells (NPC).	52	0,097	1,91E-17	2,88E-15
MIKKELSEN_ES_ICP_WITH_H3K4ME3	718	Genes with intermediate-CpG-density (ICP) promoters bearing histone H3 K4 trimethylation mark (H3K4me3) in embryonic stem cells (ES).	61	0,085	1,94E-17	2,88E-15
PLASARI_TGFB1_TARGETS_10HR_DN	244	Genes down-regulated in MEF cells (embryonic fibroblast) upon stimulation with TGFB1 [GeneID=7040] for 10 h.	35	0,1434	2,36E-17	3,40E-15

IVANOVA_HEMATOPOIESIS_STEM_CELL_AND_PROGENITOR	681	Genes in the expression cluster 'HSC and Progenitors Shared': up-regulated in hematopoietic stem cells (HSC) and progenitors from adult bone marrow and fetal liver.	59	0,0866	2,67E-17	3,74E-15
NUYTTEN_EZH2_TARGETS_DN	1024	Genes down-regulated in PC3 cells (prostate cancer) after knockdown of EZH2 [GeneID=2146] by RNAi.	74	0,0723	5,40E-17	7,35E-15
ZWANG_TRANSIENTLY_UP_BY_1ST_EGF_PULSE_ONLY	1839	Genes transiently induced only by the first pulse of EGF [GeneID=1950] in 184A1 cells (mammary epithelium).	106	0,0576	8,61E-17	1,13E-14
CREIGHTON_ENDOCRINE_THERAPY_RESISTANCE_3	720	The 'group 3 set' of genes associated with acquired endocrine therapy resistance in breast tumors expressing ESR1 and ERBB2 [GeneID=2099;2064].	60	0,0833	8,75E-17	1,13E-14
MCBRYAN_PUBERTAL_BREAST_4_5WK_UP	271	Genes up-regulated during pubertal mammary gland development between week 4 and 5.	36	0,1328	1,02E-16	1,27E-14
ONDER_CDH1_TARGETS_2_DN	464	Genes down-regulated in HMLE cells (immortalized non-transformed mammary epithelium) after E-cadhedrin (CDH1) [GeneID=999] knockdown by RNAi.	47	0,1013	1,28E-16	1,56E-14
LINDGREN_BLADDER_CANCER_CLUSTER_2B	392	Genes specifically up-regulated in Cluster IIb of urothelial cell carcinoma (UCC) tumors.	43	0,1097	1,43E-16	1,71E-14
GRYDER_PAX3FOXO1_ENHANCERS_IN_TADS	975	Expressed genes (FPKM>1) associated with high-confidence PAX3-FOXO1 sites with enhancers in primary tumors and cell lines, restricted to those within topological domain boundaries	71	0,0728	1,60E-16	1,86E-14
RIGGI_EWING_SARCOMA_PROGENITOR_UP	430	Genes up-regulated in mesenchymal stem cells (MSC) engineered to express EWS-FLI1 [GeneID=2130;2321] fusion protein.	45	0,1047	1,68E-16	1,90E-14
WEST_ADRENOCORTICAL_TUMOR_DN	546	Down-regulated genes in pediatric adrenocortical tumors (ACT) compared to the normal tissue.	51	0,0934	1,87E-16	2,08E-14
NABA_MATRISOME	1028	Ensemble of genes encoding extracellular matrix and extracellular matrix-associated proteins	73	0,071	2,22E-16	2,41E-14
BAELDE_DIABETIC_NEPHROPATHY_DN	434	Genes down-regulated in glomeruli of kidneys from patients with diabetic nephropathy (type 2 diabetes mellitus).	45	0,1037	2,38E-16	2,52E-14
RODRIGUES_THYROID_CARCINOMA_POORLY_DIFFERENTIATED_DN	805	Genes down-regulated in poorly differentiated thyroid carcinoma (PDTC) compared to normal thyroid tissue.	63	0,0783	2,97E-16	3,07E-14
BLALOCK_ALZHEIMERS_DISEASE_DN	1237	Genes down-regulated in brain from patients with Alzheimer's disease.	81	0,0655	4,81E-16	4,87E-14
BOQUEST_STEM_CELL_CULTURED_VS_FRESH_UP	425	Genes up-regulated in cultured stromal stem cells from adipose tissue, compared to the freshly isolated cells.	44	0,1035	5,42E-16	5,37E-14
ACEVEDO_FGFR1_TARGETS_IN_PROSTATE_CANCER_MODEL_DN	308	Genes down-regulated during prostate cancer progression in the JOCK1 model due to inducible activation of FGFR1 [GeneID=2260] gene in prostate.	37	0,1201	1,04E-15	1,01E-13
BONOME_OVARIAN_CANCER_SURVIVAL_SUBOPTIMAL_DEBULKING	510	Genes whose expression in sub optimally debulked ovarian tumors is associated with survival prognosis.	48	0,0941	1,08E-15	1,03E-13
