# Supplementary material for: The chromatin reader Dido3 is a regulator of the gene network that controls B cell differentiation
Source: Cell Biosci. 2025 Apr 26;15:56. doi: 10.1186/s13578-025-01394-x (PMC12034202; doi:10.1186/s13578-025-01394-x)
Supplement: Supplementary file 4 — Additional file4 (PDF 458 KB) [file 13578_2025_1394_MOESM4_ESM.pdf]

Supplementary Table 2

Differentially expressed transcripts (RNA-seq data). Lists of up- and down-regulated genes in Dido3-deficient pre-B cells in comparation to control WT. Log2 of fold-change (logFC), log2 of counts per million (logCPM), fragments per kilobase of exon per million mapped fragments (F) and statistical significance as t-test p-value and false discovery rate (FDR) are included. Lists are limited to genes with FDR<0.05.

Upregulated in *Dido1*ΔE16 pre-B						
**TranscriptID**	**Symbol**	**logFC**	**logCPM**	**F**	**PValue**	**FDR**
**ENSMUST00000044123.1**	Trhr2	8.178254997	3.689639849	291.5462108	2.38E-65	1.69E-60
**ENSMUST00000036215.7**	Foxj1	5.05070799	4.24479595	240.3549946	3.38E-54	1.6E-49
**ENSMUST00000069741.3**	E130018O15Rik	7.328231004	3.400752558	227.4450916	2.97E-51	1.06E-46
**ENSMUST00000045068.9**	Cplx3	8.373271374	3.009840974	200.5853343	1.8E-45	5.13E-41
**ENSMUST00000043315.14**	Serpina3g	2.670885696	6.717498823	193.9837055	4.37E-44	1.04E-39
**ENSMUST00000000199.7**	Ncs1	6.476854223	3.228629155	186.3587587	2.01E-42	4.1E-38
**ENSMUST00000149378.1**	Gm13398	6.422683528	3.251844647	184.800757	5.98E-42	1.06E-37
**ENSMUST00000201921.1**	4930478P22Rik	4.849938561	3.724752284	171.9832757	2.77E-39	4.38E-35
**ENSMUST00000003501.8**	Elavl3	6.145498595	3.091770125	164.8498319	9.99E-38	1.42E-33
**ENSMUST00000145549.1**	B230312C02Rik	4.950126862	4.267465985	215.6493373	4.32E-37	5.59E-33
**ENSMUST00000100396.3**	4930407I10Rik	6.007672773	3.012110222	152.6974129	4.51E-35	5.35E-31
**ENSMUST00000203350.1**	Gm44067	2.829409924	5.51242358	148.3735112	3.97E-34	4.35E-30
**ENSMUST00000098778.2**	Gm10676	2.975303294	5.190339212	145.529287	1.66E-33	1.69E-29
**ENSMUST00000114973.8**	Kalrn	4.193488629	3.806229123	144.124296	3.37E-33	3.2E-29
**ENSMUST00000145988.8**	Dnhd1	2.979992739	5.009071296	137.1784691	1.11E-31	9.9E-28
**ENSMUST00000179531.1**	Rnpepl1	2.766281975	5.374760616	135.2117135	2.99E-31	2.51E-27
**ENSMUST00000181436.1**	Gm26583	6.580283705	2.667763471	135.657234	3.65E-31	2.89E-27
**ENSMUST00000093832.10**	Lman1l	8.327862413	2.278974964	129.007599	6.81E-30	5.1E-26
**ENSMUST00000060125.6**	Scn4b	2.689071631	5.386890346	128.0014945	5.13E-29	3.65E-25
**ENSMUST00000017637.12**	Igfbp4	2.865737935	4.840380216	120.4695138	5.03E-28	3.41E-24
**ENSMUST00000057612.8**	Ssc5d	4.502593079	3.19675086	117.2099648	2.6E-27	1.68E-23
**ENSMUST00000187142.2**	Zfp469	7.556092546	2.19423831	116.6247506	3.49E-27	2.11E-23
**ENSMUST00000046892.9**	Cplx1	6.350367745	2.47891755	116.6594687	3.55E-27	2.11E-23
**ENSMUST00000207625.1**	A930030B08Rik	5.274463635	2.786384842	113.8938095	1.38E-26	7.88E-23
**ENSMUST00000176061.1**	Gm20681	8.12726194	2.058727587	112.3932968	2.95E-26	1.61E-22
**ENSMUST00000114574.2**	Glp1r	6.747750682	2.213000516	108.8820554	1.73E-25	9.14E-22
**ENSMUST00000144818.1**	Gm14168	3.243553083	4.087137291	107.3749792	3.7E-25	1.88E-21
**ENSMUST00000231439.1**	Igll1	3.654322209	3.723318304	109.8775049	5.94E-25	2.92E-21
**ENSMUST00000103430.1**	Ighj1	4.142917982	3.161137658	101.0360908	9.07E-24	4.17E-20
**ENSMUST00000030551.10**	Alpl	2.024903863	6.33004764	100.9736693	9.76E-24	4.34E-20
**ENSMUST00000088904.9**	Espnl	5.864652669	2.312617787	100.0042741	1.53E-23	6.59E-20
**ENSMUST00000073935.6**	Gsg1l	4.583973176	2.863310035	99.65603652	1.82E-23	7.63E-20
**ENSMUST00000231330.1**	Gm35455	7.257248574	1.915417082	96.70873365	8.07E-23	3.28E-19
**ENSMUST00000044970.6**	Mgat3	7.248207023	1.912963946	96.40751334	9.39E-23	3.72E-19
**ENSMUST00000080368.12**	Atp8a2	4.772741967	2.689835341	95.81534358	1.27E-22	4.81E-19
**ENSMUST00000045085.7**	Grin3b	3.645437838	3.469184124	95.7902219	1.28E-22	4.81E-19
**ENSMUST00000044384.4**	Aldh1b1	1.75479016	7.28316449	94.78412823	2.15E-22	7.85E-19
**ENSMUST00000065587.4**	Ackr3	3.26082333	3.812359919	93.99867194	3.47E-22	1.24E-18
**ENSMUST00000046383.11**	Tnfsf10	2.627373387	4.558816641	92.17799591	1.56E-21	5.42E-18
**ENSMUST00000095360.10**	Igf1	4.022933516	3.03523223	89.80536709	2.64E-21	8.94E-18
**ENSMUST00000144897.1**	Slx1b	2.44672511	4.826217701	88.32164445	5.58E-21	1.85E-17
**ENSMUST00000203263.1**	Gm44066	2.643559159	4.429725713	87.93388583	6.79E-21	2.2E-17
**ENSMUST00000009875.4**	Kcnd1	3.878502091	3.060776876	86.71730507	1.26E-20	3.98E-17
**ENSMUST00000049614.12**	B430306N03Rik	2.477422298	4.677193091	85.91677149	1.88E-20	5.83E-17
**ENSMUST00000070328.9**	Sh2d4b	2.756022319	4.209991815	84.79227113	3.32E-20	1.01E-16
**ENSMUST00000140833.1**	Gm15728	5.546501016	2.104448451	82.37768727	1.13E-19	3.28E-16
**ENSMUST00000000188.11**	Ccnd2	1.818841158	6.329194025	81.45457634	1.83E-19	5.21E-16
**ENSMUST00000057831.7**	Cilp2	5.029747674	2.245706746	81.06735435	2.19E-19	6.11E-16
**ENSMUST00000180086.2**	H1f0	1.595263421	7.333076762	78.90285205	6.54E-19	1.79E-15
**ENSMUST00000022699.9**	Gfra2	2.926129741	3.827212439	78.58993845	7.71E-19	2.07E-15

**ENSMUST00000040821.4**	Heyl	2.045638174	5.54571685	79.12551615	1.17E-18	3.07E-15
**ENSMUST00000054491.5**	Sox18	8.857512753	1.34795791	77.4372545	1.37E-18	3.56E-15
**ENSMUST00000179520.1**	Ighd4-1	4.181337013	2.652040852	77.36235869	1.43E-18	3.63E-15
**ENSMUST00000025830.8**	Apba1	2.83487618	3.933545204	77.33293058	1.47E-18	3.68E-15
**ENSMUST00000033054.9**	Adm	2.745857335	4.051061047	77.31621369	1.52E-18	3.73E-15
**ENSMUST00000105572.2**	Perm1	2.736342512	4.010776099	76.00926352	2.83E-18	6.83E-15
**ENSMUST00000030420.8**	Epha8	8.802780653	1.308029304	75.36574302	3.92E-18	9.31E-15
**ENSMUST00000145401.7**	Il9r	3.600406092	3.923795062	100.1664368	6.83E-18	1.59E-14
**ENSMUST00000166193.8**	Igfn1	6.853215762	1.529374109	74.07841672	7.53E-18	1.73E-14
**ENSMUST00000020537.8**	Nsg2	1.765745532	6.112490555	71.46610797	2.83E-17	6.39E-14
**ENSMUST00000206494.1**	Unc45a	3.446151321	3.057578002	70.31115962	5.08E-17	1.13E-13
**ENSMUST00000027533.8**	Klhl30	6.312169778	1.524034391	68.46552634	1.29E-16	2.79E-13
**ENSMUST00000159547.2**	Vamp1	1.542047785	6.889653481	67.41663009	2.2E-16	4.68E-13
**ENSMUST00000185943.1**	5031425E22Rik	3.323723001	3.043311935	65.79335522	5.04E-16	1.06E-12
**ENSMUST00000028689.3**	Lrp4	3.288327318	3.094569561	65.64168988	5.42E-16	1.12E-12
**ENSMUST00000051765.8**	Glp2r	4.982508838	1.935230947	65.5190259	5.8E-16	1.18E-12
**ENSMUST00000017561.14**	Plxdc1	2.130467259	4.719182841	64.85862685	8.06E-16	1.62E-12
**ENSMUST00000075317.11**	Pdzd2	1.577930631	6.475284059	63.91053675	1.41E-15	2.79E-12
**ENSMUST00000236745.1**	Ubash3a	3.015582757	3.297585315	63.06607012	2E-15	3.91E-12
**ENSMUST00000018711.14**	Gabarap	2.282096628	4.308942553	62.17482126	3.15E-15	6.06E-12
**ENSMUST00000127305.1**	Epn3	8.460520852	1.009187845	61.83380794	3.74E-15	7.11E-12
**ENSMUST00000097648.5**	Ramp1	2.138698729	4.519124664	60.58985181	7.04E-15	1.32E-11
**ENSMUST00000192126.1**	Gm18407	2.775747462	3.512532719	59.74526766	1.08E-14	2E-11
**ENSMUST00000102942.7**	Psd4	1.726891331	5.629282353	58.2842288	2.27E-14	4.15E-11
**ENSMUST00000126198.2**	Fam78b	5.812836728	1.343676636	58.01062587	2.61E-14	4.71E-11
**ENSMUST00000196046.1**	Gm43364	6.479984579	1.171433088	57.88118167	2.79E-14	4.97E-11
**ENSMUST00000097474.8**	Rcsd1	2.233583036	4.159368437	54.99921882	1.21E-13	2.12E-10
**ENSMUST00000132422.1**	Gps1	3.284351465	2.758402058	54.54650402	1.52E-13	2.64E-10
**ENSMUST00000053856.5**	Pcdhb17	3.31656198	2.705065885	53.65228999	2.4E-13	4.06E-10
**ENSMUST00000132080.1**	Tmem259	2.905692276	3.118682753	53.42706116	2.69E-13	4.5E-10
**ENSMUST00000052528.4**	Gm9847	3.624529389	2.40044788	52.78942758	3.73E-13	6.17E-10
**ENSMUST00000026315.7**	Dnase1l3	1.541661805	6.019372997	52.69721816	3.9E-13	6.38E-10
**ENSMUST00000137906.1**	Arid5a	1.883715516	4.820906235	52.59156905	4.11E-13	6.66E-10
**ENSMUST00000103091.8**	Elmo2	3.524071926	2.440161058	52.32854761	4.7E-13	7.52E-10
**ENSMUST00000075827.4**	Jag2	2.709793846	3.29670242	51.52895184	7.06E-13	1.12E-09
**ENSMUST00000016172.8**	Celsr1	3.06916754	2.851172513	51.14074038	8.61E-13	1.35E-09
**ENSMUST00000134427.1**	Gm11613	2.334296872	3.824140741	50.75561061	1.05E-12	1.6E-09
**ENSMUST00000108567.8**	Zfp444	2.085246088	4.243471562	50.20504135	1.39E-12	2.1E-09
**ENSMUST00000029658.13**	Enpep	2.509797611	3.483001415	49.1351093	2.39E-12	3.59E-09
**ENSMUST00000060474.13**	Septin6	1.537678051	5.80830831	48.91053335	2.68E-12	3.98E-09
**ENSMUST00000000153.8**	Gna12	1.332689888	6.748772682	48.70528104	2.98E-12	4.37E-09
**ENSMUST00000071135.5**	Tubb4a	2.778146887	3.099110788	48.58844318	3.16E-12	4.59E-09
**ENSMUST00000211765.1**	Nucb1	1.823622664	4.731981699	47.86251092	4.58E-12	6.52E-09
**ENSMUST00000202026.3**	4930478P22Rik	4.326020601	1.697352809	47.61659668	5.2E-12	7.34E-09
**ENSMUST00000030585.7**	A3galt2	3.901896341	1.92571106	47.4104692	5.78E-12	8.07E-09
**ENSMUST00000020768.3**	Pgam2	1.511219167	5.805748496	47.19041525	6.45E-12	8.83E-09
**ENSMUST00000030677.6**	Map3k6	5.128849384	1.20890097	47.07878579	6.83E-12	9.26E-09
**ENSMUST00000025805.7**	Cnih2	4.390761895	1.571238692	46.78615298	7.93E-12	1.06E-08
**ENSMUST00000039752.3**	Slc16a8	4.955339846	1.298610136	46.7491308	8.08E-12	1.08E-08
**ENSMUST00000211939.1**	Polr2c	3.127553302	2.612228058	46.30081159	1.02E-11	1.34E-08
**ENSMUST00000024575.7**	Rps6ka2	1.724961592	4.949365839	46.22813017	1.05E-11	1.38E-08
**ENSMUST00000127208.7**	Lrrc14	2.921898408	2.831551061	46.08399964	1.13E-11	1.47E-08
**ENSMUST00000037007.3**	Evpl	3.199786031	2.525025391	45.620841	1.44E-11	1.85E-08
**ENSMUST00000055619.4**	Hic1	3.146427583	2.54680267	45.31331751	1.68E-11	2.1E-08
**ENSMUST00000132562.1**	Wrap73	3.651890768	2.045044936	44.96172096	2.01E-11	2.47E-08
**ENSMUST00000224954.1**	Daam2	2.639852539	3.123062049	44.50121968	2.54E-11	3.1E-08
**ENSMUST00000047282.11**	Mthfsd	1.706383836	4.895833979	44.40456761	2.67E-11	3.23E-08
**ENSMUST00000046937.3**	Tssk1	4.152835828	1.611894621	43.99331675	3.3E-11	3.95E-08
**ENSMUST00000232008.1**	4933432I09Rik	4.123826935	1.644247453	43.5698768	4.09E-11	4.86E-08

**ENSMUST00000040059.8**	Hyal3	4.306498157	1.494729643	43.48327016	4.28E-11	5.04E-08
**ENSMUST00000055872.2**	Galr2	3.091181839	2.541521959	43.07381194	5.28E-11	6.11E-08
**ENSMUST00000109764.7**	Nfix	1.7474685	4.639411263	42.50577762	7.05E-11	8.1E-08
**ENSMUST00000120430.1**	Gm7901	1.119022712	7.808153921	42.14990359	8.46E-11	9.64E-08
**ENSMUST00000184613.1**	Mrip-ps	1.188132892	7.181988937	42.07389417	8.81E-11	9.97E-08
**ENSMUST00000122029.1**	Gm6939	3.104980149	2.396644455	41.14392486	1.42E-10	1.58E-07
**ENSMUST00000047309.5**	Nat14	2.795271917	2.755541996	40.68164561	1.79E-10	1.98E-07
**ENSMUST00000201142.3**	1700028E10Rik	2.743697883	2.808371646	40.61036861	1.86E-10	2.04E-07
**ENSMUST00000053926.11**	Gm49486	4.520082979	1.257922682	40.51833229	1.95E-10	2.1E-07
**ENSMUST00000032877.10**	Ddias	1.522241681	5.29710636	40.41477923	2.06E-10	2.21E-07
**ENSMUST00000165838.8**	Metrn	2.013891208	3.934040724	40.28601272	2.2E-10	2.33E-07
**ENSMUST00000238352.1**	Gm10570	4.65685676	1.140663365	40.24497226	2.24E-10	2.37E-07
**ENSMUST00000099373.11**	Cnnm2	3.11348923	2.358574148	40.20048908	2.29E-10	2.4E-07
**ENSMUST00000206275.1**	Bola2	2.04896394	3.857536965	40.12932128	2.38E-10	2.47E-07
**ENSMUST00000086281.4**	Zfp599	2.204170722	3.581558944	40.03868374	2.49E-10	2.57E-07
**ENSMUST00000095076.9**	Epb41l4b	3.467940505	1.959970582	39.68324766	2.99E-10	3.06E-07
**ENSMUST00000112682.3**	Slc25a18	1.746638239	4.461506871	39.37063282	3.51E-10	3.56E-07
**ENSMUST00000057311.3**	Sfn	1.293032336	6.207664903	39.23106449	3.79E-10	3.8E-07
**ENSMUST00000225289.1**	Rab24	3.467489401	1.933190752	38.68112905	4.99E-10	4.94E-07
**ENSMUST00000153578.7**	Tspoap1	1.991732965	3.934592249	39.14459395	5.11E-10	5.02E-07
**ENSMUST00000168386.8**	Prr36	4.461707258	1.163163503	38.15753377	6.53E-10	6.33E-07
**ENSMUST00000086040.5**	F5	1.239453192	7.159198127	41.20653877	7.58E-10	7.2E-07
**ENSMUST00000204059.2**	Add2	3.308950451	2.010560846	37.76298621	7.99E-10	7.54E-07
**ENSMUST00000051442.6**	Pcdhb16	2.981830872	2.384961332	37.70910916	8.22E-10	7.7E-07
**ENSMUST00000179719.1**	Hyi	2.303165648	3.285830862	37.63990033	8.51E-10	7.92E-07
**ENSMUST00000194216.1**	Gm37795	2.443973485	3.052818063	37.35251713	9.87E-10	8.95E-07
**ENSMUST00000201742.1**	Gm43860	1.504986218	5.056842678	36.35552456	1.65E-09	1.47E-06
**ENSMUST00000039840.14**	Enpp6	2.810178276	2.502889818	36.13162121	1.85E-09	1.64E-06
**ENSMUST00000028403.2**	Cybrd1	2.701860557	2.708552249	36.43646512	1.87E-09	1.65E-06
**ENSMUST00000056890.9**	Fbxl22	1.324652146	5.787102885	35.89699984	2.08E-09	1.83E-06
**ENSMUST00000030142.3**	Epb41l4b	1.114166377	7.034874192	35.83237583	2.15E-09	1.88E-06
**ENSMUST00000204971.1**	Pyroxd1	3.535965666	1.681534547	35.46673706	2.6E-09	2.23E-06
**ENSMUST00000055436.4**	Hpdl	4.395973385	1.110148268	35.18551657	3E-09	2.56E-06
**ENSMUST00000217282.1**	Gm38431	3.047412451	2.155405091	35.14967029	3.05E-09	2.59E-06
**ENSMUST00000026985.8**	Cplx2	0.786366167	11.12044583	35.08636215	3.16E-09	2.66E-06
**ENSMUST00000062117.13**	Rap2a	1.823566263	4.054593856	35.0695298	3.18E-09	2.67E-06
**ENSMUST00000150442.1**	Gm11642	3.953466338	1.331947062	34.99043619	3.32E-09	2.76E-06
**ENSMUST00000115421.2**	Steap4	0.906258524	9.153220041	34.91076411	3.45E-09	2.86E-06
**ENSMUST00000023259.14**	Lynx1	0.872210902	9.622204881	34.75964575	3.76E-09	3.06E-06
**ENSMUST00000050519.7**	Elfn1	4.348535247	1.06872759	33.9158706	5.77E-09	4.64E-06
**ENSMUST00000238703.1**	Gm50464	3.843958267	1.322656373	33.29999737	7.9E-09	6.22E-06
**ENSMUST00000180612.2**	9330175E14Rik	1.438984143	5.022930753	32.87358614	9.84E-09	7.7E-06
**ENSMUST00000128342.1**	Gm16576	1.670149231	4.238729656	32.30430049	1.32E-08	1.02E-05
**ENSMUST00000103105.9**	Aoc3	3.313840055	1.722766459	32.07082628	1.49E-08	1.15E-05
**ENSMUST00000077502.4**	Dqx1	1.869603207	3.750285324	31.75555964	1.75E-08	1.33E-05
**ENSMUST00000066330.14**	Mprip	1.240870191	5.813474083	31.70793684	1.79E-08	1.36E-05
**ENSMUST00000123349.1**	Gramd4	1.580677503	4.425720133	31.62199216	1.87E-08	1.41E-05
**ENSMUST00000027809.7**	Opn3	1.488250879	4.718897142	31.57717888	1.92E-08	1.42E-05
**ENSMUST00000101339.7**	Nhsl2	1.775886223	3.947564817	31.42541421	2.07E-08	1.52E-05
**ENSMUST00000201164.1**	4930553P18Rik	2.691706319	2.400043714	31.37788503	2.12E-08	1.55E-05
**ENSMUST00000066386.5**	Lysmd1	1.916718853	3.643093553	31.3707699	2.13E-08	1.55E-05
**ENSMUST00000192173.1**	Gm37233	1.756222894	3.963099576	31.10297182	2.45E-08	1.76E-05
**ENSMUST00000209248.1**	Rnf223	2.456071228	2.702815818	31.03311763	2.54E-08	1.82E-05
**ENSMUST00000039178.11**	Tnn	3.021037311	1.966058195	30.76333939	2.92E-08	2.08E-05
**ENSMUST00000165968.1**	Serpina3e-ps	2.644285216	2.411907374	30.63693545	3.11E-08	2.21E-05
**ENSMUST00000027952.11**	Plxna2	2.451827751	2.679388168	30.55589348	3.25E-08	2.29E-05
**ENSMUST00000142367.7**	Palm3	3.415190644	1.474732627	30.13047218	4.05E-08	2.8E-05
**ENSMUST00000231640.1**	Gm49745	2.835067494	2.0716474	29.54989546	5.45E-08	3.75E-05
**ENSMUST00000087497.10**	Col11a2	1.918409716	3.511668596	29.49297622	5.61E-08	3.83E-05

**ENSMUST00000119494.2**	Plk-ps1	1.649705765	4.078484833	29.00786005	7.21E-08	4.87E-05
**ENSMUST00000048923.6**	Spred3	2.233625227	2.883617794	28.47234464	9.51E-08	6.22E-05
**ENSMUST00000226062.1**	Psd	1.382994821	4.796589922	28.04201831	1.19E-07	7.62E-05
**ENSMUST00000110052.1**	Ocel1	1.202401787	5.630785468	28.02148255	1.2E-07	7.67E-05
**ENSMUST00000159283.7**	Manf	2.099437434	3.071940068	27.73969493	1.39E-07	8.75E-05
**ENSMUST00000232332.2**	Gm1043	1.515355313	4.323381884	27.56396107	1.52E-07	9.5E-05
**ENSMUST00000051259.9**	Adgrg3	3.149464432	1.582950951	27.5342098	1.54E-07	9.6E-05
**ENSMUST00000036248.12**	Pmepa1	1.889435097	3.406260219	27.11245222	1.92E-07	0.000116893
**ENSMUST00000130268.7**	Mypop	1.651725153	3.9274394	26.93075453	2.11E-07	0.000126255
**ENSMUST00000108288.8**	Lrfn1	2.648980494	2.118020507	26.87884245	2.17E-07	0.00012915
**ENSMUST00000232529.1**	Car15	2.190762771	2.816033678	26.49825842	2.64E-07	0.000154042
**ENSMUST00000093369.4**	Nefh	3.255859372	1.408825797	26.37595979	2.81E-07	0.000162776
**ENSMUST00000123325.8**	Ankrd33b	1.385163378	4.612731563	26.3481133	2.85E-07	0.000163808
**ENSMUST00000180852.1**	2610037D02Rik	2.883952188	1.80721041	26.32098562	2.89E-07	0.000165457
**ENSMUST00000204482.2**	Tmem176a	2.954310878	1.69945943	26.26396613	2.98E-07	0.000169733
**ENSMUST00000058479.6**	Drc7	2.627568213	2.080968669	25.83238963	3.73E-07	0.000208904
**ENSMUST00000149932.1**	Gm13184	3.001670523	1.615375774	25.43483112	4.58E-07	0.000252715
**ENSMUST00000006669.5**	Pdk1	1.598024162	3.934299237	25.38804423	4.69E-07	0.000258066
**ENSMUST00000023072.6**	Parvb	1.80953032	3.435541445	25.33830344	4.82E-07	0.00026396
**ENSMUST00000177908.1**	Cfap73	1.863204877	3.343751007	25.25257978	5.03E-07	0.0002737
**ENSMUST00000054487.9**	Ajuba	3.432751962	1.148390741	25.21224091	5.14E-07	0.000278234
**ENSMUST00000125447.2**	Macf1	1.488815289	4.207830645	25.15140066	5.31E-07	0.000286305
**ENSMUST00000024860.8**	Ehd3	1.300951319	4.815575882	24.90150471	6.04E-07	0.000321987
**ENSMUST00000110218.8**	Spef1	1.779618352	3.473243744	24.82857175	6.27E-07	0.000333154
**ENSMUST00000193564.1**	Gm37736	2.774531055	1.80628983	24.78582995	6.41E-07	0.000339357
**ENSMUST00000041124.12**	Zfp704	1.471828907	4.208156849	24.66828683	6.82E-07	0.000359985
**ENSMUST00000028223.8**	Kynu	1.607251987	3.812534738	24.11986018	9.05E-07	0.00046902
**ENSMUST00000065793.11**	Phgdh	0.978825536	6.451178839	24.04023603	9.44E-07	0.00048529
**ENSMUST00000163139.7**	Plxna1	1.300548345	4.708694186	23.97406199	9.77E-07	0.000498658
**ENSMUST00000104937.1**	Ankrd63	3.182732563	1.266945918	23.91952368	1E-06	0.000509172
**ENSMUST00000003154.6**	Efna2	2.856305497	1.64539989	23.91329801	1.01E-06	0.000509172
**ENSMUST00000124126.1**	Polr1c	3.319268229	1.183690409	23.8860097	1.02E-06	0.000514616
**ENSMUST00000020549.3**	Gzmm	2.539601573	2.0484197	23.59066076	1.19E-06	0.000593671
**ENSMUST00000022921.6**	Angpt1	1.366028426	4.423730217	23.43669635	1.29E-06	0.000638663
**ENSMUST00000133221.2**	Trp53cor1	3.098395699	1.320261454	23.37125186	1.34E-06	0.000656207
**ENSMUST00000218571.1**	Map2k2	2.343529236	2.300906166	23.26372174	1.41E-06	0.000689183
**ENSMUST00000119613.1**	Gm5939	1.081571173	5.721270725	23.24257768	1.43E-06	0.000694423
**ENSMUST00000094451.3**	Gpr157	2.000914006	2.899214486	23.10041596	1.54E-06	0.00074516
**ENSMUST00000045713.3**	Nacad	1.397895867	4.270906699	22.90602162	1.7E-06	0.000816118
**ENSMUST00000059026.9**	Abi3	2.459223566	2.102308684	22.78780396	1.81E-06	0.000856356
**ENSMUST00000004036.5**	Efnb3	1.593601349	3.729015516	22.77231922	1.82E-06	0.000860425
**ENSMUST00000200257.1**	Gm19710	2.389135468	2.183344783	22.75505761	1.84E-06	0.000865322
**ENSMUST00000152138.7**	Casc1	3.209823198	1.201112581	22.7080501	1.89E-06	0.000883833
**ENSMUST00000187230.1**	Begain	3.058379859	1.290756938	22.67051025	1.92E-06	0.000898314
**ENSMUST00000217354.1**	Ppp2r3d	1.490085117	3.979783517	22.51386827	2.09E-06	0.000965129
**ENSMUST00000154277.1**	Galr2	2.720036002	1.686457289	22.47970309	2.12E-06	0.000979268
**ENSMUST00000208686.1**	Prcp	2.196527545	2.490823108	22.42829885	2.18E-06	0.001002584
**ENSMUST00000122882.1**	2210406O10Rik	3.271197846	1.046784841	22.15524491	2.52E-06	0.001144697
**ENSMUST00000014118.3**	Mcemp1	2.659118377	1.757489807	22.08215972	2.61E-06	0.001182165
**ENSMUST00000231165.1**	Nfam1	1.226158307	4.800452911	21.91974	2.84E-06	0.001273771
**ENSMUST00000159004.1**	Mrgpra2a	2.648018483	1.876412857	22.36282257	2.92E-06	0.001304577
**ENSMUST00000026886.7**	Itih5	1.178633588	5.016252047	21.81641371	3E-06	0.001331674
**ENSMUST00000211974.1**	Adgrg3	2.726435361	1.610165285	21.80186389	3.02E-06	0.001337643
**ENSMUST00000234131.1**	Slc8a1	2.580137573	1.850693752	21.79559037	3.03E-06	0.001337869
**ENSMUST00000033495.14**	Pim2	0.844940609	7.347218913	21.6973006	3.19E-06	0.001398219
**ENSMUST00000070084.10**	Ticam2	1.160760069	5.086970346	21.67381182	3.23E-06	0.001408107
**ENSMUST00000074408.6**	Ifnlr1	2.184659642	2.438989006	21.6521755	3.27E-06	0.001419737
**ENSMUST00000082432.4**	Dio2	2.000961368	2.76544012	21.60887215	3.34E-06	0.001443352
**ENSMUST00000193350.1**	Gm37120	1.369415633	4.236353842	21.57156364	3.41E-06	0.001463789

**ENSMUST00000041093.5**	Creb3l2	0.815078195	7.689664066	21.57031547	3.41E-06	0.001463789
**ENSMUST00000028780.3**	Chac1	2.542078207	1.852268354	21.47526434	3.59E-06	0.001528955
**ENSMUST00000142665.1**	Wrap73	1.614515928	3.569008251	21.40444732	3.72E-06	0.001581742
**ENSMUST00000060524.10**	Trim10	2.23280333	2.290065577	21.04537315	4.5E-06	0.001881433
**ENSMUST00000069817.14**	Prrc2b	0.921954593	6.411183435	20.99414127	4.62E-06	0.001923849
**ENSMUST00000193194.1**	AA914427	3.146122504	1.118916616	20.98483954	4.63E-06	0.001923849
**ENSMUST00000169652.2**	Tifab	1.622130984	3.485237747	20.70558945	5.36E-06	0.002193104
**ENSMUST00000237716.1**	Gramd3	1.704791207	3.267133208	20.41862597	6.22E-06	0.002504222
**ENSMUST00000159977.1**	Pcmt1	2.424066666	1.933994746	20.35431869	6.44E-06	0.002568039
**ENSMUST00000024774.13**	Guca1b	2.026143064	2.60078282	20.21724586	6.91E-06	0.002751059
**ENSMUST00000019068.6**	Alox15	2.4872212	1.833073366	20.19116051	7.41E-06	0.002922246
**ENSMUST00000105545.11**	Phactr2	1.552111795	3.596681038	20.06800061	7.48E-06	0.002941441
**ENSMUST00000069324.6**	Zfp580	1.759429981	3.099546922	19.90546691	8.14E-06	0.003167394
**ENSMUST00000182751.1**	Rbpj-ps3	0.962880195	5.957685839	19.81859605	8.52E-06	0.003305615
**ENSMUST00000132882.1**	Hyi	2.585618917	1.637535361	19.80162095	8.59E-06	0.003326038
**ENSMUST00000040128.11**	Atp8b4	2.417728374	2.038192274	20.24541723	8.85E-06	0.003407539
**ENSMUST00000035672.4**	Ppl	2.60080229	1.597221282	19.6156583	9.47E-06	0.003626536
**ENSMUST00000233692.1**	Lrrc73	2.578944247	1.6071816	19.47485064	1.02E-05	0.003872694
**ENSMUST00000050020.7**	Jaml	2.252198496	2.089603582	19.1849666	1.19E-05	0.004459996
**ENSMUST00000089510.4**	Cenpb	0.691954558	8.828774413	19.12108393	1.23E-05	0.004575567
**ENSMUST00000139210.7**	Socs2	2.851757636	1.221918022	18.96721204	1.33E-05	0.004908384
**ENSMUST00000050234.3**	Jrk	1.078053278	5.135798253	18.95126244	1.34E-05	0.004924073
**ENSMUST00000127984.8**	Cbfa2t3	0.691218048	8.775555733	18.91562972	1.37E-05	0.005004006
**ENSMUST00000173127.2**	Gm9574	2.090222372	2.324130758	18.82405284	1.43E-05	0.005183463
**ENSMUST00000111881.3**	Nfkb2	1.812067868	2.871430599	18.70725661	1.52E-05	0.005496874
**ENSMUST00000180252.2**	Tmem151b	2.902782205	1.092301247	18.5867386	1.62E-05	0.005826049
**ENSMUST00000023487.4**	Arhgap31	1.011545585	5.439308958	18.44933609	1.75E-05	0.006158626
**ENSMUST00000172910.2**	Crybg3	1.05867018	5.158532199	18.40626317	1.78E-05	0.006278167
**ENSMUST00000113658.7**	Gfpt1	1.043398623	5.24004564	18.38379541	1.81E-05	0.006336984
**ENSMUST00000190196.4**	Prob1	1.596870647	3.300553402	18.32104184	1.87E-05	0.006469501
**ENSMUST00000105617.7**	Ipcef1	0.864975741	6.386381639	18.29696272	1.89E-05	0.006529436
**ENSMUST00000099858.3**	Prep	0.658705716	9.17719194	18.2942255	1.89E-05	0.006529436
**ENSMUST00000040971.13**	Capn5	0.965678577	5.691155889	18.26944101	1.92E-05	0.006598963
**ENSMUST00000149137.2**	Gm13270	2.675173489	1.389537889	18.17959909	2.01E-05	0.00688442
**ENSMUST00000044195.5**	Tmc7	0.972133477	5.633514391	18.17140072	2.02E-05	0.006897539
**ENSMUST00000135229.7**	Dgcr2	1.443464753	3.684723069	18.13202921	2.06E-05	0.007024777
**ENSMUST00000082152.4**	Ube2o	1.238773818	4.283131946	18.00507573	2.2E-05	0.007447531
**ENSMUST00000050511.6**	Kynu	2.18494778	2.078697419	18.0026277	2.21E-05	0.007447531
**ENSMUST00000004565.14**	Ralb	1.261448967	4.195447806	17.86523433	2.37E-05	0.007929837
**ENSMUST00000180430.1**	Ksr2	2.687576574	1.31561539	17.82193914	2.43E-05	0.008074414
**ENSMUST00000024779.14**	Usp49	0.921717753	5.877910248	17.66871917	2.63E-05	0.008610777
**ENSMUST00000165559.2**	Ctif	2.102857236	2.200264373	17.60586527	2.72E-05	0.008870608
**ENSMUST00000055475.8**	Gpr18	0.71191288	8.061417842	17.60409652	2.72E-05	0.008870608
**ENSMUST00000204498.1**	Gm44280	2.10551515	2.15921874	17.52632093	2.83E-05	0.009216716
**ENSMUST00000024708.5**	Tnfrsf21	1.584030434	3.229964413	17.37891121	3.06E-05	0.009892078
**ENSMUST00000033915.8**	Gpm6a	2.631341363	1.334824432	17.16215365	3.44E-05	0.011019398
**ENSMUST00000021028.4**	Itgb3	1.052570974	4.979349472	17.14037813	3.47E-05	0.011089499
**ENSMUST00000043400.7**	Asprv1	2.309018389	1.791814166	17.09104218	3.67E-05	0.011591079
**ENSMUST00000208023.1**	Slc35a2	2.259435725	1.824266541	17.00877201	3.72E-05	0.011727463
**ENSMUST00000049787.2**	Lrrn4	1.633936313	3.07014468	16.99350495	3.75E-05	0.011783735
**ENSMUST00000141708.1**	Plxdc1	2.818761143	1.055704752	16.95217177	3.83E-05	0.011950139
**ENSMUST00000023105.4**	Endou	0.787140835	6.800767368	16.81315244	4.13E-05	0.012664233
**ENSMUST00000029875.3**	Pip4p2	1.332829542	3.840615409	16.72690092	4.32E-05	0.013139886
**ENSMUST00000037099.8**	Clic4	0.643789061	8.882647916	16.65300859	4.49E-05	0.013574763
**ENSMUST00000027752.14**	Lamc1	0.776612345	6.874243376	16.61247848	4.59E-05	0.013780172
**ENSMUST00000057561.8**	Wwc2	1.036929522	4.968346886	16.51719599	4.82E-05	0.014399082
**ENSMUST00000045281.12**	Smg6	0.833354923	6.301554491	16.49661935	4.87E-05	0.014495414
**ENSMUST00000091288.12**	Prnp	1.667251143	2.917886515	16.39102658	5.16E-05	0.015235528
**ENSMUST00000030949.3**	Tas1r3	1.222001363	4.147078432	16.33049231	5.32E-05	0.015628693

**ENSMUST00000029078.8**	Car2	0.998261627	5.158079653	16.33032816	5.32E-05	0.015628693
**ENSMUST00000210581.1**	Gm45353	1.605477738	3.045566074	16.09415714	6.03E-05	0.017487316
**ENSMUST00000028179.14**	Fcnb	1.946885528	2.322166731	16.07037795	6.25E-05	0.017998063
**ENSMUST00000231927.1**	Rnaset2a	0.915389624	5.625877892	16.01204011	6.29E-05	0.018078259
**ENSMUST00000134982.7**	Gm14296	2.488825159	1.326195281	15.95959731	6.48E-05	0.018525725
**ENSMUST00000234364.1**	1810073O08Rik	1.838884233	2.505484297	15.92594077	6.59E-05	0.018805713
**ENSMUST00000178993.2**	Gm4617	0.621350107	9.037674858	15.88756742	6.72E-05	0.01915249
**ENSMUST00000094897.4**	Dnaaf3	2.194825466	1.820541701	15.86551964	6.8E-05	0.019299718
**ENSMUST00000132092.1**	1110051M20Rik	2.140659711	1.926134182	15.85193673	6.85E-05	0.019390703
**ENSMUST00000199915.1**	Gm43737	0.959646607	5.304041997	15.84909436	6.86E-05	0.019390703
**ENSMUST00000081649.9**	Ifitm2	1.675778866	2.842725493	15.79724437	7.05E-05	0.019819627
**ENSMUST00000034983.6**	Atp1b3	0.616451805	9.068331327	15.70587445	7.41E-05	0.020660522
**ENSMUST00000045487.3**	Rhou	2.175048853	2.104316282	16.1961656	7.6E-05	0.021097427
**ENSMUST00000090246.4**	Sgms2	2.375894668	1.583909448	15.85498177	7.62E-05	0.021131314
**ENSMUST00000183583.7**	Ntng2	2.713081499	1.010883844	15.50033431	8.25E-05	0.022513142
**ENSMUST00000022304.9**	Thrb	2.465009772	1.332511772	15.48328466	8.32E-05	0.022673688
**ENSMUST00000028593.10**	Prrg4	1.623305896	2.919716397	15.45181677	8.46E-05	0.02296651
**ENSMUST00000160523.7**	Vamp1	1.381188592	3.541026352	15.41915374	8.61E-05	0.023322464
**ENSMUST00000212475.1**	Gm7807	2.354005543	1.500624243	15.34909554	8.94E-05	0.024157467
**ENSMUST00000170998.8**	Scn2b	1.489581799	3.331547338	15.4954795	9.07E-05	0.024477231
**ENSMUST00000144519.1**	Gps1	2.190738575	1.729844653	15.29938823	9.18E-05	0.024668887
**ENSMUST00000060148.5**	Hivep1	0.627201462	8.653686716	15.21455299	9.6E-05	0.025648846
**ENSMUST00000211473.1**	Gm9347	1.07377183	4.506600577	15.01030004	0.000106938	0.028313904
**ENSMUST00000064187.11**	Thra	1.46464195	3.245568694	14.93263581	0.000111431	0.029394289
**ENSMUST00000056977.13**	Runx3	0.904869666	5.476641029	14.90572358	0.000113032	0.029712636
**ENSMUST00000181211.1**	Gm17552	1.245442491	3.877798069	14.90655133	0.000113264	0.029712636
**ENSMUST00000029158.3**	Aar2	2.141757873	1.767565526	14.88871784	0.000114056	0.029865364
**ENSMUST00000094303.5**	Fcrl6	1.573454053	2.967723721	14.86673159	0.000115393	0.030049776
**ENSMUST00000198190.4**	Rnf216	2.1680711	1.738065333	14.84510431	0.000116723	0.030212319
**ENSMUST00000156997.7**	Dvl1	2.123140792	1.823795163	14.84503948	0.000116727	0.030212319
**ENSMUST00000098799.4**	Ehd2	0.591317189	9.243669189	14.84331481	0.000116865	0.030212319
**ENSMUST00000038053.13**	Lpp	0.817505107	6.091819532	14.83191869	0.000117542	0.030253495
**ENSMUST00000052457.14**	Mtss2	1.960211438	2.117583032	14.79164572	0.000120079	0.030693586
**ENSMUST00000165774.7**	Gbp2	1.119464739	4.302242568	14.78943983	0.00012022	0.030693586
**ENSMUST00000046206.4**	Rprd1a	0.740099793	6.758436388	14.70886236	0.000125469	0.031858334
**ENSMUST00000231568.1**	Ermard	2.613916207	1.065845372	14.68957757	0.000126759	0.032014705
**ENSMUST00000138611.7**	Ino80dos	1.464950471	3.210541081	14.66560241	0.000128381	0.032309915
**ENSMUST00000058524.2**	Zc3hav1l	1.191379259	4.019837718	14.61840745	0.000131637	0.032978986
**ENSMUST00000105818.7**	Kif17	2.290716976	1.498257089	14.61700719	0.000131734	0.032978986
**ENSMUST00000190826.1**	Ly6m	1.59735376	2.861653911	14.46330722	0.000142931	0.035554569
**ENSMUST00000087867.5**	Uprt	1.090461689	4.368119862	14.44429954	0.000144381	0.035767856
**ENSMUST00000098513.5**	Plekhf1	1.96504943	2.049646196	14.4279329	0.000145641	0.035958993
**ENSMUST00000049931.5**	Spn	0.678072467	7.511650538	14.32202139	0.000154121	0.037892903
**ENSMUST00000212276.1**	2900026A02Rik	2.519530348	1.106689538	14.31217287	0.000154876	0.037971555
**ENSMUST00000073109.11**	Ctdspl	1.492178379	3.093966786	14.30662304	0.000155411	0.03803725
**ENSMUST00000052678.8**	Flnb	0.806014717	6.064554135	14.27437444	0.000158017	0.038586161
**ENSMUST00000044492.9**	Akap9	0.760705726	6.422783521	14.2357382	0.000161295	0.039207817
**ENSMUST00000120711.1**	Gm1848	0.760785311	6.414004482	14.20654002	0.000163817	0.039564816
**ENSMUST00000178353.1**	Gm21992	1.498680905	3.067317817	14.20554851	0.000163903	0.039564816
**ENSMUST00000026565.6**	Ifitm3	1.375575329	3.426334712	14.2246013	0.00016576	0.039750707
**ENSMUST00000192833.1**	(None) lncRNA	0.701764191	7.061849304	14.03283519	0.000179664	0.042583006
**ENSMUST00000020350.14**	Lgr5	0.756865418	6.39686742	13.98456814	0.000184372	0.043462699
**ENSMUST00000208484.1**	D7Bwg0826e	1.940997967	2.034448473	13.976648	0.000185113	0.043462699
**ENSMUST00000062211.3**	Gpat2	2.272488105	1.443188796	13.97570754	0.000185206	0.043462699
**ENSMUST00000051803.7**	Aldh3b1	0.912361699	5.236743549	13.95996895	0.000186763	0.043684131
**ENSMUST00000058154.14**	Tmtc3	1.475000559	3.093075609	13.9460486	0.000188151	0.043875928
**ENSMUST00000205979.1**	Qpctl	1.751379181	2.438628057	13.94372899	0.000188383	0.043875928
**ENSMUST00000118960.1**	Car15	1.450243285	3.149629562	13.94249496	0.000188507	0.043875928
**ENSMUST00000096338.4**	Gpr152	2.382783071	1.262397684	13.86321668	0.000196628	0.045468949

**ENSMUST00000156839.1**	Ttpal	2.198145953	1.565111647	13.85379841	0.000197616	0.045623333
**ENSMUST00000059341.4**	Zc2hc1c	1.568068621	2.864413198	13.8423194	0.000198883	0.045841677
**ENSMUST00000005671.9**	Igf1r	1.049573733	4.43546616	13.83779409	0.000199349	0.045874786
**ENSMUST00000067458.6**	Sema5a	3.639412476	4.416924321	34.1782988	0.000204773	0.04689568
**ENSMUST00000112707.2**	Lrrc8b	0.770240361	6.244318037	13.7875638	0.00020531	0.046928667
**ENSMUST00000003561.9**	Phyhip	2.096810671	1.721291854	13.77959715	0.000205576	0.046928667
**ENSMUST00000135091.1**	Mtln	1.405864455	3.247832623	13.75955962	0.000207781	0.047280357
**ENSMUST00000053078.4**	Map10	1.113478994	4.180729456	13.75524674	0.000208258	0.047313452
**ENSMUST00000231574.1**	Gm2792	0.953075215	4.927914037	13.72149338	0.000212034	0.048009217
**ENSMUST00000053686.8**	Uck2	0.790459387	6.064749939	13.71885993	0.000212332	0.048009217
**ENSMUST00000119848.7**	Eme2	0.944359867	4.972499349	13.70129894	0.000214326	0.04830686
**ENSMUST00000119978.1**	Gm12671	0.638325412	7.902025085	13.66813018	0.000218145	0.049089967

Downregulated in *Dido1*ΔE16

TranscriptID	Symbol	logFC	logCPM	F	PValue	FDR
**ENSMUST00000087517.9**	Dido1	-6.321931699	6.127988892	360.3444673	2.51E-80	3.57E-75
**ENSMUST00000087543.4**	B3gatl4	-3.661303587	4.557737119	102.7008546	3.92E-24	1.86E-20
**ENSMUST00000060185.2**	Fndc9	-1.953593451	6.973307638	83.07163692	7.94E-20	2.36E-16
**ENSMUST00000038552.12**	Coro7	-1.869896301	6.500700051	68.86100539	1.06E-16	2.32E-13
**ENSMUST00000033056.4**	Pycard	-1.883482372	5.753463343	54.41246882	1.63E-13	2.79E-10
**ENSMUST00000222616.1**	Gm19951	-4.80983627	3.183063661	56.41428902	5.84E-12	8.07E-09
**ENSMUST00000103510.1**	Ighv-26	-1.56806827	6.193893519	45.3781159	1.63E-11	2.07E-08
**ENSMUST00000154177.1**	Gm12678	-1.655462674	5.889877298	45.31532015	1.68E-11	2.1E-08
**ENSMUST00000135559.7**	Iffo1	-4.15081223	2.8172429	45.28881682	1.7E-11	2.11E-08
**ENSMUST00000112751.1**	Bcl2	-2.270625247	4.416905344	44.30505812	5.2E-11	6.07E-08
**ENSMUST00000232755.1**	Brwd1	-1.388856764	6.525235209	40.566014	1.9E-10	2.07E-07
**ENSMUST00000124462.2**	Arhgap27os3	-1.889261018	4.729862382	37.95072549	7.32E-10	6.99E-07
**ENSMUST00000025786.8**	Pacs1	-1.692871768	5.242494037	37.59600881	8.9E-10	8.18E-07
**ENSMUST00000103328.2**	Igkv10-96	-1.692240036	5.090174833	35.70522519	2.3E-09	1.99E-06
**ENSMUST00000048935.5**	DMrt3	-2.045531931	4.255897118	34.85501199	3.59E-09	2.96E-06
**ENSMUST00000102600.3**	Fndc5	-1.837701248	4.602258186	34.4097588	4.47E-09	3.62E-06
**ENSMUST00000159080.5**	Clec2i	-7.983593355	1.558483814	33.37174352	7.62E-09	6.03E-06
**ENSMUST00000139071.7**	Iffo1	-7.890355654	1.463687259	31.36565171	2.14E-08	1.55E-05
**ENSMUST00000138127.7**	Zfp318	-4.442870304	1.956464378	29.51947886	5.54E-08	3.8E-05
**ENSMUST00000144920.3**	Cmas	-2.003662183	3.954036653	29.30672633	6.31E-08	4.28E-05
**ENSMUST00000139806.1**	Casz1	-2.62217115	3.113146752	28.85298785	7.81E-08	5.23E-05
**ENSMUST00000159745.1**	Pip4p1	-1.716446429	4.448499287	28.85859076	7.82E-08	5.23E-05
**ENSMUST00000108809.7**	Trim11	-1.328841978	6.363271883	31.2363446	8.09E-08	5.36E-05
**ENSMUST00000076957.6**	Zdhhc8	-1.179685888	6.382338265	28.64868403	8.68E-08	5.72E-05
**ENSMUST00000130486.1**	Gm15675	-1.339694373	5.647834982	28.47516073	9.58E-08	6.23E-05
**ENSMUST00000117906.1**	Gm14127	-1.420073761	5.320221421	28.24872281	1.07E-07	6.91E-05
**ENSMUST00000103507.1**	Ighv1-22	-1.800719921	4.202435221	27.47968675	1.59E-07	9.84E-05
**ENSMUST00000103495.2**	Ighv10-3	-1.495800386	4.954604049	27.25163869	1.79E-07	0.000110187
**ENSMUST00000103544.2**	Ighv1-75	-1.464349621	5.054374643	27.18513212	1.85E-07	0.000113064
**ENSMUST00000192591.1**	Ighv8-8	-1.398220873	5.268371238	26.98035366	2.06E-07	0.000123576
**ENSMUST00000201831.3**	Gm20559	-1.182468775	6.205358653	26.98167247	2.22E-07	0.000131625
**ENSMUST00000052124.8**	Nlrc4	-1.870336605	4.02341972	26.68329429	2.4E-07	0.00014055
**ENSMUST00000103526.2**	Ighv1-55	-1.310757938	5.559107453	26.45566013	2.7E-07	0.000156834
**ENSMUST00000026408.6**	Gdf11	-1.261248622	5.657364824	25.54015348	4.34E-07	0.000241657
**ENSMUST00000001327.10**	Itgb7	-2.61163875	3.107993862	26.35748488	4.57E-07	0.000252715
**ENSMUST00000219633.1**	Icosl	-7.525804932	1.161075383	25.31362351	4.9E-07	0.000267191
**ENSMUST00000057725.9**	Samhd1	-0.792015264	9.539454369	24.98671412	5.77E-07	0.000310391
**ENSMUST00000171330.6**	Slamf6	-1.13563657	6.058165089	24.0025037	9.62E-07	0.000493113
**ENSMUST00000124485.7**	Fam129c	-1.066469298	6.676448923	24.608023	9.92E-07	0.000504511
**ENSMUST00000030417.9**	Cdc42	-1.590191046	4.399796876	23.90182956	1.21E-06	0.000602967
**ENSMUST00000035218.8**	Nckipsd	-1.283554478	5.317747107	23.40961893	1.31E-06	0.000645474
**ENSMUST00000136582.1**	Dpm1	-1.543326311	4.409283137	23.36333687	1.34E-06	0.000656648
**ENSMUST00000103515.1**	Ighv1-39	-1.547760676	4.378250288	23.00184139	1.62E-06	0.000781701

**ENSMUST00000029569.8**	Slc35a3	-1.230029896	5.472174724	22.83947362	1.76E-06	0.000841061
**ENSMUST00000224965.1**	Blk	-1.250333269	5.365670422	22.82565724	1.78E-06	0.000845437
**ENSMUST00000108846.1**	Galnt10	-2.251345495	3.090909269	22.57263474	2.03E-06	0.000942717
**ENSMUST00000103386.2**	Igkv6-23	-1.356995082	4.870950986	22.1892887	2.47E-06	0.001128871
**ENSMUST00000127842.7**	Ctsh	-3.440887594	1.979518078	22.14112208	2.54E-06	0.001150331
**ENSMUST00000177669.1**	Pfn1	-1.038391608	6.305071193	22.02354857	2.7E-06	0.001218565
**ENSMUST00000054279.14**	Sp100	-1.679650835	4.002008163	21.97562283	2.76E-06	0.001241119
**ENSMUST00000028755.7**	Ehd4	-0.852689493	7.956604028	21.84851254	2.95E-06	0.001313675
**ENSMUST00000094646.5**	Vps4b	-0.9528883	6.769731742	21.2618847	4.01E-06	0.001683743
**ENSMUST00000110031.3**	Auh	-1.699163386	3.82382454	20.55017493	5.81E-06	0.002357878
**ENSMUST00000229230.1**	Gm18724	-1.248267124	5.065038697	20.40789211	6.26E-06	0.002511213
**ENSMUST00000098486.3**	Bcl2a1d	-3.109744396	2.036965161	20.12783914	7.27E-06	0.002883393
**ENSMUST00000119311.7**	Auh	-1.774167059	3.641515153	20.09724599	7.36E-06	0.002912898
**ENSMUST00000028205.9**	BC005624	-1.062442735	5.875275434	19.95054576	7.95E-06	0.003119156
**ENSMUST00000124563.7**	Napa	-2.896490967	2.171873297	19.93717851	8.02E-06	0.003136808
**ENSMUST00000191475.1**	Gm8369	-3.285587564	1.914526522	19.76182214	8.77E-06	0.003386816
**ENSMUST00000105520.7**	Enpp1	-2.7745023	2.294940484	19.70209371	9.05E-06	0.003475495
**ENSMUST00000236643.1**	H2-Ab1	-1.809995864	3.510553429	19.50810809	1.01E-05	0.003834755
**ENSMUST00000028928.7**	Gzf1	-1.002841441	6.123570802	19.40183321	1.06E-05	0.004012909
**ENSMUST00000089688.5**	Mmp14	-3.049193137	2.042926237	19.38315756	1.07E-05	0.004041594
**ENSMUST00000032288.5**	Klra1	-2.381443479	2.679955902	19.16790685	1.2E-05	0.004488191
**ENSMUST00000200318.1**	Reln	-1.801471893	3.508443606	19.06073033	1.27E-05	0.004699707
**ENSMUST00000194738.5**	Igha	-2.28159457	2.761208115	19.01513182	1.3E-05	0.004802556
**ENSMUST00000103521.2**	Ighv1-50	-1.339319172	4.520063	18.8886975	1.39E-05	0.005037705
**ENSMUST00000103321.2**	Igkv1-110	-1.244154495	4.83149238	18.69219071	1.54E-05	0.005531082
**ENSMUST00000033611.4**	Xkrx	-1.877057128	4.45365182	22.18038806	1.65E-05	0.005898442
**ENSMUST00000149244.7**	Lrmp	-1.320774437	4.50840102	18.5478536	1.66E-05	0.005919815
**ENSMUST00000059644.12**	Rbm33	-0.933005737	6.391954558	18.50911402	1.69E-05	0.006016918
**ENSMUST00000103330.1**	Igkv10-94	-1.581022172	3.863354835	18.47096412	1.73E-05	0.006113882
**ENSMUST00000171415.7**	Ndufb8	-1.546232139	3.937086795	18.44760915	1.75E-05	0.006158626
**ENSMUST00000190715.6**	Cep70	-2.040272535	2.979809197	17.91385147	2.31E-05	0.007766293
**ENSMUST00000185851.1**	Ms4a1	-1.574366466	3.801611177	17.7073616	2.58E-05	0.008535729
**ENSMUST00000189288.1**	F730311O21	-1.301374978	4.811238435	18.38186761	2.59E-05	0.008572595
**ENSMUST00000107802.7**	Trim59	-0.855661493	6.779539165	17.35938084	3.09E-05	0.009971639
**ENSMUST00000049872.8**	Gpr183	-1.171565745	5.567322821	18.39201523	3.19E-05	0.010252892
**ENSMUST00000123453.1**	Gmip	-2.080640912	2.83817036	17.09162441	3.56E-05	0.011327043
**ENSMUST00000074259.14**	Nrm	-0.765176205	7.74999753	17.0492499	3.64E-05	0.011556825
**ENSMUST00000077605.11**	Eif4a2	-0.857011307	6.70463286	17.09189787	3.65E-05	0.011557296
**ENSMUST00000088785.5**	Zfp566	-1.521634845	3.830695557	16.99129999	3.76E-05	0.011783735
**ENSMUST00000025266.5**	Lta	-2.498746182	2.32040539	16.90669591	3.96E-05	0.012262889
**ENSMUST00000089497.6**	Lsy1	-1.005583807	5.690951661	16.88235191	3.98E-05	0.012290273
**ENSMUST00000034466.9**	Gnpat	-1.1617632	4.901555007	16.86739962	4.01E-05	0.01236066
**ENSMUST00000143378.7**	RIKEN cDNA	-1.063285171	5.357939252	16.77561341	0.0000422	0.012867344
**ENSMUST00000014597.4**	Blk	-0.648216171	9.463687199	16.77374043	4.21E-05	0.012867344
**ENSMUST00000103369.1**	Igkv12_41	-1.843511231	3.176795449	16.64238842	4.51E-05	0.013622056
**ENSMUST00000168846.2**	Prkag1	-0.95133699	5.941714353	16.53153497	4.79E-05	0.01435078
**ENSMUST00000063140.14**	Hcrtr2	-4.031617164	1.218501868	16.50007809	4.87E-05	0.014495414
**ENSMUST00000049295.14**	Ehbp1l1	-0.986878979	5.72915136	16.49113643	4.89E-05	0.014507106
**ENSMUST00000106357.7**	Ypel3	-1.209641194	4.602325694	16.37671719	5.19E-05	0.015313957
**ENSMUST00000027266.3**	Ormdl1	-0.9487111	5.913806377	16.29367741	5.43E-05	0.015901113
**ENSMUST00000030964.5**	CD38	-0.651117231	9.203340782	16.23521185	5.6E-05	0.016365734
**ENSMUST00000103492.1**	Ighv10_1	-1.084158731	5.162109654	16.20811053	5.68E-05	0.016567508
**ENSMUST00000128652.1**	RIKEN cDNA	-1.00947143	5.618464323	16.30293479	0.0000572	0.016656537
**ENSMUST00000096243.6**	B3gat3	-1.085825454	5.11984947	16.08608819	6.05E-05	0.017500942
**ENSMUST00000153442.7**	Hnrnpdl	-1.096473772	5.059817253	16.08560917	6.06E-05	0.017500942
**ENSMUST00000074733.10**	Sept11	-0.708824	8.200756884	16.06479354	6.12E-05	0.017652724
**ENSMUST00000103493.2**	Ighv1_4	-1.602674675	3.567852863	15.9900552	6.37E-05	0.01825261
**ENSMUST00000015460.4**	Slamf1	-2.286564693	2.455558045	15.87475288	6.78E-05	0.019273272
**ENSMUST00000103534.1**	Ighv1-63	-1.756876208	3.266879808	15.83304593	6.93E-05	0.019498865

**ENSMUST00000120267.8**	Atg16l2	-0.927330632	5.944811642	15.76609552	7.17E-05	0.020061185
**ENSMUST00000161705.2**	Mcoln1	-1.22439226	4.453059577	15.67899419	7.51E-05	0.020896092
**ENSMUST00000173103.1**	H2-Ab1	-1.822872128	3.052427033	15.58433112	7.92E-05	0.021770549
**ENSMUST00000070720.7**	Sorcs2	-1.316907882	4.181814742	15.51468541	8.19E-05	0.022385759
**ENSMUST00000219038.1**	Icosl	-3.030945151	1.624124969	15.22695931	9.53E-05	0.025528804
**ENSMUST00000164035.7**	Arid3b	-1.747583514	3.159753895	15.22848471	9.53E-05	0.025528804
**ENSMUST00000140896.1**	Coro1a	-1.402439401	3.905593784	15.2313424	9.66E-05	0.025761121
**ENSMUST00000103548.2**	Ighv1_81	-1.022871062	5.272466495	15.10425623	0.000101745	0.027039536
**ENSMUST00000037913.8**	Rmi2	-0.853572767	6.324823147	15.08240218	0.000106569	0.028268881
**ENSMUST00000195325.1**	Ighv1_2	-1.506154793	3.650215269	14.99575642	0.000107765	0.028480012
**ENSMUST00000123602.1**	Dctn5	-1.632783615	3.336595336	14.90422859	0.000113244	0.029712636
**ENSMUST00000200926.1**	Nabp2l1	-2.27234845	2.326272308	14.88002122	0.000114583	0.029948313
**ENSMUST00000117892.1**	Slc48a1	-0.771435071	6.987704437	14.85486262	0.000116121	0.030184274
**ENSMUST00000035842.6**	Rassf1	-0.654480122	8.580568303	14.80443381	0.000119268	0.030611202
**ENSMUST00000204885.1**	Hnrnpa2b1	-0.994640097	5.339542027	14.72942994	0.000124107	0.031625416
**ENSMUST00000113481.8**	Zfp318	-0.799788028	6.608211956	14.71730276	0.000125115	0.031825347
**ENSMUST00000103384.1**	Igkv8_24	-1.313846169	4.059313433	14.53626051	0.000137502	0.034362495
**ENSMUST00000207327.1**	Fcer2a	-3.283156331	1.372850445	14.44130764	0.000145658	0.035958993
**ENSMUST00000072729.9**	Ms4a4c	-2.331771874	2.251738081	14.33233054	0.000158196	0.038586161
**ENSMUST00000119728.1**	Npm3-ps1	-0.924176794	5.638522107	14.15437794	0.000168422	0.040274585
**ENSMUST00000001455.12**	Mef2d	-0.595348928	9.43343979	14.15338483	0.000168511	0.040274585
**ENSMUST00000172057.7**	Ralgps2	-0.67314841	8.049282892	14.1192891	0.000171592	0.040942466
**ENSMUST00000186927.1**	Ly6e	-1.186612293	4.339905695	13.99950832	0.000182937	0.04321498
**ENSMUST00000192106.1**	Gm8146	-1.425706995	3.686588118	13.98002222	0.000184781	0.043462699
**ENSMUST00000150208.7**	Napa	-1.487700985	3.537511902	13.9375477	0.000190788	0.044299427
