# Supplementary material for: The chromatin reader Dido3 is a regulator of the gene network that controls B cell differentiation
Source: Cell Biosci. 2025 Apr 26;15:56. doi: 10.1186/s13578-025-01394-x (PMC12034202; doi:10.1186/s13578-025-01394-x)
Supplement: Supplementary file 5 — Additional file5 (PDF 236 KB) [file 13578_2025_1394_MOESM5_ESM.pdf]

Supplementary Table 3

Differentially expressed Ighv (variable) regions in pre-B cells (RNA-seq data). Lists of up- and down-regulated fragmentsof *Igh, Igl* and *Igk* genes in Dido3-deficient pre-B cells in comparation to control WT, ordered by false discovery rate (FDR). Log2 fold-change of expression between conditions being tested (logFC), log2 of counts per million, the average taken over all libraries (logCPM), likelihood ratio statistics. (LR) and statistical significance as p-value and false discovery rate using Benjamini-Hochberg correction (FDR) are included.

Ensembl Gene ID_Gene symbol)	logFC	logCPM	LR	PValue	FDR
ENSMUSG00000076613_Ighg2b	5.177375763	11.0114599	201.453585	1.01E-45	2.80E-43
ENSMUSG00000094028_Ighd4-1	3.435788362	11.6479589	114.786492	8.76E-27	1.22E-24
ENSMUSG00000094057_Ighd2-7	4.13279968	8.67285432	92.4153468	7.03E-22	6.51E-20
ENSMUSG00000095897_Ighd2-5	2.705912053	9.02573405	60.6345883	6.87E-15	4.78E-13
ENSMUSG00000095444_Ighd2-4	2.203923512	9.67438993	54.5985616	1.48E-13	8.22E-12
ENSMUSG00000076630_Ighd1-1	1.793919186	11.253159	30.6397651	3.11E-08	1.44E-06
ENSMUSG00000096568_Ighd2-3	1.798574118	9.51005007	29.510394	5.56E-08	2.21E-06
ENSMUSG00000096150_Ighv1-85	2.001683257	8.37291067	21.7637665	3.08E-06	0.00010716
ENSMUSG00000093955_Ighv1-34	-1.683806396	8.63910452	20.0707275	7.46E-06	0.00023052
ENSMUSG00000076633_Ighv5-2	1.149102373	10.7760075	18.7699476	1.47E-05	0.00040997
ENSMUSG00000096464_Ighv2-2	1.196086747	11.7479314	17.8036236	2.45E-05	0.00061897
ENSMUSG00000094117_Igkv3-12	1.177994346	11.2784643	16.1877401	5.74E-05	0.00132894
ENSMUSG00000095416_Ighv1-12	-1.180049493	9.75657498	15.9939	6.35E-05	0.00135893
ENSMUSG00000103439_Gm7019	1.111729657	12.0007745	15.0978134	0.00010208	0.00202705
ENSMUSG00000095130_Ighv1-39	-1.073056122	10.0470355	14.7446491	0.0001231	0.00228139
ENSMUSG00000105630_Gm42543	1.958377857	7.62244402	13.8997266	0.00019283	0.00331427
ENSMUSG00000094051_Ighv1-36	-1.729868909	8.40680503	13.7040139	0.000214	0.00331427
ENSMUSG00000076620_Ighj2	1.173126641	9.80407887	13.6987894	0.00021459	0.00331427
ENSMUSG00000094561_Ighv1-22	-0.968802278	10.0309777	11.9406639	0.00054922	0.00803594
ENSMUSG00000076531_Igkv4-92	0.947768886	9.64418052	11.4133208	0.00072919	0.01013579
ENSMUSG00000094940_Ighv1-84	-1.569747231	7.86347906	11.0600177	0.00088209	0.01167723
ENSMUSG00000076598_Igkv3-7	0.974039758	11.8250008	10.7807443	0.00102561	0.01296004
ENSMUSG00000096074_Ighv1-72	1.078454326	9.63555533	10.0677939	0.00150884	0.01823723
ENSMUSG00000095866_Ighv2-4	0.955057938	9.2066517	9.66627868	0.00187681	0.02173973
ENSMUSG00000096649_Ighv1-31	-1.603278868	7.74617826	9.53362458	0.00201741	0.02243363
ENSMUSG00000094652_Ighv1-42	-0.975189499	9.26275221	9.32514755	0.0022603	0.02379573
ENSMUSG00000076695_Ighv1-18	-0.92584829	10.3922373	9.2754911	0.0023224	0.02379573
ENSMUSG00000095863_Ighv1-67	-1.144229293	8.51612379	9.21783174	0.00239669	0.02379573
ENSMUSG00000093896_Ighv1-76	-0.834787482	10.7236013	8.36705245	0.00382085	0.03662744
ENSMUSG00000094546_Ighv1-26	-0.911194794	11.9062431	8.2095574	0.00416703	0.03857043
ENSMUSG00000106124_Gm42539	1.869388279	6.46688357	8.15214777	0.00430102	0.03857043
ENSMUSG00000103537_Gm37838	1.567022347	6.59522279	7.97100381	0.00475326	0.04129392
ENSMUSG00000094694_Ighv1-9	-0.759644961	11.0040389	7.40626887	0.0064997	0.05475503
ENSMUSG00000094951_Ighv5-6	0.726667156	11.0872131	7.30006217	0.00689522	0.05490461
ENSMUSG00000095700_Ighv10-3	-0.783609818	10.5656279	7.29557865	0.00691245	0.05490461
ENSMUSG00000095612_Ighv5-4	0.74439873	11.5734534	7.11169352	0.00765827	0.05913888
ENSMUSG00000000001_Gm30996	2.102964555	4.81116565	6.75350437	0.00935637	0.07029924
ENSMUSG00000103297_Ighv3-2	-1.472817484	7.33039719	6.6933679	0.00967722	0.07079652
ENSMUSG00000104760_Igkv3-8	1.658945266	5.00512581	6.47040267	0.01096857	0.0781862
ENSMUSG00000102942_Ighv1-33	-2.126420546	3.91093715	6.39753437	0.0114279	0.07942388
ENSMUSG00000094533_Ighv11-1	-1.669326819	5.8759403	6.31963143	0.01194084	0.08005876
ENSMUSG00000076596_Igkv3-10	0.673171372	10.4226837	6.29685931	0.01209521	0.08005876
ENSMUSG00000106098_Igkv14-118-2	1.495531483	5.31228066	6.05846689	0.01383978	0.08947577
ENSMUSG00000076501_Igkv2-137	0.646926589	10.8553969	5.99636998	0.01433534	0.08980706
ENSMUSG00000095197_Ighv1-59	-0.721533304	9.91442933	5.94771485	0.01473638	0.08980706

ENSMUSG00000102654_Ighv1-21	-1.201134567	7.45384167	5.93297185	0.01486016	0.08980706
ENSMUSG00000094087_Ighv1-61	-0.759887613	9.16421927	5.71295115	0.01684021	0.09960803
ENSMUSG00000094164_Ighv2-3	0.685587426	9.60526463	5.34609232	0.02076877	0.12028578
ENSMUSG00000103168_Gm30948	2.033469731	3.00730278	5.22282817	0.02229227	0.12408686
ENSMUSG00000106601_Ighv1-70	-1.381132688	5.69079483	5.22083902	0.02231778	0.12408686
ENSMUSG00000096594_Igkv8-19	0.632206719	11.1831626	5.11593087	0.02370714	0.12805691
ENSMUSG00000095170_Ighv8-11	-1.36949941	6.25424398	5.09802581	0.02395309	0.12805691
ENSMUSG00000094075_Ighv1-80	-0.620116103	10.0480177	4.95710873	0.02598362	0.13629142
ENSMUSG00000095981_Ighv10-1	-0.588829595	10.554939	4.81117145	0.02827582	0.1455681
ENSMUSG00000102888_Ighv1-11	-0.92138606	8.27124633	4.77202889	0.02892572	0.14620636
ENSMUSG00000095442_Ighv1-4	-0.663938949	9.15982116	4.71780225	0.02985187	0.14819323
ENSMUSG00000076589_Igkv8-18	0.947540018	8.36825993	4.62057115	0.03159072	0.15407403
ENSMUSG00000102952_Ighv1-25	-1.307576367	5.74547552	4.29499462	0.03822472	0.18321502
ENSMUSG00000095771_Igkv14-111	0.533274469	10.2864999	4.17979124	0.04090874	0.19275643
ENSMUSG00000105462_Igkv4-77	0.531311477	9.93846955	4.13771285	0.0419378	0.19431179
ENSMUSG00000076676_Ighv12-3	-0.687180345	9.13269173	4.10008879	0.04288096	0.19542471
ENSMUSG00000094198_Ighv1-50	-0.585001217	10.1161914	4.01208031	0.04517538	0.2005046
ENSMUSG00000094689_Ighv1-81	-0.584217099	10.9978384	3.99755463	0.04556633	0.2005046
ENSMUSG00000076621_Ighj1	0.884264822	14.4042261	3.97577047	0.04615933	0.2005046
ENSMUSG00000095589_Ighv1-55	-0.555040787	10.9645996	3.94440051	0.04702761	0.20113346
ENSMUSG00000094552_Ighd3-2	0.773590698	8.43986094	3.91042688	0.04798741	0.20212877
ENSMUSG00000096672_Ighv1-63	-0.763362468	8.82568421	3.76300446	0.05239835	0.21741404
ENSMUSG00000106599_Ighv1-73	-1.398410132	4.48734055	3.71656662	0.05387505	0.22025386
ENSMUSG00000102301_Ighv8-2	-0.936167637	7.69514926	3.5994213	0.05779969	0.23287411
ENSMUSG00000104213_Ighd	-0.659177666	9.88175883	3.47103318	0.06245229	0.24647816
ENSMUSG00000104769_Igkv8-34	0.941357387	7.02804306	3.443371	0.063506	0.24647816
ENSMUSG00000104452_Ighv8-8	-0.527043772	11.3098304	3.43480628	0.06383607	0.24647816
ENSMUSG00000095429_Ighv5-12	0.49803958	10.6063664	3.34159822	0.06754897	0.25724128
ENSMUSG00000076680_Ighv6-6	-0.527580202	9.7000838	3.21736697	0.07286074	0.27067816
ENSMUSG00000073028_Igkv4-71	-0.679573852	8.77448633	3.18170461	0.07446701	0.27067816
ENSMUSG00000076581_Igkv8-26	1.030033169	6.3041543	3.16804027	0.0750925	0.27067816
ENSMUSG00000096250_Ighd2-6	-0.587084644	10.4542299	3.10255832	0.07816936	0.27067816
ENSMUSG00000076606_Igkj3	0.640924259	11.5116641	3.09446147	0.07855914	0.27067816
ENSMUSG00000095127_Ighv1-82	-0.504245407	11.5938878	3.08994271	0.07877758	0.27067816
ENSMUSG00000102678_Ighv1-21-1	-1.004043452	5.873329	3.08886053	0.07882999	0.27067816
ENSMUSG00000094787_Ighv1-54	-0.5071661	9.60429213	3.08810393	0.07886666	0.27067816
ENSMUSG00000094420_Igkv10-96	-0.453739549	10.5607213	3.06213137	0.08013649	0.27168225
ENSMUSG00000095571_Ighv5-17	0.463357616	10.6474763	2.91762159	0.08761692	0.29001116
ENSMUSG00000094262_Igkv4-62	0.874528166	7.16048478	2.9054264	0.0882819	0.29001116
ENSMUSG00000106403_Igkv20-101-2	-1.082974466	6.07234901	2.88025985	0.08967155	0.29001116
ENSMUSG00000095761_Ighv1-20	-1.055689119	7.01745686	2.8776134	0.08981905	0.29001116
ENSMUSG00000103203_Gm37327	1.326136828	5.73368861	2.85504232	0.09108784	0.29001116
ENSMUSG00000103271_Ighv4-2	-0.806116273	7.60655197	2.80778994	0.09380753	0.29001116
ENSMUSG00000096078_Ighv1-62-2	1.001763931	7.45787547	2.80751714	0.09382348	0.29001116
ENSMUSG00000095794_Igkv6-17	0.429882915	10.4922438	2.79456216	0.0945846	0.29001116
ENSMUSG00000094134_Ighv5-15	0.492452966	9.2415781	2.78869158	0.09493171	0.29001116
ENSMUSG00000076731_Ighv8-12	-0.505490423	10.6615352	2.76956572	0.09607222	0.2903052
ENSMUSG00000076666_Ighv14-4	-0.439885409	10.9026793	2.64081998	0.10414978	0.31132944
ENSMUSG00000096844_Igkv6-14	0.598552395	8.88294692	2.58305221	0.10801321	0.31944331
ENSMUSG00000095630_Igkv6-23	-0.436742903	9.76588655	2.49506662	0.11420354	0.33403597
ENSMUSG00000094502_Ighv1-69	-0.406074466	10.2378681	2.47934131	0.11535055	0.33403597

ENSMUSG00000103873_Ighv1-38	-0.847369758	7.1242255	2.44680215	0.11776457	0.33688302
ENSMUSG00000104103_Gm9517	0.568057076	8.59524348	2.43363375	0.11875732	0.33688302
ENSMUSG00000093838_Ighv3-1	-0.524645867	8.89825088	2.36655136	0.12396063	0.34809146
ENSMUSG00000094094_Igkv5-45	0.397046687	10.0367108	2.30828563	0.12868591	0.35774684
ENSMUSG00000094345_Igkv14-126	-0.501278722	9.03501953	2.27166813	0.13175802	0.36266068
ENSMUSG00000096499_Ighv1-5	-0.41639089	11.0010588	2.2420177	0.13430563	0.36604867
ENSMUSG00000105906_Iglc1	0.697952416	15.6446864	2.2132972	0.13682596	0.36929724
ENSMUSG00000103989_Ighv5-21	1.185740316	3.696691	2.18464116	0.13939355	0.37260968
ENSMUSG00000095335_Igkv3-5	0.404687686	9.63444883	2.11418872	0.14593937	0.38639186
ENSMUSG00000095285_Ighv5-9	0.490247096	8.82247985	2.09121541	0.14814817	0.3869217
ENSMUSG00000076672_Ighv3-6	-0.439935206	11.6608676	2.08324695	0.1489231	0.3869217
ENSMUSG00000095204_Ighv1-52	-0.383154594	9.90330242	2.05735825	0.15147257	0.38990163
ENSMUSG00000105955_Igkv12-40	-0.831343442	7.37205575	2.01372719	0.15588195	0.39520584
ENSMUSG00000094993_Igkv4-51	0.54121124	8.50420195	2.00809557	0.15646162	0.39520584
ENSMUSG00000076543_Igkv4-74	-0.517388412	8.59620808	1.99520211	0.15779801	0.39520584
ENSMUSG00000105757_Ighv1-83	-0.840099243	5.46038142	1.97360712	0.16006551	0.39730545
ENSMUSG00000096498_Ighv2-5	0.376127475	9.99479459	1.93448397	0.16426871	0.4041301
ENSMUSG00000106668_Iglj1	0.65977613	16.2949127	1.8530564	0.17342833	0.42292173
ENSMUSG00000104712_Igkv4-75	-0.776275593	6.01635123	1.81471057	0.17794418	0.43016072
ENSMUSG00000076934_Iglv1	0.621829349	15.5288016	1.79752389	0.18001213	0.43140839
ENSMUSG00000103254_Ighv1-15	-0.420562261	9.70403143	1.6777382	0.19522526	0.46370708
ENSMUSG00000094433_Igkv5-43	0.335543925	10.727831	1.65992101	0.19761399	0.46370708
ENSMUSG00000076578_Igkv6-29	-0.702788094	7.42870741	1.65342567	0.19849332	0.46370708
ENSMUSG00000096355_Ighv8-4	-0.679160692	6.59248066	1.60774962	0.20480843	0.47447285
ENSMUSG00000095519_Ighv1-66	-0.500453107	8.57595717	1.57623443	0.20930393	0.4808801
ENSMUSG00000095889_Ighv1-58	-0.361396915	9.36345882	1.54469072	0.21392095	0.48435744
ENSMUSG00000076534_Igkv12-89	-0.410829361	8.95769102	1.54201517	0.2143181	0.48435744
ENSMUSG00000094174_Ighv6-4	0.870134735	4.21543851	1.52390026	0.21703022	0.48435744
ENSMUSG00000102765_Ighv1-62	-0.586863251	7.43619533	1.50140182	0.2204558	0.48435744
ENSMUSG00000105599_Gm9238	-0.786277568	5.27895761	1.46407662	0.2262826	0.48435744
ENSMUSG00000076940_Iglv2	0.535480051	13.8034696	1.46273654	0.22649521	0.48435744
ENSMUSG00000094319_Igkv4-54	0.346279984	9.38795044	1.45309118	0.22803259	0.48435744
ENSMUSG00000096108_Ighv11-2	-0.720556782	8.95932044	1.4509443	0.22837649	0.48435744
ENSMUSG00000076607_Igkj4	0.531183988	14.8029883	1.42745702	0.2321799	0.48435744
ENSMUSG00000093906_Igkv9-129	0.331344172	10.0123279	1.41938817	0.23350413	0.48435744
ENSMUSG00000104575_Igkv9-119	1.009895791	4.47058237	1.41429226	0.23434516	0.48435744
ENSMUSG00000076532_Igkv4-91	0.43127226	8.69238204	1.40767817	0.23544221	0.48435744
ENSMUSG00000076526_Igkv12-98	0.471417481	8.36153569	1.39581348	0.23742579	0.48435744
ENSMUSG00000076505_Igkv1-131	0.413437289	8.77375358	1.39337613	0.23783578	0.48435744
ENSMUSG00000105928_Gm9256	0.711054143	6.66474411	1.38232606	0.23970533	0.48435744
ENSMUSG00000104422_Ighv1-14	-0.92659312	3.8392428	1.38067141	0.23998681	0.48435744
ENSMUSG00000076733_Ighv8-13	-0.90725106	3.57763276	1.37803332	0.24043643	0.48435744
ENSMUSG00000076535_Igkv1-88	0.334989463	9.52801973	1.3625797	0.2430909	0.48494172
ENSMUSG00000093894_Ighv1-53	-0.331863888	10.3608133	1.35609585	0.24421525	0.48494172
ENSMUSG00000076583_Igkv8-24	-0.30269033	9.87016226	1.33342668	0.24819652	0.48882087
ENSMUSG00000094322_Ighv9-4	0.350445863	9.15944924	1.32506266	0.24968548	0.48882087
ENSMUSG00000096490_Igkv10-94	-0.364783491	9.55513238	1.2797716	0.25794151	0.49923956
ENSMUSG00000106428_Gm42667	0.596237557	6.8861137	1.27213745	0.25936608	0.49923956
ENSMUSG00000076533_Igkv4-90	-0.677187307	6.17758504	1.26657052	0.26041104	0.49923956
ENSMUSG00000096020_Ighv1-75	-0.323557453	10.70344	1.25715362	0.26219056	0.49923956
ENSMUSG00000076514_Igkv17-121	0.308582629	11.3131073	1.24171107	0.26514154	0.50142414

ENSMUSG00000094356_Igkv8-28	-0.310896373	9.53487803	1.21455404	0.27043208	0.50797377
ENSMUSG00000076550_Igkv4-63	0.347530991	8.90454381	1.12611662	0.28860519	0.53513966
ENSMUSG00000076522_Igkv16-104	0.271050448	10.2715059	1.12546645	0.28874442	0.53513966
ENSMUSG00000106039_Iglc4	0.626748129	6.10958567	1.08547482	0.29747602	0.54428012
ENSMUSG00000106372_Igkv14-126-1	0.640671539	5.10650691	1.08495374	0.29759201	0.54428012
ENSMUSG00000076580_Igkv8-27	0.315112972	10.7556421	1.06093665	0.30300165	0.55055202
ENSMUSG00000103290_Ighv1-23	-0.552986541	6.51221135	0.97457853	0.32354096	0.57933155
ENSMUSG00000106494_Igkv2-107	-0.679545597	4.49058185	0.97354072	0.32379872	0.57933155
ENSMUSG00000076652_Ighv7-3	-0.255794517	10.0408208	0.96834802	0.32509252	0.57933155
ENSMUSG00000094088_Ighv1-64	-0.276951909	10.8868825	0.95644203	0.32808494	0.5809402
ENSMUSG00000094124_Ighv1-74	-0.256971563	10.1565174	0.9407982	0.33207265	0.58263025
ENSMUSG00000105231_Iglj3	0.445552361	15.6411654	0.92919457	0.3350723	0.58263025
ENSMUSG00000079543_Igkv13-85	0.263507337	9.54336062	0.92231842	0.33686697	0.58263025
ENSMUSG00000094194_Ighv5-16	0.26056316	9.81106463	0.92019966	0.33742255	0.58263025
ENSMUSG00000094797_Igkv6-15	0.271672034	10.5824756	0.89871219	0.34312725	0.58882331
ENSMUSG00000076562_Igkv4-50	-0.24157172	10.1554421	0.87299281	0.3501281	0.597151
ENSMUSG00000095079_Igha	0.622972057	7.81467079	0.84951311	0.35669011	0.60358819
ENSMUSG00000096452_Ighv1-77	-0.290538096	9.18020159	0.84403681	0.35824479	0.60358819
ENSMUSG00000095592_Ighd5-7	-0.649330507	5.00801704	0.81973547	0.36525752	0.61064709
ENSMUSG00000095200_Ighv1-7	-0.260085741	10.1568173	0.80668439	0.36910233	0.61064709
ENSMUSG00000076564_Igkv12-46	-0.244995	9.76198843	0.80287265	0.37023588	0.61064709
ENSMUSG00000076594_Igkv6-13	0.272287622	9.23379843	0.79116689	0.37374752	0.61064709
ENSMUSG00000094505_Ighv8-6	0.472182721	6.48449882	0.78362143	0.37603586	0.61064709
ENSMUSG00000096805_Ighv9-1	0.259736521	10.0325341	0.77611813	0.37833099	0.61064709
ENSMUSG00000076523_Igkv15-103	0.241075804	9.57426012	0.77426322	0.37890141	0.61064709
ENSMUSG00000076577_Igkv8-30	0.251965001	9.6721933	0.77067926	0.380007	0.61064709
ENSMUSG00000095007_Igkv12-41	-0.233280903	9.65325154	0.73154946	0.39238141	0.62690823
ENSMUSG00000076538_Igkv13-84	0.308077386	8.80405391	0.69754677	0.40360925	0.6348118
ENSMUSG00000076525_Igkv1-99	-0.481681387	6.00351146	0.69718639	0.40373073	0.6348118
ENSMUSG00000105123_Ighv1-79	-0.491407698	5.3351094	0.69326074	0.40505749	0.6348118
ENSMUSG00000094006_Igkv4-59	0.21216088	10.3393884	0.68912478	0.40646223	0.6348118
ENSMUSG00000076709_Ighv1-47	-0.229032973	9.68623755	0.67518375	0.41125013	0.63707238
ENSMUSG00000076608_Igkj5	0.377073531	15.486796	0.67160409	0.41249291	0.63707238
ENSMUSG00000102524_Ighv1-2	-0.254762567	9.13168914	0.64354316	0.42243067	0.64881617
ENSMUSG00000118182_Gm50427	-0.442921533	6.01716163	0.61649833	0.43235151	0.65967763
ENSMUSG00000096638_Ighv2-9	0.234766291	9.19876365	0.60414049	0.43700259	0.65967763
ENSMUSG00000076615_Ighg3	-0.561090699	4.2901392	0.60395397	0.43707337	0.65967763
ENSMUSG00000104975_Iglj2	0.753599928	14.6635427	0.59375423	0.44097104	0.65967763
ENSMUSG00000095682_Igkv3-1	0.312557013	8.17792018	0.59272568	0.44136705	0.65967763
ENSMUSG00000076518_Igkv2-112	0.263756771	8.85308283	0.57980687	0.44638803	0.66361429
ENSMUSG00000094478_Igkv3-3	0.416937855	5.93614304	0.55996014	0.4542763	0.6665364
ENSMUSG00000104679_Igkv4-60	0.31541137	7.98018412	0.55786806	0.45512051	0.6665364
ENSMUSG00000105781_Igkv8-31	-0.558734137	3.96971686	0.55489596	0.45632408	0.6665364
ENSMUSG00000076591_Igkv8-16	0.20319541	10.7337683	0.55091499	0.45794407	0.6665364
ENSMUSG00000105547_Iglc3	0.339635191	15.1081855	0.54347477	0.46099623	0.66748412
ENSMUSG00000094315_Igkv4-78	0.248892032	8.89086979	0.5287429	0.46713585	0.67286925
ENSMUSG00000076617_Ighm	0.348183033	17.3514804	0.51663129	0.47228223	0.67677556
ENSMUSG00000095565_Ighv2-9-1	0.184258808	10.0521685	0.48983886	0.48399919	0.6861848
ENSMUSG00000106630_Igkv2-116	0.338122031	7.19670815	0.4792497	0.48876237	0.6861848
ENSMUSG00000106256_Igkv12-47	0.405542736	5.95829757	0.47543499	0.49049739	0.6861848
ENSMUSG00000104533_Igkv5-40-1	0.375379991	6.26698921	0.47494428	0.49072132	0.6861848

ENSMUSG00000076540_Igkv4-80	-0.281333597	8.40302214	0.47191206	0.49210884	0.6861848
ENSMUSG00000105606_Igkv2-109	0.245014201	8.63256957	0.4685432	0.49365813	0.6861848
ENSMUSG00000096715_Igkv3-4	0.198274599	9.23296256	0.45416514	0.5003636	0.68954181
ENSMUSG00000076710_Ighv1-49	-0.340366322	7.30480647	0.45274563	0.50103397	0.68954181
ENSMUSG00000076547_Igkv4-70	0.246010923	8.4109251	0.41864495	0.51761396	0.70727918
ENSMUSG00000096326_Ighv1-78	-0.317656718	11.8539838	0.41585893	0.51901062	0.70727918
ENSMUSG00000076609_Igkc	0.291222181	16.7502319	0.37662463	0.53941523	0.73149968
ENSMUSG00000094102_Ighv9-2	-0.170329386	9.43557026	0.34528378	0.55679508	0.75140308
ENSMUSG00000076512_Igkv9-123	0.271666192	7.48862618	0.33483406	0.56282639	0.75352941
ENSMUSG00000076556_Igkv4-57	0.154728655	10.5094676	0.33318133	0.56379179	0.75352941
ENSMUSG00000105432_Gm43218	0.148564981	10.3498374	0.30389826	0.58144882	0.76992341
ENSMUSG00000096833_Igkv4-55	0.14409403	10.0273607	0.30214115	0.58254321	0.76992341
ENSMUSG00000076549_Igkv4-68	-0.141048878	10.5684415	0.29922876	0.58436633	0.76992341
ENSMUSG00000076555_Igkv4-57-1	-0.262436959	7.71067725	0.2867031	0.59234105	0.77674911
ENSMUSG00000076552_Igkv4-61	-0.186117332	8.66037059	0.2755353	0.59964258	0.7826321
ENSMUSG00000094509_Ighv14-1	-0.189970368	8.69376466	0.26431842	0.60716874	0.78875192
ENSMUSG00000105605_Ighv1-86	-0.361815371	4.23368518	0.2555706	0.61317967	0.79285558
ENSMUSG00000076688_Ighv15-2	-0.149383467	9.48570109	0.24924357	0.61760821	0.79488464
ENSMUSG00000076563_Igkv5-48	-0.139570571	9.40559736	0.24004421	0.62417419	0.79963329
ENSMUSG00000106239_Gm9260	0.287001875	5.16385006	0.21634036	0.64184264	0.81408531
ENSMUSG00000102364_Ighv8-5	-0.161869405	8.99789554	0.21510133	0.64279808	0.81408531
ENSMUSG00000076655_Ighv4-1	-0.121571431	10.5384202	0.20891783	0.64761693	0.81408531
ENSMUSG00000105363_Igkv11-114	0.240278976	6.34800144	0.20184216	0.653238	0.81408531
ENSMUSG00000098814_Igkv19-93	0.124634903	11.778665	0.19659474	0.65748363	0.81408531
ENSMUSG00000096670_Ighv2-6	0.155854184	8.76412662	0.19629158	0.65773097	0.81408531
ENSMUSG00000076677_Ighv6-3	-0.120845008	11.0785089	0.19193504	0.66131095	0.81408531
ENSMUSG00000076618_Ighj4	-0.186959068	14.1884986	0.1901091	0.66282583	0.81408531
ENSMUSG00000094335_Igkv1-117	0.114967801	10.8050238	0.18932363	0.66348015	0.81408531
ENSMUSG00000094491_Igkv1-133	0.139406554	9.00614621	0.18474283	0.66732862	0.81408531
ENSMUSG00000093876_Ighd5-3	0.276261958	6.71784344	0.18210758	0.66956824	0.81408531
ENSMUSG00000076665_Ighv7-1	-0.120348616	9.50395325	0.1809064	0.67059545	0.81408531
ENSMUSG00000106387_Igkv4-73	-0.137780003	9.07078641	0.17708438	0.67389094	0.81452905
ENSMUSG00000076605_Igkj2	0.176885429	15.0225811	0.1568367	0.69208575	0.82639415
ENSMUSG00000076530_Igkv11-106	-0.18736427	7.45791349	0.15563006	0.6932121	0.82639415
ENSMUSG00000096632_Igkv9-124	-0.108582598	10.6291738	0.15468461	0.69409819	0.82639415
ENSMUSG00000095497_Igkv1-122	-0.12694671	9.0595918	0.14999015	0.69854477	0.82639415
ENSMUSG00000076587_Igkv6-20	-0.162908938	8.54752757	0.14996313	0.6985706	0.82639415
ENSMUSG00000096580_Igkv1-132	0.186139319	7.36224049	0.13940096	0.70887726	0.833794
ENSMUSG00000095753_Igkv4-53	-0.095777546	10.2749611	0.13745493	0.71082438	0.833794
ENSMUSG00000076541_Igkv4-79	0.121632105	8.9398792	0.12051305	0.72847871	0.85091211
ENSMUSG00000106405_Iglj3p	0.191227185	7.8650562	0.11487126	0.734665	0.85454758
ENSMUSG00000076573_Igkv1-35	0.209845865	4.76780112	0.11113314	0.73885774	0.85584355
ENSMUSG00000076576_Igkv6-32	-0.080550462	10.1078751	0.10026057	0.75151717	0.86499685
ENSMUSG00000105499_Igkv3-11	0.180479765	5.86457245	0.09896565	0.75307444	0.86499685
ENSMUSG00000076604_Igkj1	0.137928839	15.023093	0.09648083	0.75609437	0.86499685
ENSMUSG00000102381_Ighv8-7	0.18465745	5.86355052	0.08499911	0.77063371	0.8780171
ENSMUSG00000076548_Igkv4-69	-0.093399629	9.04179871	0.08116068	0.77573061	0.88021678
ENSMUSG00000096577_Ighv1-71	0.092346246	8.9955719	0.07753822	0.78066167	0.88037455
ENSMUSG00000095633_Igkv4-58	-0.094153211	9.70899731	0.076424	0.78220329	0.88037455
ENSMUSG00000076674_Ighv3-8	0.073361619	10.1637542	0.07076587	0.79022492	0.88581665
ENSMUSG00000095351_Igkv3-2	-0.065360601	10.2199948	0.06734516	0.79524219	0.88669299

ENSMUSG00000096767_Ighv1-62-3	-0.086999961	8.90785895	0.06591124	0.79738578	0.88669299
ENSMUSG00000076619_Ighj3	-0.101747365	14.1264234	0.05606735	0.81282324	0.89282621
ENSMUSG00000076571_Igkv5-37	-0.144791447	5.6630405	0.05508074	0.81444712	0.89282621
ENSMUSG00000095642_Ighv14-3	-0.059290379	10.1187981	0.05482572	0.81486936	0.89282621
ENSMUSG00000104742_Igkv9-128	-0.1921414	4.40318622	0.05429723	0.81574769	0.89282621
ENSMUSG00000076572_Igkv18-36	0.109422805	6.83412762	0.04664122	0.82901443	0.90073515
ENSMUSG00000091087_Ighv8-14	-0.185163176	2.99762025	0.046398	0.82945395	0.90073515
ENSMUSG00000094872_Igkv9-120	0.055844358	11.1235654	0.04353472	0.83472167	0.9029285
ENSMUSG00000076614_Ighg1	-0.138190691	3.9784508	0.03993818	0.8416015	0.90497231
ENSMUSG00000076536_Igkv4-86	-0.053758181	9.86559969	0.03916523	0.84312168	0.90497231
ENSMUSG00000096422_Igkv12-44	0.048912709	10.0671197	0.03659978	0.84828218	0.90700941
ENSMUSG00000094902_Igkv10-95	-0.049115534	10.055638	0.03466202	0.85230565	0.90781982
ENSMUSG00000093861_Igkv1-110	0.043759949	10.0831495	0.02841619	0.86613407	0.91902776
ENSMUSG00000106667_Igkv3-6	-0.105053915	4.13129792	0.02306254	0.87929456	0.92760834
ENSMUSG00000076508_Igkv17-127	0.03972502	11.4242513	0.02131987	0.88391092	0.92760834
ENSMUSG00000076586_Igkv8-21	0.04034298	9.71480056	0.01913409	0.88998276	0.92760834
ENSMUSG00000102901_Ighv5-1	0.071624359	6.66648494	0.01799088	0.89329972	0.92760834
ENSMUSG00000102535_Ighv1-41	-0.0722863	6.75060593	0.01768225	0.89421346	0.92760834
ENSMUSG00000076939_Iglv3	0.055580193	13.3533477	0.01767294	0.89424114	0.92760834
ENSMUSG00000103939_Ighv3-4	0.077553904	6.27476052	0.01499839	0.90252844	0.93272456
ENSMUSG00000096459_Ighv9-3	-0.029310753	12.2720502	0.00813855	0.92811722	0.95561699
ENSMUSG00000095210_Ighv5-9-1	0.022084337	9.94400644	0.00651178	0.93568404	0.959853
ENSMUSG00000076569_Igkv5-39	-0.013586154	11.1590319	0.00255699	0.95967083	0.98084003
ENSMUSG00000076545_Igkv4-72	-0.01190012	9.09632534	0.00147271	0.96938801	0.98401258
ENSMUSG00000106016_Igkv4-56	0.017566271	7.48033277	0.00142818	0.96985412	0.98401258
ENSMUSG00000095583_Ighv14-2	0.006201916	10.4775504	0.00052578	0.98170627	0.98975896
ENSMUSG00000076539_Igkv4-81	0.011010796	7.27230913	0.00047355	0.9826384	0.98975896
ENSMUSG00000094930_Igkv6-25	0.003325808	9.72610993	0.00015357	0.99011252	0.99366989
ENSMUSG00000076646_Ighv2-6-8	-0.003638691	7.72934721	6.29E-05	0.99366989	0.99366989
