# Supplementary material for: The chromatin reader Dido3 is a regulator of the gene network that controls B cell differentiation
Source: Cell Biosci. 2025 Apr 26;15:56. doi: 10.1186/s13578-025-01394-x (PMC12034202; doi:10.1186/s13578-025-01394-x)
Supplement: Supplementary file 6 — Additional file6 (PDF 73 KB) [file 13578_2025_1394_MOESM6_ESM.pdf]

Supplementary Table 4

ChIPseeker annotations of the H3K27me3 ChIP-seq data.

ID	#Peaks	Condition	Percentage of H3K27me3 peaks								
			Promoter	5'-UTR	3'-UTR	1st Exon	Other Exon	1st Intron	Other Intron	Down-stream	Distal Intergenic
HR45_S1	898	WT (replicate 1)	1.89	0.22	1.22	2.56	4.12	10.8	21.49	0.11	57.57
HR60_S4	894	WT (replicate 2)	2.01	0.34	1.12	2.46	3.47	10.51	21.92	-	58.16
HR61_S5	1212	WT (replicate 3)	1.89	0.25	1.24	2.72	3.96	9.65	22.52	-	57.76
HR56_S2	827	*Dido1*ΔE16 (replicate 1)	1.57	0.36	1.33	2.66	4.35	10.16	23.58	-	55.98
HR58_S3	1356	*Dido1*ΔE16 (replicate 2)	1.99	0.29	1.4	2.36	3.69	10.84	21.75	-	57.67
HR62_S6	973	*Dido1*ΔE16 (replicate 3)	1.85	0.31	1.34	2.36	4.01	9.97	23.23	-	56.94

Numbers in columns (promoter, 5'-UTR, 3'-UTR, 1st exon, other exon, 1st intron, other intron, downstream, distal intergenic) indicate the percentage of H3K27me3 peaks overlapping each genomic region. ID: identifier of biological replicates in the GEO dataset GSE272156. #Peaks: total number of H3K27me3 peaks detected with the callpeak function in MACS version 3.0.0b3. Condition: source of the LSK cells and their biological replicates in parenthesis.
