# Supplementary material for: The chromatin reader Dido3 is a regulator of the gene network that controls B cell differentiation
Source: Cell Biosci. 2025 Apr 26;15:56. doi: 10.1186/s13578-025-01394-x (PMC12034202; doi:10.1186/s13578-025-01394-x)
Supplement: Supplementary file 7 — Additional file7 (PDF 99 KB) [file 13578_2025_1394_MOESM7_ESM.pdf]

Supplementary Table 5

ChIPseeker annotations of the H3K27me3-enriched genomic regions.

H3K27me3 enrichment	Percentage of H3K27me3 peaks									
	Promoter	5'-UTR	3'-UTR	1st Exon	Other Exon	1st Intron	Other Intron	Downstream	Distal Intergenic	Gene symbols[(a)]
WT	1.72	-	3.45	-	-	8.62	15.52	-	70.69	Cd180, Cpeb4, Hmgxb4, Snrpe
*Dido1*ΔE16	-	-	-	-	-	6.82	20.45	-	72.73	Alx1,Atg7,Camk1d,Dclk2,Eya1, Marchf1,Snrpn,Spin4,Tet2,Zxda
Common regions	1.38	0.55	1.1	2.75	5.37	10.47	21.76	-	56.61	*Blk*,Cdk18,Cr2,Erdr1x,Ezh2,Fgfr2,*Gpm6a*, *Gramd2b(Gramd3)*,Hdac9,Hira,Jarid2,*Klra1*, Pou5f1-rs8,Snrpe,Srsf4,Trim30a,Zfp607a, Zfp746,Zfp825,Zfp945,Zfp958,Zfp980, Zfp982
**Control dataset annotations**										
Control IgG	1.36	0.34	1.9	2.07	3.11	12.16	28.11	0.07	50.87	
Control H3K4me3	32.21	0.99	2.21	3.01	4.08	10.18	17.06	0.09	30.16	

Numbers in columns (promoter, 5'-UTR, 3'-UTR, 1st exon, other exon, 1st intron, other intron, downstream, distal intergenic) indicate the percentage of H3K27me3 peaks overlapping each genomic region.
[(a)]The complete list of genes within 1.0 kb upstream to 200 bp downstream of transcriptional start sites is available in Supplementary File section. Text in italics and underline indicates genes that were differentially expressed in the RNA-seq of the pre-B cells.
