# Supplementary material for: The chromatin reader Dido3 is a regulator of the gene network that controls B cell differentiation
Source: Cell Biosci. 2025 Apr 26;15:56. doi: 10.1186/s13578-025-01394-x (PMC12034202; doi:10.1186/s13578-025-01394-x)
Supplement: Supplementary file 8 — Additional file8 (PDF 85 KB) [file 13578_2025_1394_MOESM8_ESM.pdf]

Supplementary Table 6

Summary of H3K27me3 ChIP-seq data in LSK cells.

Study	LSK cells	GEO dataset	#Peaks	H3K27me3 Ab	Bioinformatic pipeline[(a)]
This study	WT (replicate 3)	GSE272156	1212	Abcam (ab195477)	MACS3 callpeak -B --extsize 147 -g mm -q 0.05 –broad --broad-cutoff 0.1 --nomodel --extsize 147 MACS3 bdgdiff -g 73 -l 147 (b)
	*Dido1*dE16 (replicate 2)	GSE272156	1356		
Yang *et al*. Blood (2016) DOI:10.1182/blood-2015-11-679431	Ezh2-WT	GSM2091489	25541	Millipore (07-449)	Strand NGS Version 2.0 (Strand Genomics) with MACS method
	Ezh2-KO	GSM2091491	29626		
Hasemann *et al*. PLoS Genet (2014) DOI:10.1371/journal.pgen.1004079	Cebpa-WT	GSM1054811	239	Cell Signaling (C36B11)	MACS2 callpeak -g mm -p 0.001 --to-large
	Cebpa-KO	GSM1054814	597		

[(a)]For more details see https://github.com/macs3-project/MACS/wiki/Call-differential-binding-events
