# Supplementary material for: The chromatin reader Dido3 is a regulator of the gene network that controls B cell differentiation
Source: Cell Biosci. 2025 Apr 26;15:56. doi: 10.1186/s13578-025-01394-x (PMC12034202; doi:10.1186/s13578-025-01394-x)
Supplement: Supplementary file 9 — Additional file9 (PDF 69 KB) [file 13578_2025_1394_MOESM9_ESM.pdf]

Supplementary Table 7

ChIPseeker annotations of H3K27me3 ChIP-seq data in LSK cells.

Dataset	Promoter	5'-UTR	3'-UTR	1st Exon	Other Exon	1st Intron	Other Intron	Down-stream	Distal Intergenic
WT (replicate 3)	1.89	0.25	1.24	2.72	3.96	9.65	22.52	-	57.76
dE16 (replicate 2)	1.99	0.29	1.4	2.36	3.69	10.84	21.75	-	57.67
Ezh2-WT (GSM2091489)	0.99	0.05	0.48	0.52	0.24	12.15	25.87	0.1	59.59
Ezh2-KO (GSM2091491)	1.02	0.05	0.46	0.43	0.25	11.99	25.79	0.08	59.93
Cebpa-WT (GSM1054811)	1.25	-	0.84	8.37	3.35	12.55	24.27	-	49.37
Cebpa-KO (GSM1054814)	1.17	0.17	1.17	4.86	2.68	17.59	26.29	-	46.06

Numbers in columns (promoter, 5'-UTR, 3'-UTR, 1st exon, other exon, 1st intron, other intron, downstream, distal intergenic) indicate the percentage of H3K27me3 peaks overlapping each genomic region. Dataset: identifier of H3K27me3 ChIP-seq data and their GEO accession number in parenthesis.
