# Supplementary material for: The chromatin reader Dido3 is a regulator of the gene network that controls B cell differentiation
Source: Cell Biosci. 2025 Apr 26;15:56. doi: 10.1186/s13578-025-01394-x (PMC12034202; doi:10.1186/s13578-025-01394-x)
Supplement: Supplementary file 10 — Additional file10 (PDF 78 KB) [file 13578_2025_1394_MOESM10_ESM.pdf]

Supplementary Table 8

H3K27me3 peaks overlap enrichment analysis.

qSample[a]	GEO acc.	tSample[b]	qLen[c]	tLen[d]	N_OL[e]	N_OL(%qLen)[f]	p-value[g]	p.adjust[h]
WT (replicate 3)	GSM2091489	Ezh2-WT	1212	25541	107	8.8%	4.9×10^{-4}	5.9×10^{-4}
	GSM2091491	Ezh2-KO	1212	29626	106	8.7%	1.02×10^{-2}	1.02×10^{-2}
	GSM1054811	Cebpa-WT	1212	239	117	9.6%	9.9×10^{-5}	1.5×10^{-4}
	GSM1054814	Cebpa-KO	1212	597	146	**12%**	9.9×10^{-5}	**1.5×10^{-4}**
dE16 (replicate 2)	GSM2091489	Ezh2-WT	1356	25541	83	6.1%	1.3×10^{-2}	1.3×10^{-2}
	GSM2091491	Ezh2-KO	1356	29626	86	6.3%	1.3×10^{-2}	1.3×10^{-2}
	GSM1054811	Cebpa-WT	1356	239	117	8.6%	9.9×10^{-5}	1.5×10^{-4}
	GSM1054814	Cebpa-KO	1356	597	144	**10.6%**	9.9×10^{-5}	**1.5×10^{-4}**

[a]Query ChIP-seq sample, [b]Target ChIP-seq sample, [c]Number of query peaks, [d]Number of target peaks, [e]Number of overlapped peaks between query and target, [f]Percentage of overlapped peaks, [g]calculated p-value by ChIPseeker, [h]p-value correction (FDR) according to the Benjamini and Hochberg method. GEO database accession of tSample (GEO acc.). The values were obtained using the ChIPseeker command enrichPeakOverlap(queryPeak=file1, targetPeak=file-list, TxDb=TxDb.Mmusculus.UCSC.mm10.knownGene, pAdjustMethod="BH", nShuffle=10000, chainFile=NULL, verbose=FALSE) and a number of randomly permutations in the genomic locations of 10000.
