# Supplementary material for: The chromatin reader Dido3 is a regulator of the gene network that controls B cell differentiation
Source: Cell Biosci. 2025 Apr 26;15:56. doi: 10.1186/s13578-025-01394-x (PMC12034202; doi:10.1186/s13578-025-01394-x)
Supplement: Supplementary file 11 — Additional file11 (PDF 83 KB) [file 13578_2025_1394_MOESM11_ESM.pdf]

Supplementary Table 9

ChIP-seq H3K27me3 peaks and ATAC-seq chromatin-accessible regions overlap enrichment analysis.

qSample[a]	tSource	tSample[b]	qLen[c]	tLen[d]	N_OL[e]	N_OL(%qLen)[f]	p-value[g]	p.adjust[h]	Gene symbols
WT(replicate 3)	ATAC-seq	WT(open)	1212	26327	272	22.4%	1.8×10^{-2}	2.3×10^{-2}	(i)
		WT(open)/MUT(open)	1212	9197	219	18.1%	2.2×10^{-3}	3.7×10^{-3}	(i)
		MUT(open)	1212	9795	**333**	**27.5%**	3.9×10^{-4}	**9.9×10^{-4}**	(i)
		WT(open)/MUT(close)	1212	11007	7	0.6%	0.6	0.6	-
		WT(close)/MUT(open)	1212	81	**11**	**0.9%**	9.9×10^{-5}	**4.9×10^{-4}**	4930448K20Rik,Adam29,B230307C23Rik, Bcas1,G530011O06Rik,Gm12018,Gm17019, Mid1,Pdia3,Rimbp2,Speer4d
dE16(replicate 2)		WT(open)	1356	26327	276	20.3%	1.9×10^{-2}	2.4×10^{-2}	(i)
		WT(open)/MUT(open)	1356	9197	219	16.2%	2.9×10^{-3}	4.9×10^{-3}	(i)
		MUT(open)	1356	9795	**346**	**25.5%**	2.9×10^{-4}	**7.5×10^{-4}**	(i)
		WT(open)/MUT(close)	1356	11007	8	0.6%	0.6	0.6	-
		WT(close)/MUT(open)	1356	81	**14**	**1%**	9.9×10^{-5}	**4.9×10^{-4}**	4930448K20Rik,Adam29,1700064M15Rik, B230307C23Rik,Bcas1,G530011O06Rik, Gm12018,Gm17019,Gm21190,Mid1,Pdia3, Rimbp2,Speer4d

[a]Query ChIP-seq sample, [b]Target ChIP-seq sample, [c]Number of query peaks, [d]Number of target peaks, [e]Number of overlapped peaks between query and target, [f]Percentage of overlapped peaks, [g]calculated p-value by ChIPseeker,[h]p-value correction (FDR) according to the Benjamini and Hochberg method. [i]The complete list of genes within 1.0 kb upstream to 200 bp downstream of transcriptional start sites is available in Supplementary File section. The values were obtained using the ChIPseeker command enrichPeakOverlap(queryPeak=file1, targetPeak=file-list, TxDb=TxDb.Mmusculus.UCSC.mm10.knownGene, pAdjustMethod="BH", nShuffle=10000, chainFile=NULL, verbose=FALSE) and a number of randomly permutations in the genomic locations of 10000.
