# Supplementary material for: The chromatin reader Dido3 is a regulator of the gene network that controls B cell differentiation
Source: Cell Biosci. 2025 Apr 26;15:56. doi: 10.1186/s13578-025-01394-x (PMC12034202; doi:10.1186/s13578-025-01394-x)
Supplement: Supplementary file 12 — Additional file12 (PDF 78 KB) [file 13578_2025_1394_MOESM12_ESM.pdf]

Supplementary Table 10

ChIP-seq H3K4me3 peaks and ATAC-seq chromatin-accessible regions overlap enrichment analysis.

qSample[a]	tSource	tSample[b]	qLen(c)	tLen(d)	N_OL(e)	N_OL(%qLen)[f]	p-value[g]	p.adjust[h]
H3K4me3	ATAC-seq	WT(open)	47243	26327	17663	**37.4%**	9.9×10^{-5}	9.9×10^{-5}
H3K4me3		WT(open)/MUT(open)	47243	9197	8301	17.6%	9.9×10^{-5}	9.9×10^{-5}
H3K4me3		MUT(open)	47243	9795	8995	**19%**	9.9×10^{-5}	9.9×10^{-5}
H3K4me3		WT(open)/MUT(close)	47243	11007	4990	10.6%	9.9×10^{-5}	9.9×10^{-5}
H3K4me3		WT(close)/MUT(open)	47243	81	17	0.04%	9.9×10^{-5}	9.9×10^{-5}
H3K4me3	ChIP-seq (H3K27me3)	WT (enrichment)	47243	58	41	0.09%	9.9×10^{-5}	9.9×10^{-5}
H3K4me3		dE16 (enrichment)	47243	44	8	0.02%	9.9×10^{-5}	9.9×10^{-5}
H3K4me3		Common	47243	726	645	1.4%	9.9×10^{-5}	9.9×10^{-5}
H3K4me3	FANTOM5	F5.mm10.enhancers	47243	49797	7771	16.4%	9.9×10^{-5}	9.9×10^{-5}

[a]Query ChIP-seq sample, [b]Target ChIP-seq sample, [c]Number of query peaks, [d]Number of target peaks, [e]Number of overlapped peaks between query and target, [f]Percentage of overlapped peaks, [g]calculated p-value by ChIPseeker, [h]p-value correction
(FDR) according to the Benjamini and Hochberg method. The values were obtained using the ChIPseeker command enrichPeakOverlap(queryPeak=file1, targetPeak=file-list, TxDb=TxDb.Mmusculus.UCSC.mm10.knownGene, pAdjustMethod="BH", nShuffle=10000, chainFile=NULL, verbose=FALSE) and a number of randomly permutations in the genomic locations of 10000.
