# Supplementary material for: The chromatin reader Dido3 is a regulator of the gene network that controls B cell differentiation
Source: Cell Biosci. 2025 Apr 26;15:56. doi: 10.1186/s13578-025-01394-x (PMC12034202; doi:10.1186/s13578-025-01394-x)
Supplement: Supplementary file 13 — Additional file13 (PDF 102 KB) [file 13578_2025_1394_MOESM13_ESM.pdf]

Supplementary Table 11

List of bivalent genes with chromatin accessibility alterations and H3K4me3 marks in the promoter that are differently expressed.

Gene	ChIP-seq		ATAC-seq (WT/MUT)			RNA-seq		Description
	H3K4me3	H3K27me3	(cl/op)	(op/cl)	(op/op)	DOWN	UP	
Ctsh	1	0	0	0	1	1	0	cathepsin H
Atg16l2	1	0	0	0	1	1	0	autophagy related 16 like 2
Casz1	1	0	0	0	0	1	0	castor zinc finger 1
Rassf1	1	0	0	0	1	1	0	Ras association (RalGDS/AF-6) domain family member 1
**Sorcs2**	1	0	0	1	0	1	0	sortilin-related VPS10 domain containing receptor 2
**Reln**	1	0	0	1	0	1	0	Reelin
Mef2d	1	0	0	0	1	1	0	myocyte enhancer factor 2D
H2-Ab1	1	0	1	0	0	1	0	histocompatibility 2, class II antigen A, beta 1
Runx3	1	0	0	0	1	0	1	runt related transcription factor 3
Macf1	1	0	0	0	1	0	1	microtubule-actin crosslinking factor 1
Pdzd2	1	0	0	0	1	0	1	PDZ domain containing 2
Nfkb2	1	0	0	0	1	0	1	nuclear factor of kappa light polypeptide gene enhancer in B cells 2, p49/p100
Phactr2	1	0	0	1	0	0	1	phosphatase and actin regulator 2
Arhgap31	1	0	0	1	0	0	1	Rho GTPase activating protein 31
Sox18	1	0	0	1	0	0	1	SRY (sex determining region Y)-box 18
Adm	1	0	0	1	0	0	1	adrenomedullin
Cnnm2	1	0	0	0	1	0	1	cyclin M2
Lgr5	1	0	0	1	0	0	1	leucine rich repeat containing G protein coupled receptor 5
Nefh	1	0	0	1	0	0	1	neurofilament, heavy polypeptide
Cplx1	1	0	0	1	0	0	1	complexin 1
Plxna2	1	0	0	1	0	0	1	plexin A2
Heyl	1	0	0	1	0	0	1	hairy/enhancer-of-split related with YRPW motif-like
Tnfrsf21	1	0	0	0	1	0	1	tumor necrosis factor receptor superfamily, member 21
Nhsl2	1	0	0	1	0	0	1	NHS-like 2
Thrb	1	0	0	0	1	0	1	thyroid hormone receptor beta
Apba1	1	0	0	0	1	0	1	amyloid beta precursor protein binding family A member 1
Gpr157	1	0	0	0	1	0	1	G protein-coupled receptor 157
Lrrc8b	1	0	0	0	1	0	1	leucine rich repeat containing 8 family, member B
Arid5a	1	0	0	0	1	0	1	AT-rich interaction domain 5A
Epb41l4b	1	0	0	1	0	0	1	erythrocyte membrane protein band 4.1 like 4b
Begain	1	0	0	1	0	0	1	brain-enriched guanylate kinase-associated
Mypop	1	0	0	0	1	0	1	Myb-related transcription factor, partner of profilin
Ctif	1	0	0	1	0	0	1	CBP80/20-dependent translation initiation factor

The chromatin regions that are either close (cl) or open (op) are indicated using ATAC-seq data. One denotes the existence of a differentially expressed gene, a chromatin accessible region, or a histone mark, whereas cero denotes their absence.
